# From spikes to intercellular waves: Tuning intercellular calcium signaling dynamics modulates organ size control

**DOI:** 10.1371/journal.pcbi.1009543

**Published:** 2021-11-01

**Authors:** Dharsan K. Soundarrajan, Francisco J. Huizar, Ramezan Paravitorghabeh, Trent Robinett, Jeremiah J. Zartman

**Affiliations:** 1 Department of Chemical and Biomolecular Engineering, University of Notre Dame, South Bend, Indiana, United States of America; 2 Bioengineering Graduate Program, University of Notre Dame, South Bend, Indiana, United States of America; North Carolina State University, UNITED STATES

## Abstract

Information flow within and between cells depends significantly on calcium (Ca^2+^) signaling dynamics. However, the biophysical mechanisms that govern emergent patterns of Ca^2+^ signaling dynamics at the organ level remain elusive. Recent experimental studies in developing *Drosophila* wing imaginal discs demonstrate the emergence of four distinct patterns of Ca^2+^ activity: Ca^2+^ spikes, intercellular Ca^2+^ transients, tissue-level Ca^2+^ waves, and a global “fluttering” state. Here, we used a combination of computational modeling and experimental approaches to identify two different populations of cells within tissues that are connected by gap junction proteins. We term these two subpopulations “initiator cells,” defined by elevated levels of Phospholipase C (PLC) activity, and “standby cells,” which exhibit baseline activity. We found that the type and strength of hormonal stimulation and extent of gap junctional communication jointly determine the predominate class of Ca^2+^ signaling activity. Further, single-cell Ca^2+^ spikes are stimulated by insulin, while intercellular Ca^2+^ waves depend on Gαq activity. Our computational model successfully reproduces how the dynamics of Ca^2+^ transients varies during organ growth. Phenotypic analysis of perturbations to Gαq and insulin signaling support an integrated model of cytoplasmic Ca^2+^ as a dynamic reporter of overall tissue growth. Further, we show that perturbations to Ca^2+^ signaling tune the final size of organs. This work provides a platform to further study how organ size regulation emerges from the crosstalk between biochemical growth signals and heterogeneous cell signaling states.

## Author summary

Calcium (Ca^2+^) is a universal second messenger that regulates a myriad of cellular processes such as cell division, cell proliferation and apoptosis. Multiple patterns of Ca^2+^ signaling including single-cell spikes, multicellular Ca^2+^ transients, large-scale Ca^2+^ waves, and global “fluttering” have been observed in epithelial systems during organ development. Key molecular players and biophysical mechanisms involved in formation of these patterns during organ development are not well understood. In this work, we developed a generalized multicellular model of Ca^2+^ that captures all the key categories of Ca^2+^ activity as a function of key hormonal signals. Integration of model predictions and experiments reveals two subclasses of cell populations and demonstrates that Ca^2+^ signaling activity at the organ scale is defined by a general decrease in gap junction communication as an organ grows. Our experiments also reveal that a “goldilocks zone” of optimal Ca^2+^ activity is required to achieve optimal growth at the organ level.

## Introduction

Mechanisms of intercellular communication are critical during epithelial morphogenesis when cells communicate and coordinate their activities to generate functioning organs [1,2]. One modality of intercellular communication occurs through gap junctions (GJ), intercellular channels that permit direct cell-cell transfer of ions and other small molecules [3]. Calcium ions (Ca^2+^) act as second messengers that regulate a myriad of cellular processes such as proliferation, differentiation, transcription, metabolism, cellular motility, fertilization, and neuronal communication [4–13]. Ca^2+^ signaling also regulates developmental processes at the multicellular level. For instance, Ca^2+^ signaling has been shown to regulate scale development in butterfly wings [14]. It also mediates autophagic and apoptotic processes required for hearing acquisition in the developing cochlea [15–17]. However, a systems-level description of Ca^2+^ signaling during organ development is lacking.

**Fig 1:**
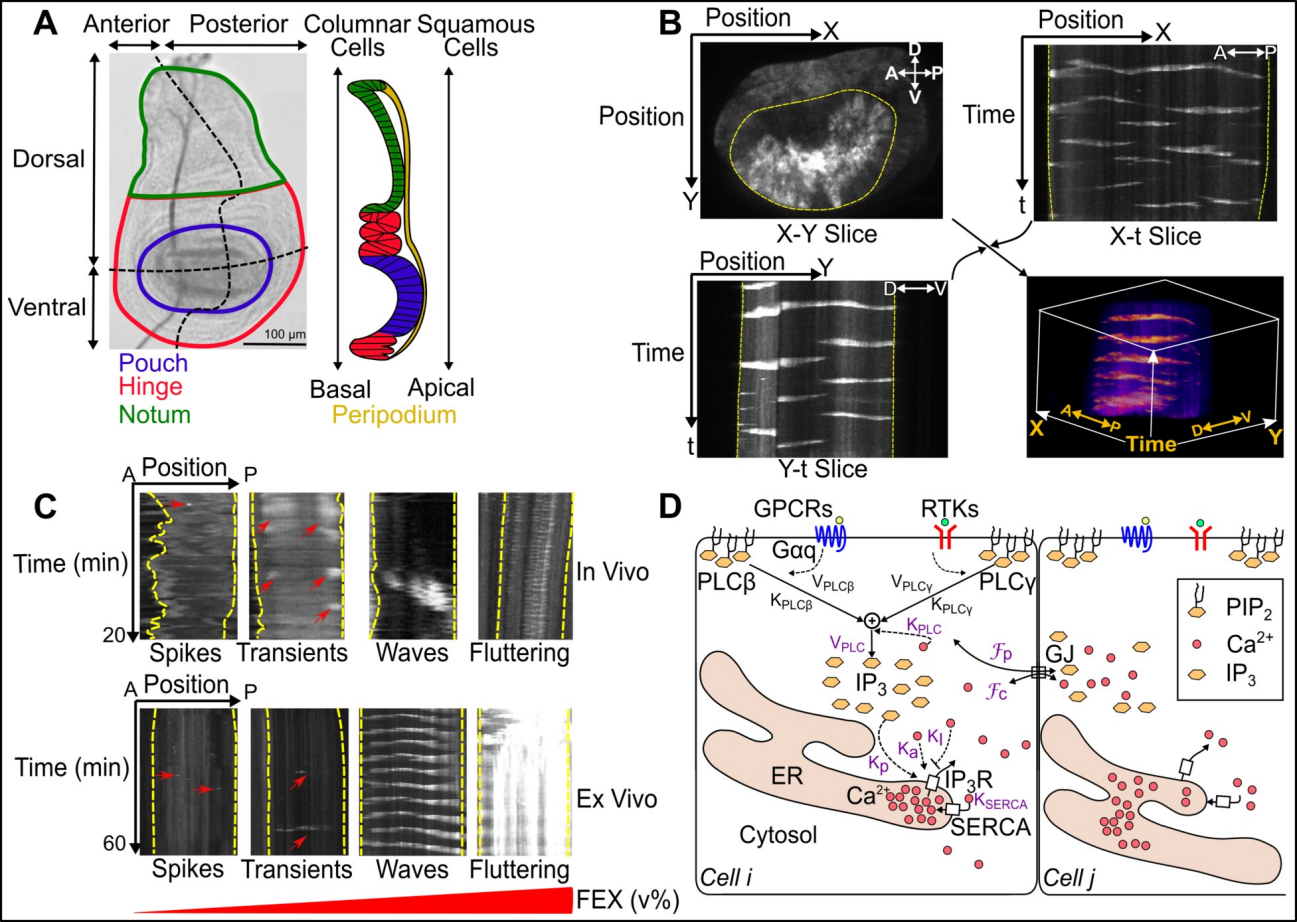
Multicellular Ca^2+^ signaling in a developing organ. **(A)** (*Left panel*) Image of third instar *Drosophila* wing imaginal disc. The larval wing disc includes main four regions: pouch (blue), hinge (red), notum (green) and peripodium (yellow). The pouch cells are the region of interest for this study. (*Right panel*) A schematic of the side view of the wing disc showing the peripodial membrane composed of squamous epithelial cells. **(B)** Kymographs illustrate two-dimensional slices of three-dimensional (X, Y and t planes respectively) spatiotemporal signaling. Ca^2+^ signaling activity is related to the fluorescence intensity of the Ca^2+^ sensor, GCaMP6f. A view of the X-Y plane (top left), the X-t plane (top right), and Y-t plane (bottom left) are combined to illustrate a 3D view of the signaling activity (bottom right). Yellow dashed lines indicate the pouch region. X coordinates roughly correspond to the A/P direction of the wing disc pouch whereas the Y coordinate principally describes the D/V axis of the pouch. **(C)** Four classes of Ca^2+^ signaling patterns are observed both in vivo and ex vivo: single-cell spikes, intercellular transient, intercellular waves and fluttering [19]. In ex vivo cultures, the occurrence of these patterns depend on the concentration of fly extract added to the culture media. Red arrows highlight subtle cellular Ca^2+^ activity. **(D)** Major components of Ca^2+^ toolkit: G protein–coupled receptors (GPCRs), receptor tyrosine kinase (RTKs), gap junctions (GJ), Inositol trisphosphate (IP_3_), diacylglycerol (DAG), Phospholipase C (PLC), Phosphatidylinositol 4,5-bisphosphate (PIP_2_), sarco/endoplasmic reticulum Ca^2+^-ATPase (SERCA) and IP_3_ receptors (IP_3_R). Parameters for our computational model are denoted in purple. K_PLC_ and V_PLC_ values are lumped activity parameters that are describe the stimulus-dependent activity of specific PLC isoforms, specifically PLCγ downstream of the insulin receptor and PLCβ downstream of GPCR signaling.

A major challenge in reverse engineering Ca^2+^ signaling during organ development is the lack of an in vivo model system to identify how cells interpret and integrate information across the broad range of input molecules that dynamically vary concentrations of cytosolic Ca^2+^ ions. In particular, it remains unclear how single-cell Ca^2+^ dynamics are coordinated to regulate tissue-level Ca^2+^ patterns. To overcome these challenges, we developed a computational model based on a realistic geometry of epithelial cells to model Ca^2+^ signaling in the *Drosophila* wing imaginal disc. The *Drosophila* wing imaginal disc is an experimentally amenable system for investigating systems-level regulation of cell signaling (Fig 1A) [18–20]. Overall, *Drosophila* wing imaginal discs are a premier system to gain insights into several organ-intrinsic and organ-extrinsic mechanisms that control organ growth [21–26].

Previous experimental investigations revealed four classes of Ca^2+^ signaling activity in the developing wing disc: single-cell Ca^2+^ spikes, multicellular transients, intercellular waves, and global fluttering (Fig 1B and 1C). We have shown recently that the patterns depend on the strength of agonist stimulation [19]. We and others have previously reported that Ca^2+^ patterns observed in the wing disc are dependent on phospholipase C (PLC) and the inositol trisphosphate receptor (IP_3_R) pathway mediated by gap junctional communication [18–20]. In non-excitable cells, stimulation of receptors in the cell surface results in activation of PLCs to generate IP_3_, which binds to and activates IP_3_R (Fig 1D) [6]. Upon binding, IP_3_Rs channel Ca^2+^ from the endoplasmic reticulum (ER) to the cytosolic space [6,27,28]. However, the specific receptors involved in stimulation of PLCs in the *Drosophila* wing imaginal disc remain to be more fully defined. The key physical/chemical parameters and their interactions that define multicellular Ca^2+^ dynamics in response to agonist stimulation is not fully characterized. How these different spatiotemporal modes of signaling encode information from upstream signals that impact downstream cellular processes during organ development is poorly understood. We have previously shown that inhibition of Ca^2+^ regulators of IP_3_, including PLC21C, Gαq and the gap junction protein Innexin 2 (Inx2) in the wing disc results in reduction in size of adult wing blade [19]. Whether changes to multiscale Ca^2+^ signaling patterns in wing disc alters overall adult blade wing size remains unknown.

Overall, the computational models of calcium signaling in developing epithelial systems have received sparse attention to date. Here, we report the necessary conditions to generate the full spectrum of experimentally observed spatiotemporal patterns by employing a computational modeling approach. To do so, we built a geometrically accurate 2D-model of a wing disc based on experimental data. We discovered that in silico replication of wing disc Ca^2+^ patterns requires two distinct classes of cells, which we term as “initiator cells” and “standby cells.” Here, we show how the standby cells organize themselves with respect to a Hopf bifurcation threshold of the model’s V_PLC_ parameter, and how the range of standby cell V_PLC_ values determine the final patterns of Ca^2+^ signaling. Next, computational simulations and experiments demonstrate that gap junction communication alters Ca^2+^ signaling response resulting in more Ca^2+^ spikes in the absence of external stimuli. Finally, we provide computational and experimental evidence for the role of Ca^2+^ signaling in imaginal disc morphogenesis. Our findings suggest a “goldilocks zone” of integrated Ca^2+^ signaling where lower levels of Ca^2+^ is correlated with reduced organ growth and higher levels of Ca^2+^ is also correlated with reduced growth dependent upon the stimulus triggering the Ca^2+^ signal. Overall, we identify crucial crosstalk between biochemical growth signals, such as insulin and Gαq, and heterogeneous cell signaling states during the growth of an organ.

## Methods

### Computational model

Several mathematical models have been proposed to describe intra- and intercellular Ca^2+^ wave propagation [29]. Here we extended a previously formulated model reported by Politi and colleagues that described single-cell Ca^2+^ oscillations observed in Chinese hamster ovarian (CHO) cells [30]. This model serves as a baseline model in cells for IP_3_R-mediated Ca^2+^ signaling in the *Drosophila* wing disc. This model accounts for the formation and degradation of IP_3_, Ca^2+^ flux across the endoplasmic reticulum (ER) through IP_3_R, and sarco/endoplasmic reticulum Ca^2+^-ATPase (SERCA), IP_3_R, and ER Ca^2+^ dynamics. The model consists of four state variables: cytosolic IP_3_ (*p*), cytosolic Ca^2+^ (*c*), the ER Ca^2+^ concentration (*s*), and the fraction of IP_3_ receptors that have not been inactivated by Ca^2+^ (*r*).

### IP_3_ dynamics

IP_3_ is generated in the cytosol by phospholipases (PLC) [31]. The *Drosophila* genome consists of three PLC genes. They include *PLC21C* and *norpA*, which are related to the PLCβ1–4 subfamily of *Homo sapien* homologs, and a single PLCγ (*sl*) [32]. While different classes of PLC can hydrolyze PI(4,5)P_2_ to generate IP_3_ and DAG, they are activated by different receptors on the cell surface. For instance, PLCβ homolog PLC21C is activated by the heterotrimeric G-protein αq subunit in response to G-protein receptor signaling [33]. On the other hand, PLCγ is recruited via its SH2 domain to activated receptor tyrosine kinase, such as the insulin receptor, at the plasma membrane [32]. In our model, we describe all the combined production of IP_3_ as dependent on the total PLC activity of the cell:

$$v_{{PLC}_{i}}=V_{PLC}\frac{c_{i}^{2}}{K_{PLC}^{2}+c_{i}^{2}},$$ (1)

where V_PLC_ describes the maximal production rate of IP_3_, and K_PLC_ describes the sensitivity of PLC to Ca^2+^. The parameter V_PLC_ depends on agonist concentration, we assume that V_PLC_ describes a summed activity of PLCs activated by upstream receptors.

Our model also considers degradation of IP_3_ by other factors such as IP_3_ kinases, which converts IP_3_ to IP_4_. We generalize the degradation of IP_3_ using first order kinetics. Collectively, the equation describing the dynamics of IP_3_ in a cell is:

$$\frac{{dp}_{i}}{dt}=J_{p_{i}}+v_{{PLC}_{i}}-k_{5,P}p_{i},$$ (2)

where $J_{p_{i}}$ is the flux of cell *i* IP_3_ through gap junction communication and *k*_*5*,*P*_ is the IP_3_ dephosphorylation rate constant. We assume that IP_3_ diffuses from one cell to the adjacent cells through gap junctional coupling. We model the flux through gap junctions (GJs) using the following equation:

$$J_{p_{i}}\approx F_{p}\left[\sum_{j\in N_{i}}p_{j}l_{ij}-p_{j}(\sum_{j\in N_{j}}l_{ij})\right],$$ (3)

where F_p_ refers to the IP_3_ permeability of the gap junctions, *p*_*j*_ refers to the IP_3_ concentration in neighboring cell *j* and *l*_*ij*_ refers to the length of cell boundary shared by cells *i* and *j* respectively. We assume that the intracellular diffusion of IP_3_ is fast relative to the diffusion of IP_3_ between cells through GJs. Consequently, we have neglected terms that describe intracellular diffusion.

### Ca^2+^ dynamics

Ca^2+^ is released through IP_3_R from the ER. Similarly, cytosolic Ca^2+^ is pumped into the ER using SERCA pumps. In many cell types, Ca^2+^ is also pumped out from the cytosol to the extracellular space through the plasma membrane. In our model, we ignore the flux of Ca^2+^ through the cell’s plasma membrane, and we only consider the transport of Ca^2+^ from the ER to cytosol. This assumption is based on our previous experimental studies showing that the observed Ca^2+^ dynamics in the wing disc are due to the IP_3_ mediated Ca^2+^ release through IP_3_R from ER [18,19]. To describe the IP_3_R dynamics, we followed Politi et al.’s derivation [30,33,34]. Thus, the dynamics of cytosolic Ca^2+^ in a cell is given by:

$$\frac{{dc}_{i}}{dt}=J_{c_{i}}+\left[k_{1}{\left(r_{i}.\frac{c_{i}}{K_{a}+c_{i}}\frac{p_{i}}{K_{p}+p_{i}}\right)}^{3}+k_{2}\right](s_{i}-c_{i})-V_{SERCA}\frac{c_{i}^{2}}{c_{i}^{2}+K_{SERCA}^{2}},$$ (4)

where *k*_1_ refers to maximal rate of Ca^2+^ release, *K*_*a*_ is the rate constant characterizing Ca^2+^ binding to activating site in IP_3_R, *K*_*p*_ is the rate constant characterizing IP_3_ binding to IP_3_R, *k*_2_ refers to Ca^2+^ leak out of ER, *V*_*SERCA*_ is the maximum rate of SERCA pump and *K*_*SERCA*_ is the half activation constant. We assume that Ca^2+^ acts as both a positive and negative regulator of IP_3_R which is consistent with experimental observations of the single channel properties of the wild-type *Drosophila* receptor that has been studied using a lipid bilayer reconstitution technique [35]. Similar to IP_3_, we also model diffusion of Ca^2+^ through GJs by the following equation:

$$J_{c_{i}}\approx F_{c}\left[\sum_{j\in N_{i}}c_{j}l_{ij}-c_{j}(\sum_{j\in N_{j}}l_{ij})\right],$$ (5)

where F_c_ refers to the permeability of Ca^2+^ through GJs, *c*_*j*_ refers to the concentration of Ca^2+^ in neighboring cell *j* and *l*_*ij*_ refers to the length of cell boundary shared by cells *i* and *j* respectively.

Similarly, we describe the dynamics of Ca^2+^ concentration in the ER of a cell as:

$$s_{i}(t)=\frac{c_{tot}-c_{i}(t)}{\beta },$$ (6)

where *s*_*i*_ is the Ca^2+^ concentration in the ER of the cell and *β* is the ratio of effective cytoplasmic and effective ER volume, *c*_*i*_ refers to the cytosolic Ca^2+^ concentration in the cell and *c*_*tot*_ refers to the total Ca^2+^ concentration in the cell which includes both ER and the cytosol. We modified the rate of IP_3_R inactivation term from the Politi model, *r*, to replicate our experimental Ca^2+^ dynamics. The modified equation is described below:

$$\frac{{dr}_{i}}{dt}=\frac{1}{\tau_{max}}\frac{k_{\tau }^{4}+c_{i}^{4}}{k_{\tau }^{4}}(1-r_{i}\frac{K_{r}+c_{i}}{K_{r}})$$

A similar modification to the rate of inactivation term, *r*, has been proposed previously [36].

### IP_3_R dynamics

We assume that the Ca^2+^ binding to the inactivating site on the IP_3_R is a slow process [36]. Consequently, we consider the dynamics of IP_3_R inactivation by Ca^2+^ in a cell as a separate differential equation given below:

$$\tau_{r}\frac{{dr}_{i}}{dt}=\left[1-r_{i}\frac{\left(K_{r}+c_{i}\right)}{K_{r}}\right],$$ (7)

where *r*_*i*_ refers to the fraction of IP_3_Rs of cell *i* that are not inactivated by Ca^2+^, *K*_*r*_ refers to the binding coefficient characterizing Ca^2+^ binding to the inactive site on the IP_3_R and *τ*_*r*_ refers to the characteristic time of IP_3_R inactivation.

## Results

### The relative rate of IP_3_ production governs transitions between classes of spatiotemporal Ca^2+^ patterns at the tissue level

Multiple spatiotemporal classes of Ca^2+^ activity are observed in vivo and ex vivo in the wing disc. However, an understanding of how this activity is regulated requires developing a systems-level description. To summarize, these include: (i) single-cell Ca^2+^ spikes, (ii) intercellular Ca^2+^ transients (ICTs), (iii) intercellular Ca^2+^ waves (ICWs), and a (iv) global fluttering phenomenon (Fig 1C and Table 1 and S1–S8 Movies) [18–20]. The frequencies of these observed classes are dependent on the age of the larvae in both in vivo and ex vivo experiments. Younger larva with smaller discs (4–5 days after egg laying) exhibit higher occurrences of ICWs and fluttering states while older, larger larval discs (6–8 days after egg laying) predominantly show ICTs and spikes [19]. For ex vivo cultures, the transition from limited to tissue-wide Ca^2+^ signaling activity depends on the amount of fly extract (FEX) added to the culture. Low concentrations of FEX stimulated Ca^2+^ spikes. Progressively increasing levels of FEX resulted in ICTs, ICWs, and eventually fluttering. Further, FEX-stimulated Ca^2+^ dynamics is based on IP_3_R-based release of Ca^2+^ from the ER to the cytosol as shown in Fig 1D [19].

**Table 1:** Baseline parameters used in the model.

Parameter	Description	Value
*k*_5,*P*_	IP_3_ dephosphorylation rate constant	0.66 s^-1^
*K*_*PLC*_	Half activation of PLC	0.2 μM
*V*_*PLC*_	Maximum production rate of PLC	0.1–1.5 μM s^-1^
*β*	Ratio of effective volumes ER/cytosol	0.185
*V*_*SERCA*_	Maximum SERCA pump rate	0.9 μM s^-1^
*K*_*SERCA*_	Half activation constant for SERCA pump	0.1 μM
*k*_1_	Maximum rate of Ca^2+^ release from IP_3_R	1.11 s^-1^
*k*_2_	Ca^2+^ leak from ER	0.0203 s^-1^
*K*_*a*_	Ca^2+^ binding to activating site of IP_3_R	0.08 μM
*K*_*r*_	Ca^2+^ binding to inactivating site of IP_3_R	0.4 μM
*K*_*p*_	IP_3_ binding to IP_3_R	0.13 μM
*τ*_*max*_	Maximum time constant of IP_3_R inactivation	800 s^-1^
*k*_*τ*_	Ca^2+^ dependent rate of IP_3_R inactivation	1.5 μM
*F*_*p*_	GJ permeability for IP_3_	0.005 μM^2^ s^-1^
*F*_*c*_	GJ permeability for Ca^2+^	0.0005 μM^2^ s^-1^
*C*_*tot*_	Total Ca^2+^ concentration in ER and cytosol	2 μM

Most baseline parameters were adopted from Politi and colleagues [30]. V_PLC_ was varied in this report in order to investigate the effects of IP_3_ production on spatiotemporal calcium patterns.

These experimental findings motivated us to ask what specific cellular properties of the wing disc result in the emergence of these distinct patterns. To systematically investigate the underlying principles governing the emergence of these patterns, we formulated a two-dimensional image-based, geometrically realistic computational model of Ca^2+^ signaling in the wing disc pouch where columnar epithelial cells are connected by gap junctions (S1 Fig), [37–40]. Image-based modelling enables the holistic characterization of molecular mechanisms and tissue dynamics during organogenesis [41]. Given the near universal conservation of the Ca^2+^ signaling pathway across model systems [27,28], the baseline single-cell Ca^2+^ model in our study was adapted from Politi and colleagues [30]. The model equations, biological relevance and descriptions of the parameters are shown in Fig 1D and Table 1.

**Fig 2:**
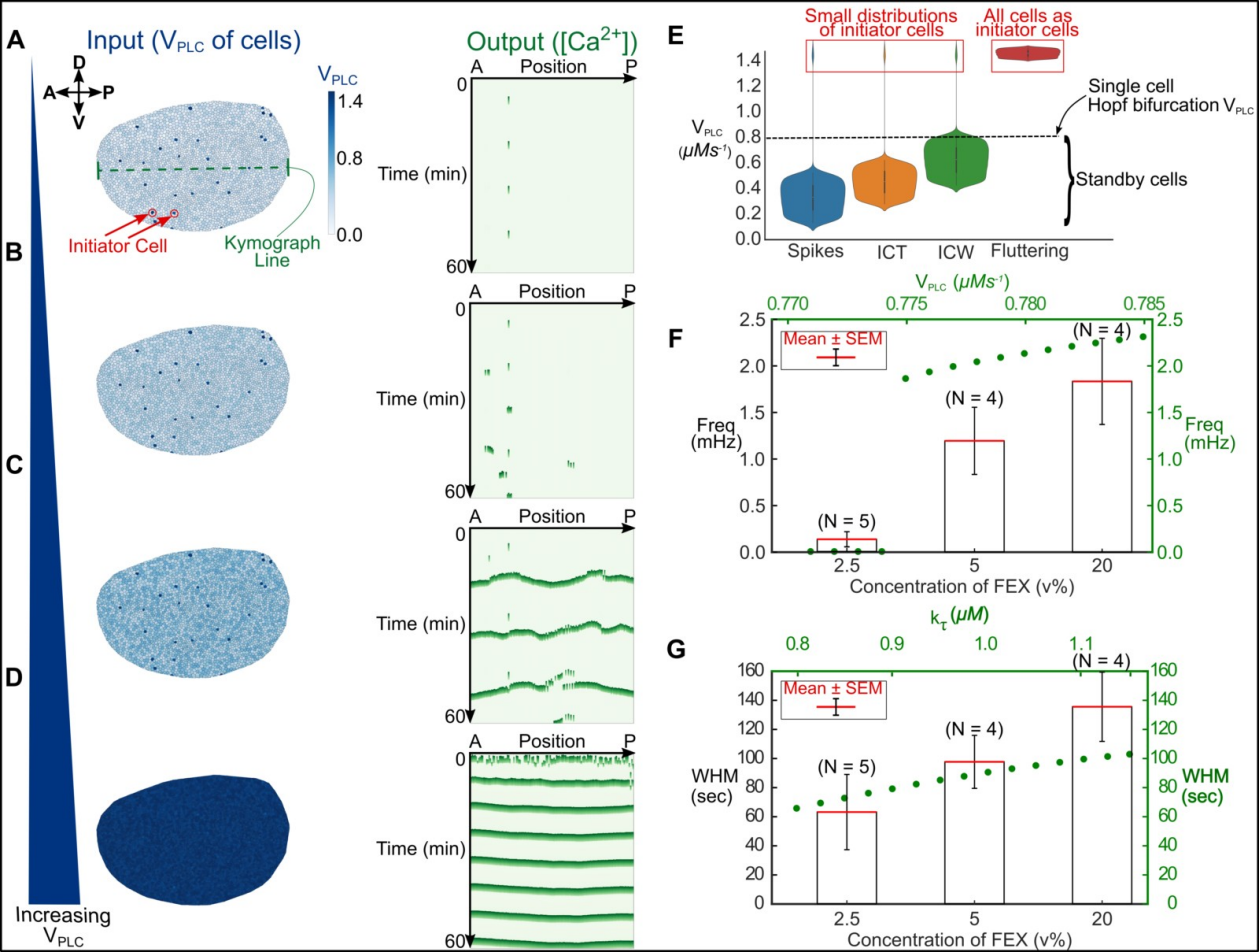
**The level of hormonal stimulation governs the spatial extent of intercellular Ca**^**2+**^ **communication. (A-D)** Computer simulations recapitulating the key classes of multicellular Ca^2+^ activity observed in vivo and ex vivo. **(A)** When the majority of cells have V_PLC_ values below the Hopf bifurcation threshold (*left*), single-cell Ca^2+^ spikes are seen (*right*). Initiator cells (red arrows) are cells with V_PLC_ values set between 1.4 and 1.5 in the simulation. A line through the A/P direction (green) demonstrates where the kymograph line is drawn that produces the simulated tissue’s corresponding kymograph. **(B)** Intercellular Ca^2+^ transients are observed (*right*) as the distribution of V_PLC_ in cells is increased (*left*). **(C)** A further increase in V_PLC_ (*left*) results in the emergence of periodic intercellular Ca^2+^ waves (*right*). **(D)** “Fluttering” occurs (*right*) when V_PLC_ levels in all of the cells in the disc is above Hopf bifurcation (*left*). **(E)** Quantification of V_PLC_ distribution in the initiator and the standby cells for each of the classes of Ca^2+^ signaling activity. The first three Ca^2+^ signaling classes have a small distribution of initiator cells (red box) that are necessary for signal initiation. The dashed line indicates the threshold of the V_PLC_ parameter that permits Ca^2+^ oscillations. **(F-G)** The single-cell version of our model predicts that the frequency and width at half maximum (WHM) of Ca^2+^ oscillations is altered by varying V_PLC_ and *k*_*τ*_. This prediction matches the WHM of Ca^2+^ activity observed in ex vivo discs cultured with variable concentrations of fly extract. Error bars are reported as standard error of the means (SEM).

To reproduce the four distinct patterns in silico, we varied the V_PLC_ parameter progressively in individual cells across a range of values (Table 1). Given that our patterns were dependent on the concentration of FEX, we varied V_PLC_ as a lumped-parameter representing the level of agonist stimulation (Fig 1D). We and others have demonstrated by FEX, which contains a mixture of agonists, stimulates PLC activity through GPCR and RTK signaling [18,19]. From these results, it can be inferred that the stimulation of PLC activity from FEX would subsequently increase the maximal rate of production of IP_3_ (higher V_PLC_). Thus, V_PLC_ is not *directly* a parameter representing the concentration of FEX, but is a parameter that describes the net activity of PLC through FEX stimulation of upstream receptors. The computational model successfully reproduced the four different spatiotemporal classes of Ca^2+^ signaling dynamics observed in vivo and ex vivo (Fig 2A–2D and S9–S12 Movies). Interestingly, we discovered that the formation of these patterns is dependent on the number of cells in the simulated tissue having a V_PLC_ value below, above, or equal to the Hopf bifurcation threshold for single-cells (V_PLC_ = 0.774) (Fig 2E). The Hopf threshold was identified from a single-cell version of the model wherein Ca^2+^ oscillations occur in the cell when V_PLC_ is at or above the value of 0.774 (Figs 2F and S2A and S3A). Simulated cells that have a V_PLC_ value above the Hopf threshold, in the absence of agonist stimulation, are termed “initiator cells” and are posed to exhibit high levels of IP_3_ production. Neighboring simulated cells with V_PLC_ values below the Hopf threshold are termed “standby cells” that receive a signal from initiator cells to propagate a signal. For instance, if a majority of standby cells have V_PLC_ values significantly below the critical Hopf bifurcation threshold (standby cell V_PLC_ randomly uniformly distributed between 0.1–0.5), single-cell Ca^2+^ spikes occur only where initiator cells oscillate (Fig 2A and 2E). When we increased standby cell V_PLC_ values close to the lower end of the Hopf bifurcation point (Fig 2B and 2E), we noticed the formation of ICTs (standby cell V_PLC_ randomly uniformly distributed between 0.25–0.60). Finally, we observed the formation of ICWs (standby cell V_PLC_ randomly uniformly distributed between 0.4–0.8) and fluttering phenotypes (standby cell V_PLC_ randomly uniformly distributed between 1.4–1.5) (Fig 2C–2E) for cases when the majority of cells in the system were assigned a V_PLC_ close to or above the bifurcation threshold, thereby placing more cells in an initiator state. In the absence of initiator cells, Ca^2+^ activity is not observed. This suggests that initiator cells are necessary for the formation of Ca^2+^ transients in developing tissues.

The “single-cell” version of the model predicts that differences in Ca^2+^ signal amplitude and frequency are tunable by varying the V_PLC_ and *k*_*τ*_ parameters (Figs 2F and 2G and S2). An output Ca^2+^ signal was observably tunable with V_PLC_ variations in replication of the four distinct patterns (Fig 2A–2E). Because the single-cell model predicted Ca^2+^ signal perturbations by tuning *k*_*τ*_, we sought to investigate how the characteristic time associated with inactivation of IP_3_R would influence tissue-scale signaling. To do this, we performed a sensitivity analysis of the two model parameters that influence characteristic time associated with inactivation of IP_3_R: *k*_*τ*_ and *τ*_*max*_. Two-dimensional computational model simulations of the tissue were performed by varying parameter values as a percentage of their baseline values (Table 1) while holding other parameters constant. The baseline parameter set was selected such that the simulation generated intercellular Ca^2+^ waves (S4 Fig, red box). This was done by each baseline simulation having the exact same V_PLC_ profile for standby and initiator cells with standby cell V_PLC_ values being uniformly random distributed between 0.7 and 1.0 and parameter values being set to those detailed in Table 1. Reducing *k*_*τ*_ leads to a narrower width at half maximum (WHM) of Ca^2+^ transients (S4A Fig). A similar result is observed where a reduction of *τ*_*max*_ results in decreased WHM, whereas increased *τ*_*max*_ increased WHM (S4B Fig). Further, a decrease in the *τ*_*max*_ parameter increased the frequency of signals in the simulated tissue, while an increase reduced the frequency of Ca^2+^ transients. This suggests that the system is more sensitive to *τ*_*max*_, and variations in *τ*_*max*_ have much greater impact in how quickly the system responds to stimulus.

These results indicate that tissue-level Ca^2+^ patterns depend on the spatial distribution of cell states defined by their maximal IP_3_ production rates, in relation to the effective tissue-level Hopf bifurcation threshold. Further, other key parameters in the model can be modified in a manner to allow tunable Ca^2+^ signaling patterns. A systematic sensitivity analysis of other parameters in the model demonstrated perturbations to Ca^2+^ signal strength, frequency, duration, and propagation (S4–S6 Figs). Further exploration and quantitative analysis of all parameters could allow an avenue to explore how tunable Ca^2+^ signaling can induce a desired physiological outcome.

### GJ communication limits Ca^2+^ spikes in the absence of hormonal stimulation

**Fig 3:**
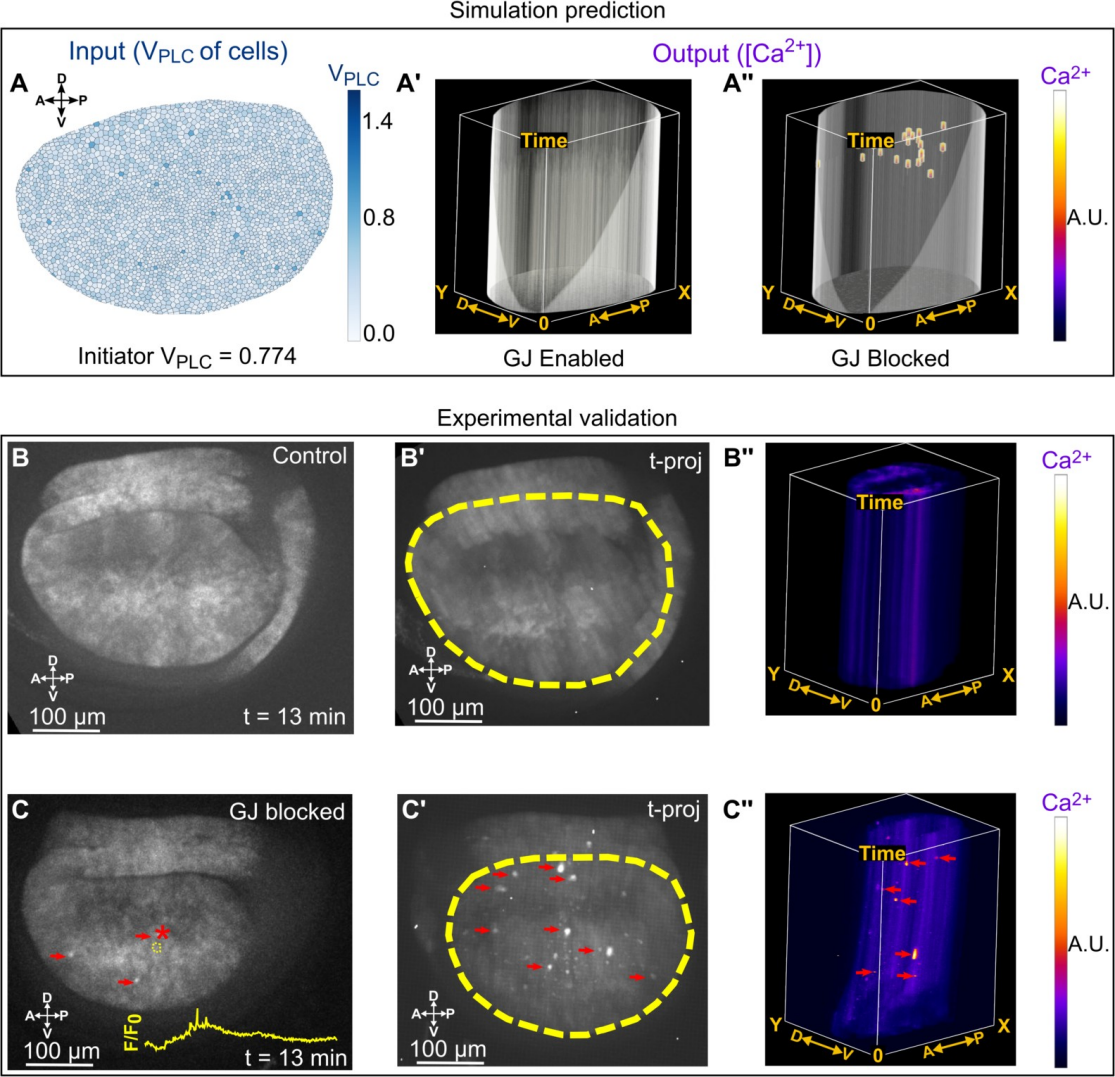
Gap junction (GJ) communication decreases the proportion of cells exhibiting Ca^2+^ spikes. **(A)** Simulation of Ca^2+^ signaling in wing disc where the V_PLC_ values of initiator cells were set to the Hopf bifurcation threshold 0.774, and standby cell V_PLC_ values were randomly distributed between 0.1 and 0.5. **(A’)** Allowing GJ communication by letting permeability of IP_3_ (F_p_) and Ca^2+^ (F_c_) be 0.005 μM^2^ s^-1^ and 0.0005 μM^2^ s^-1^ respectively, resulted in no Ca^2+^ activity (GJ Enabled). **(A”)** F_p_ and F_c_ were set to zero to simulate inhibition of GJ communication (GJ Blocked). Inhibition of GJ communication resulted in Ca^2+^ spike activity. A.U. indicates arbitrary units that correspond to the intensity of the signal. X coordinates roughly correspond to the A/P direction of the wing disc pouch whereas the Y coordinate principally describes the D/V axis of the pouch. **(B)** Ex vivo time lapses of nub>GCaMP6f x UAS-RyR^RNAi^ (control) wing discs in Grace’s low ecdysone media were generated by imaging for 1 h at 10 sec intervals. Under this condition, we observed no Ca^2+^ activity in the wing disc cells. **(B’)** Time-projection of the time lapse stack. The wing disc boundary is indicated with the yellow dashed line. **(B”)** A kymograph generated further demonstrates no instances of Ca^2+^ activity. **(C)** GJ communication was blocked by culturing wing discs in Grace’s low ecdysone media with 100 mM of Carbenoxolone (CBX). Instances of spike activity are denoted by red arrows. The intensity of a region of interest (yellow dashed circle) is overlaid to demonstrate a spike in local Ca^2+^ activity. Ca^2+^ spike is observed when the intensity normalized to basal intensity is plotted (yellow line, F/F_0_). **(C’)** Time-projection of the time lapse movies. We observed a significant number of Ca^2+^ spikes in the 1 h time interval when GJs were inhibited. Yellow dashed lines indicated disc boundary**. (C”)** A kymograph generated demonstrates instances of Ca^2+^ spike activity.

To elucidate how gap junction proteins alter Ca^2+^ signals, we simulated a scenario where the initiator cells chosen at random had their V_PLC_ values set to the Hopf bifurcation threshold value of 0.774 and standby cells had a V_PLC_ values that were randomly uniformly distributed between 0.1 and 0.5 (Fig 3A). Under these conditions, no Ca^2+^ activity was observed in the presence of normal functioning GJ communication (Fig 3A’). Next, we compared the effect of blocking gap junction communication in silico. To do so, we set the permeability terms for Ca^2+^ (F_c_) and for IP_3_ (F_p_) to zero. Strikingly, we observed Ca^2+^ spikes in simulated wing disc cells in the absence of GJ communication (Fig 3A”). We explored this phenomenon computationally by considering a single stimulated cell connected to neighboring cells by GJ communication by performing bifurcation analysis on our modified model for a single cell. We observed the emergence of a Hopf bifurcation as expected (S3A Fig). Next, the effect of the initial Hopf bifurcation point (HB_1_) on gap junctional (GJ) permeability of IP_3,_ F_p_, was analyzed. Setting F_c_ to zero and progressively increasing F_p_ increased the critical maximum rate of IP_3_ activation threshold $V_{PLC}^{*}$ where HB_1_ occurred (S3B Fig). Similar trends were observed when F_c_ was increased to 0.05. Thus, our model suggests that inhibition of GJ communication lowers the Hopf threshold necessary to generate Ca^2+^ activity in wing disc epithelial cells.

A closer look into the importance of GJ permeability on the formation of Ca^2+^ signals was taken by varying F_c_ and F_p_ in computational simulations. Similar to the sensitivity analysis performed on *k*_*τ*_ and *τ*_*max*_, GJ permeability was varied by fixed percentages (S4C Fig). We discovered that decreased GJ permeability decreased the propagation of the Ca^2+^ signal across the simulated tissue. However, the fixed percentage values ranging from 50% to 150% of the baseline parameter values (Table 1) did not produce notable changes of the ICW Ca^2+^ pattern. To further investigate this, a wider range of fixed percentages were tested between 1% and 1000% of the baseline parameter values (S7A Fig). Starting from a baseline ICW, a 90% decrease in GJ permeability resulted in a transition from ICWs to ICTs, and eventually to single-cell Ca^2+^ spikes, while a 100% increase in GJ permeability increased Ca^2+^ signal propagation and decreased the frequency (S7A Fig). These findings show that GJ permeability alters the cytosolic residence time for critical messengers such as IP_3_ and Ca^2+^ whose cytosolic concentrations affects Ca^2+^ release from ER in both initiator cells and standby cells. This is consistent with bifurcation analysis demonstrating that GJ permeability influences stimulation threshold required for Ca^2+^ oscillations in cells (S3B Fig).

We tested these computational modeling predictions experimentally. To do so, we pharmacologically inhibited the gap junctional protein Inx2 using Carbenoxolone (CBX) and observed the emergence of Ca^2+^ spikes in the absence of FEX in the culture media (Fig 3B-3B” and 3C-3C”, and S13 and S14 Movies). This further demonstrates that gap junction communication regulates Ca^2+^ dynamics in the wing disc pouch.

### GJ permeability modulates Ca^2+^ signaling during development

**Fig 4:**
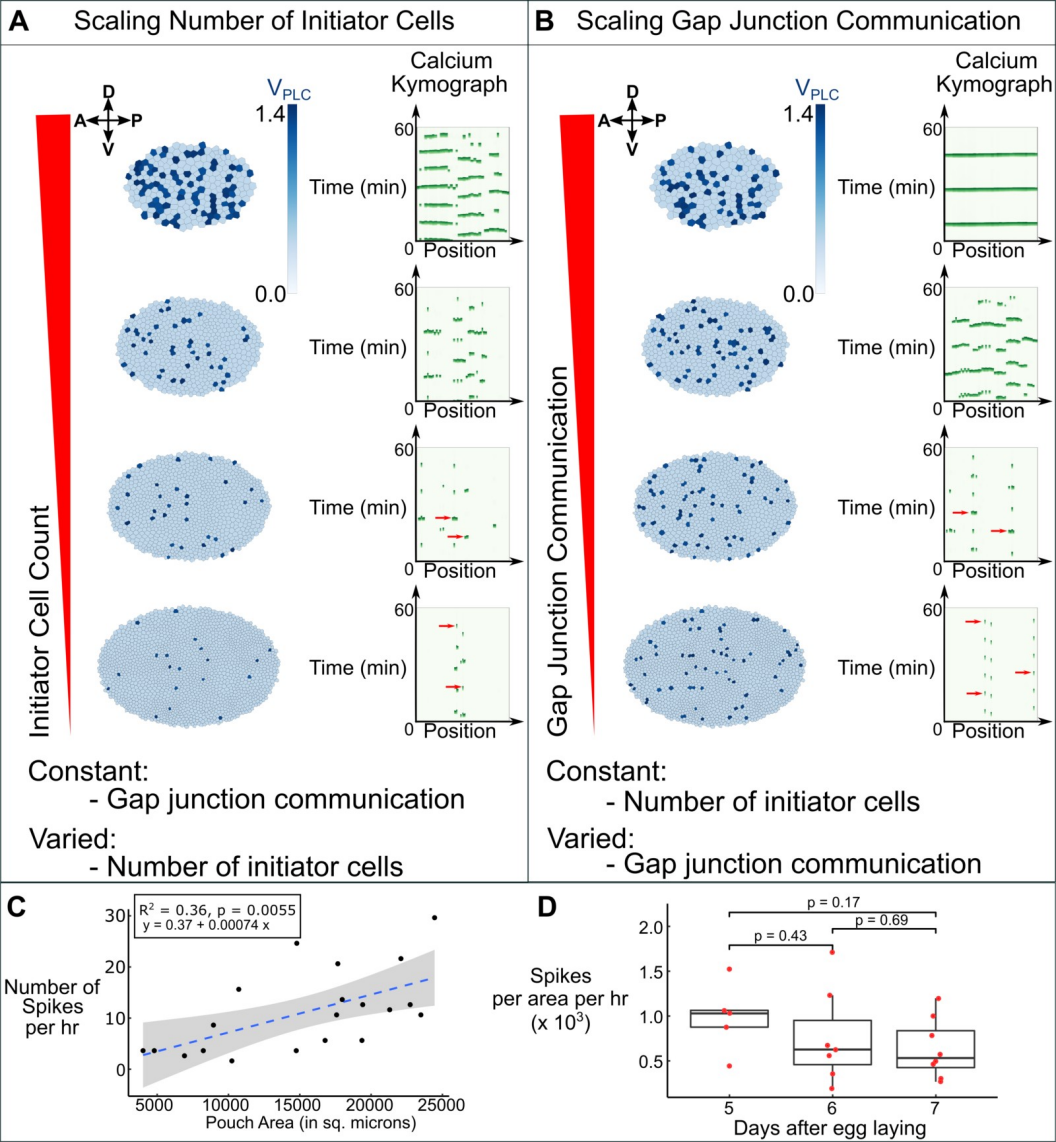
GJ permeability determines total tissue-level signaling activity as development progresses. **(A-B)** Simulations of Ca^2+^ signaling for wing discs of increasing size. **(A)** *(Left column)* The total number of initiator cells was varied with tissue size according to the following equation: $N_{initiators}=8,000∙N_{cells}^{-0.8}$ while GJ permeability was held constant. The V_PLC_ of all standby cells was restricted to values of 0.40, with initiator cells being denoted by dark blue cells with V_PLC_ values between 1.3 and 1.5. *(Right column)* Associated 2D kymographs of the simulated pouches shown in **A**. **(B)** *(Left column)* The GJ permeability is varied while holding the total number of initiator cells constant. GJ permeability was varied according to the following equation: $F_{p}=800∙N_{cells}^{-1.8}$ and *F*_*c*_ = 0.1∙*F*_*p*_. The total number of initiator cells were held constant in this scenario (*N*_*initiator*_ = 65) and standby cells were restricted to V_PLC_ values of 0.40. *(Right column)* Associated kymographs of the simulated pouches shown in **B**. Both scaling models demonstrate high Ca^2+^ signaling activity in small discs and gradually regress to low Ca^2+^ signaling activity in large discs. **(C-D)** Experimental validation of the computational predictions in which the discs were cultured ex vivo in Grace’s low 20E media (basal media) for 1 h in the absence of any stimulus triggering Ca^2+^. **(C)** Quantification of total number of Ca^2+^ spikes in different sized wing disc pouch during 1 h culture. A linear regression line was fit to the data set and the p-value for the slope of the fitted line is shown. Since the *p-*value is less than 0.05 (level of significance), a positive correlation between size and the number of spikes could be inferred. Grey region corresponds to 95% confidence bands of the trend line. **(D)** Quantification of total number of spikes in disc pouch scaled with respect to pouch size during various stages of larval development. *p*-values were obtained by Mann Whitney U test.

Of note, the integrated intensity of Ca^2+^ signaling throughout the wing disc pouch decreases during development suggesting an inverse relationship between Ca^2+^ signaling activity and organ growth rates [19]. Therefore, we investigated the role of tissue size in altering Ca^2+^ signaling dynamics to propose an explanation for this finding. To do so, we simulated Ca^2+^ signaling in different sized wing discs. We hypothesized two different possible scenarios that could explain a decrease in integrated Ca^2+^ signaling activity. In the first scenario, the total number initiator cells was allowed to decrease in a power law fashion as development proceeds while GJ permeability remained constant. This resulted in a decay in total Ca^2+^ signaling activity with a transition from intercellular waves to predominantly single-cell spiking activity (Fig 4A). The fraction of initiator cells was varied according to the following equation:

$$N_{initiators}=8,000∙N_{cells}^{-0.8},$$ (8)

where *N*_*cells*_ is the total number of cells in the simulated tissue. The power-law relationship exponential value of -0.8 was used from the discovery that integrated Ca^2+^ intensity scales in a similar power-law fashion detailed in one of our previous publications [19]. The constant in front of the equation, 8,000, was chosen to output physiologically realistic fractions of initiator cells with the simulated tissue sizes. In the second scenario, GJ permeability was set to decrease with increasing organ size while the total number of initiator cells was held constant at *N*_*initiators*_ = 65 (Fig 4B). GJ permeability was varied according to the following equation:

$$F_{p}=800∙N_{cells}^{-1.8},$$ (9)

where *F*_*c*_ is directly proportional to *F*_*p*_ such that *F*_*c*_ = 0.1∙*F*_*p*_. Similar to the scenario where initiator cell count was scaled, we investigated a scenario of scaling gap junction communication. We selected a power-law relationship value of -1.8 as an analogy to the relationship between integrated Ca^2+^ signaling activity we reported previously [19]. To ensure consistency across the two simulation setups, all other parameter values aside from initiator cell count and GJ permeability are those listed in Table 1 with V_PLC_ values of standby cell equal to 0.40. Simulations corresponding to each scenario show a decrease in progression of Ca^2+^ signaling activity starting from ICWs and intercellular transients in smaller simulated discs and decaying to intermittent single-cell spikes in larger simulated discs (Fig 4A and 4B). This suggests that both scenarios provide a possible explanation for the decrease in integrated Ca^2+^ signaling activity observed in wing discs as development progresses.

To distinguish between these scenarios, we cultured wing discs from multiple developmental stages from days 5–7 after egg laying (AEL) without any agonist stimulation and observed the resulting Ca^2+^ dynamics. We reasoned that the lack of agonist stimulation would reveal cells with phospholipase activity sufficient to create spikes, and this could further be considered initiator cells. We observed single-cell Ca^2+^ spikes that we interpret as characteristic of “initiator” cells in all samples independent of sizes. We did not observe Ca^2+^ transients or waves in unstimulated smaller wing discs obtained day 5 AEL. This is consistent with agonist stimulation increase in PLC activity in standby cells. We next characterized the total number of spikes in the discs of all sizes. We found a positive correlation between the total number of spikes and the size of the disc pouch (Fig 4C). The difference in spiking activity between discs of varying ages was not significant when scaled for pouch size (Fig 4D). This is consistent with a scenario of a relatively constant number of initiator cells in the system with overall GJ permeability decreasing as the organ reaches its terminal size. This scenario is further supported by findings from previous reports showing a decrease in GJ permeability as larval development proceeds [39,40]. Furthermore, a decrease in GJ permeability increases the net cytosolic residence time and effective concentrations of IP_3_ and Ca^2+^ within the cytosol leading to an increased instance of cytosolic Ca^2+^ spikes.

### Gαq overexpression induces intercellular Ca^2+^ waves and reduces wing size

**Fig 5:**
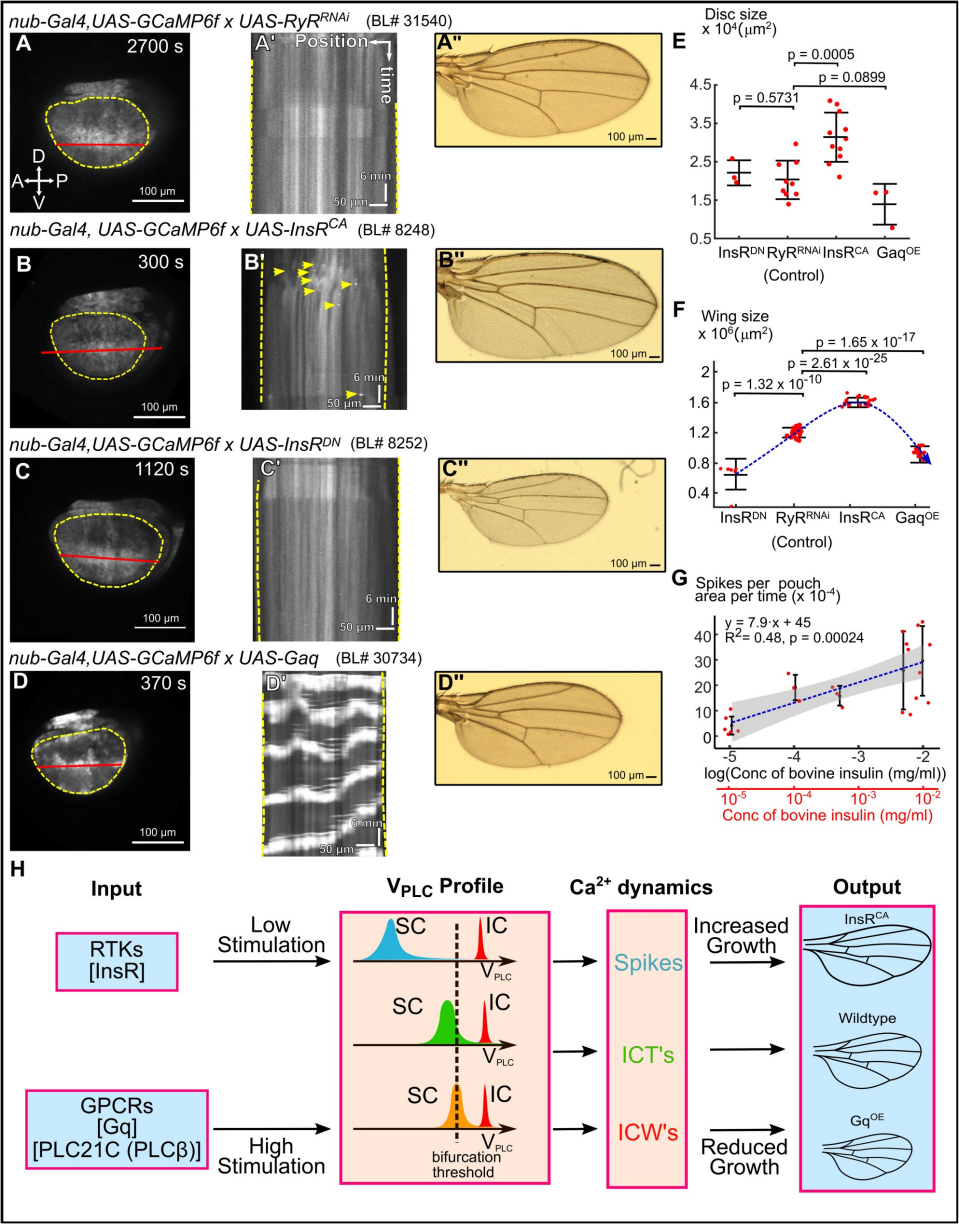
GPCR and insulin signaling regulate wing size and differentially regulate Ca^2+^ signaling. **(A-D)** Montages of time-lapse movies of wing discs cultured ex vivo. **(A’-D’)** Kymographs of the corresponding time-lapse movies. **(A"-D")** Adult wings from the indicated genetic perturbation. **(E)** Quantification of the wing disc sizes for the indicated genetic perturbations. **(F)** Quantification of the adult wing sizes for the indicated perturbations. **(G)** Quantification of Ca^2+^ spikes when insulin dose is progressively increased in ex vivo cultures. A linear regression trend line was fit to the data and the *p*-value of the slope is shown. Since the *p*-value is less than 0.05, a positive correlation between spikes and the log(concentration) can be inferred. Grey region illustrates 95% confidence bands of the linear regression **(H)** Summary of key findings based on the proposed model for tissue-level regulation of dynamics in epithelial tissues. Different cell surface receptor stimulation produces varying V_PLC_ profiles of developing tissues causing Ca^2+^ signaling and varied tissue responses. Scale bars in (A-D) and (A"-D") represent 100 μm, while yellow dotted lines indicate pouch boundaries, and red lines indicate x-y positions in the kymograph. Horizontal scale bars in (A’-D’) represent 50 μm. Vertical scale bars in (A’-D’) represent 6 min. Student t-test was performed. Labels in (E) represent crosses of UAS-transgene to parental nub>GCaMP6f in the case of InsR^CA^ and InsR^DN^ or nub>GCaMP6f; mcherry in the case of RyR^RNAi^ or Gαq. The UAS lines used are UAS-RyR^RNAi^ (BL#31540), UAS InsR^CA^ (BL#8248), UAS-InsR^DN^ (BL#8252) and UAS-Gq (BL#30734) respectively.

Next, using the GAL4/UAS system (S8 Fig), we overexpressed the wild type splice 3 variant of *Drosophila* Gαq in the wing disc to characterize how different classes of upstream signals impact the spatiotemporal patterns of Ca^2+^ signaling [42]. G protein-coupled receptor (GPCR) activation stimulates Gαq-driven PLC*β* activity to generate IP_3_ and Ca^2+^ [43]. Strikingly, ectopic Gαq expression was sufficient to generate robust formation of intercellular waves independent of the presence of FEX in the media (Fig 5D and 5D’). The waves were periodic in nature and were similar to FEX-induced waves (Fig 1C). This experimental finding most likely resembles our previous simulation of ICWs with a small fraction of randomly located initiator cells surrounded by standby cells (Fig 2C). Additionally, the wing disc size (day 6 AEL) and adult wing size were significantly reduced when Gαq was overexpressed (Fig 5E and 5F). To understand whether the reduction in the wing size was due to changes in proliferation or cell growth, we quantified the total number of cells in the region bounded by the LIII, LIV, ACV wing veins and the wing margin. We observed a reduction in the total number of trichomes, where each individual trichome corresponds to a cell [44]. Furthermore, we found that cell size was reduced when Gαq was overexpressed (S9F Fig). However, the wing shape is not significantly affected when Gαq is overexpressed (S9G Fig). In sum, increasing the concentration of Gαq in the pouch is sufficient to generate periodic Ca^2+^ waves. Further, these periodic Ca^2+^ waves are correlated with reduction in wing and disc size suggesting that tissue-wide Ca^2+^ wave activity may play a role in determining final organ size via growth inhibition.

### Insulin signaling increases wing size but only generates localized Ca^2+^ signals

Because FEX is an undefined cocktail of biochemical factors, we tested whether specific ligands added to the organ culture affects Ca^2+^ signaling activity and growth. In addition to FEX, insulin is often added to organ culture media to stimulate cell proliferation [45,46]. Hence, we tested whether insulin signaling regulates Ca^2+^ signaling activity independent of FEX. Similar to other experiments, we upregulated and downregulated insulin signaling in the wing disc using the GAL4/UAS expression system (S8 Fig). As expected, wing disc size and adult wing size were decreased when insulin signaling is downregulated (Fig 5C”, 5E, and 5F). Strikingly, we observed that activation of insulin stimulated pathways results in localized Ca^2+^ spikes and ICTs (Fig 5B and 5B’). Titrated concentration of insulin in the culture media demonstrated that a higher concentration of insulin increased the number of spikes (Fig 5G). Quantification of the Ca^2+^ spikes showed a positive correlation between spikes normalized to area of the pouch with log of insulin concentration (Fig 5G). However, even high concentrations of insulin were not sufficient to generate periodic ICWs. In contrast, expressing a dominant negative form of the insulin receptor resulted in minimal Ca^2+^ spiking activity (Fig 5C and 5C’) [47]. These results demonstrate that insulin signaling stimulates Ca^2+^ activity in the wing disc. Overall, these results indicate that the agonist class encodes different spatiotemporal dynamics of Ca^2+^ signaling at the tissue scale.

In silico simulations with parameter variations in half activation of the PLC parameter, K_PLC_, and GJ permeability were performed to test scenarios of how insulin signaling can only generate Ca^2+^ spiking activity even under saturating conditions (S7B and S10 Figs). These two parameters were chosen because K_PLC_ can potentially be influenced by downstream of insulin receptor activity, a receptor tyrosine kinase (Fig 1D) or by the biochemical activity of phospholipase C γ. Additionally, single-cell spikes were only observed when gap junction communication was inhibited either experimentally (Fig 3B and 3C) or in silico (Figs 3A and S7A). Although large K_PLC_ variations influenced signal frequency (S7B Fig), there was no instance in which varying K_PLC_ resulted in exclusive production of Ca^2+^ spikes. To further investigate this, both GJ permeability and K_PLC_ were varied simultaneously (S10 Fig). From a baseline intercellular wave (S10A Fig), K_PLC_ was increased from its baseline value while GJ permeability was simultaneously decreased from its baseline value. Only when GJ permeability was decreased was there observance of single-cell Ca^2+^ spikes (S10 Fig). Thus, the computational model suggests that insulin signaling must not only stimulate IP_3_ production, but also inhibit GJ permeability to account for the limitations of spatial spread of Ca^2+^ signaling.

As a first step to assess whether Ca^2+^ levels in the cell directly control organ sizes, we exploited the Ca^2+^ buffering effects induced by high levels of GCaMP6f sensor expression. The GCaMP6f sensor consists of a M13 fragment of myosin light chain kinase, GFP and Calmodulin (CaM) to which Ca^2+^ binds [48]. To elucidate the role of Ca^2+^ signaling in insulin mediated growth, we compared the effects of co-expressing the GCaMP6f sensor (*K*_*d*_ = 375±14 *nM*) to decrease the amount of free cytosolic Ca^2+^ [49]. We found that co-expressing GCaMP6f sensor increased wing size when insulin signaling was also stimulated (S11 Fig). This increase in size was also observed in control wing disc without insulin upregulation (S11A and S11B Fig). This analysis provides additional evidence that buffering cytosolic Ca^2+^ signaling influences the final tissue size. In contrast, when we expressed Gαq and GCaMP6f, we did not observe a significant decrease in size when compared to just expressed Gαq without GCaMP6f (S11C and S11D Fig). This may be due to the inability of GCaMP6f expression to buffer the high levels of Ca^2+^ in the cytosol when Gαq is overexpressed. We also observed a severe reduction in the wing size, along with vein defects, in flies that were homozygous for the GCaMP6f sensor, consistent with a buffering role for high concentrations of GCaMP6f expression (S12 Fig). To validate this finding, we compared the adult wing sizes from flies expressed one and two copies of GCaMP6f. Strikingly, increasing the dose of the relative GCaM6f expression decreased the size (S12C and S12D Fig). We observed a severe reduction in size as expression of the transgene was further increased with the presence of two copies of the GAL4 driver. To further elucidate the sponging effects of Ca^2+^, we expressed CaMKII, which binds to Ca^2+^/CaM complex [50]. Similar to overexpressing GCaM6f, we observe a significant increase in the wing area (S12E and S12F Fig). Collectively, these results support a role of cytosolic Ca^2+^ concentration as either a growth enhancer or suppressor and that an optimal amount of Ca^2+^ signaling is required for robust size control.

## Discussion

The main finding of this work is the discovery of a parsimonious mechanistic model that links global hormonal stimulation of Ca^2+^ signaling to emergent spatiotemporal classes of signaling dynamics. Further, we identify downstream correlations to the final organ size that suggest these signaling dynamics may mediate growth-related information used by the system to tune organ size. To do so, we developed a geometrically accurate computational model of Ca^2+^ signaling in the *Drosophila* wing imaginal disc, a premier model for studying conserved cell signaling mechanisms [23,51,52]. Previously, we discovered a correlation between Ca^2+^ signaling during larval growth and final organ size. Four distinct patterns of Ca^2+^ signaling activity occur in the wing imaginal disc pouch as observed in vivo and ex vivo experiments [19]. Through systems-level computational analysis, we established the essential conditions required for generating the different patterns.

The model predicts two cell types with different levels of IP_3_ production: “initiator cells” with high IP_3_ production and “standby cells” with baseline IP_3_ production levels. The presence of initiator cells is necessary, but not sufficient, to induce multicellular Ca^2+^ signaling. Additionally, the distribution of the maximal rate of IP_3_ production, V_PLC_, in the standby cells determines the spatial range of Ca^2+^ signaling. As V_PLC_ values of standby cells approaches the Hopf bifurcation threshold, Ca^2+^ activity transitions from single-cell signals toward global signals.

What are the possible functional implications of a tissue consisting of initiator and standby cells? A recent study on electrical signal transmission in bacterial communities suggests that the transition from localized short-range signaling to global community-level communication is associated with a cost-benefit balance [53]. In that context, long-range signaling increases the overall fitness of the community against chemical attacks, while a reduction in growth rate is the cost to individual cells. In the wing disc, a similar analogy can be drawn where significant generation of long-range Ca^2+^ signals due to overexpression of Gαq results in reduced wing disc growth. Thus, the proposed model in this work can also be characterized as a cost-benefit tradeoff within the context of tissue-level signaling. For instance, it has been suggested that the fast Ca^2+^ waves facilitate migration and proliferation of the healing cells by inhibiting excessive apoptotic response during wound healing in epithelia [54].

Our model also predicts that the inhibition of GJs lowers the Hopf threshold necessary for generating Ca^2+^ spikes. We have validated this prediction experimentally where inhibition of GJs results in the formation of Ca^2+^ spikes in the absence of external agonist. Further, computational simulations demonstrate that as GJ permeability decreases, there is a transition of activity from synchronous global to asynchronous local Ca^2+^ activity. How gap junctional mediated Ca^2+^ signaling is connected to the regulation of cell mechanics is not currently understood and warrants further investigation. One possibility is that tension impacts the level of gap junction communication between cell and may also influence the activity of mechanosensitive ion channels [55]. Feedback between Ca^2+^ signaling and cell mechanics may play important roles in ensuring tissue growth and morphogenesis. This is evident from our previous experimental work and other experimental studies in the literature where knockdown of gap junctional proteins such as Inx2 leads to a reduction in wing and eye size in *Drosophila* [19,56].

Upregulated insulin signaling increases the formation of Ca^2+^ spikes. One possible implication is that insulin signaling inhibits GJs in addition to increasing PLC*γ* activity. This implication is consistent with the role of insulin signaling in inhibiting gap junction proteins Connexin43 in vertebrates [57–59]. Thus, the increase of adult wing and developing wing disc size from higher insulin activity correlates with higher levels of localized Ca^2+^ spiking activity, which has a limited total integrated calcium signal at that tissue level. In contrast, Ca^2+^ waves induced by Gαq overexpression are correlated with smaller adult wings and wing discs. Higher Gαq expression, which activates PLC*β* activity results in robust production of tissue-scale intercellular Ca^2+^ waves. We show experimentally that insulin signaling controls Ca^2+^ spike activity in the wing disc, potentially through GJ inhibition or PLC*γ* activation, whereas GPCR-based Gαq signaling is sufficient to generate global Ca^2+^ waves. These results are consistent with previous reports of Ca^2+^ spikes being observed in discs that have reached their final size, and Ca^2+^ waves being observed in smaller developing discs [19]. A recent study found that Ca^2+^ signals are initiated in response to wounding by the G-protein coupled receptor Methuselah like 10 (Mthl10) [60]. Mthl10 is activated by Growth-blocking peptides (Gbps) [60]. Whether Mthl10 also is involved in developmental growth requires further investigation, but may be consistent with our findings that intercellular calcium wave activity inhibits organ growth. These findings suggest that Ca^2+^ acts as both a growth enhancing and growth inhibiting signal dependent upon the tissue-level scale of the activity and level of gap junction coupling.

The spatial range of tissue-level signaling is determined by how the IP_3_ production is organized with respect to a Hopf bifurcation threshold throughout the tissue. Localized transients are correlated with larger wings induced by insulin-stimulated growth whereas global signaling is correlated with smaller wings that are stimulated by upstream GPCRs and Gαq upregulation (Fig 5H). This resembles a paradoxical network motif [61] where Ca^2+^ signaling has two opposite effects on the same downstream target, dependent upon the tissue-level magnitude of the Ca^2+^ signaling. Within the context of the hypothesized IP_3_/shunt model proposed in our previous study, the strong induction of Ca^2+^ waves will reduce the level of phosphatidylinositol 4,5-bisphosphate (PIP_2_), a key substrate for growth [19]. This may occur as high levels of Gαq/PLC activity are proposed to deplete PIP_2_ levels [62]. This would occur due to substrate depletion of PIP_2_ through promotion of IP_3_ generation and downstream activity by stimulation of PLC activity. In turn, this would lead to reduced availability of PIP_2_ for conversion of PIP_2_ to phosphatidylinositol-trisphosphate (PIP_3_), a key second messenger for stimulating protein kinase AKT and downstream growth promotion [63].

Similar to the reduced wing size observed in this study due to overexpression of Gαq, we have also reported that knockdown of Gαq gene decreases wing size in our previous study (S2 Table) [19]. Comparing the reduction in wing size due to perturbation of Ca^2+^ signaling components with known size control genes such as morphogens (Dpp, Wg, Hippo) and mechanical transducers (RoK) indicate that the reduction in size is comparable to when Ca^2+^ signaling is perturbed (S2 Table). Taken together, these experimental findings imply that Gαq signaling is paradoxical in nature. In the context of conserved network motif observed in biological systems, paradoxical components have the ability to activate and inhibit the downstream target despite a single source of stimulus [61]. Gαq could possibly be a growth promoter and growth inhibitor. This correlation motivates further studies that map out the exact molecular players that are downstream of Gαq signaling. This will require careful quantification of PIP_2_ and PIP_3_ under genetic perturbations of GPCR signaling. Additionally, future work is needed to quantify the metabolic benefits and costs of Ca^2+^ signaling during tissue growth to observe if abundant use of metabolic resources to consistently propagate global activity is explanatory for the reduction in size in Gαq overexpression wings.

## Materials and methods

### Drosophila genetics

We used the GAL4/UAS system to express modulators of the Ca^2+^ signaling pathway in the wing disc [64,65]. A nub-GAL4, UAS-GCaMP6f reporter tester line was created by recombining nub-GAL4 and UAS-GCaMP6f lines [66]. Additionally, a second tester line was used that also includes UAS-mcherry. Gene perturbations were generated by crossing the tester line to either RNAi-based transgenic lines (UAS-Gene X^RNAi^) or gene overexpression (UAS-Gene X). The following UAS transgenic lines were used: UAS-RyR^RNAi^ (BL#31540) [67], UAS-Gq (BL#30734) [67], UAS-InsR^CA^ (BL#8248) [68], UAS-InsR^DN^ (BL#8252) [69]. Progeny wing phenotypes are from F1 male progeny emerging form the nub-Gal4, UAS-GCaMP6f/CyO x UAS-X cross or nub-Gal4, UAS-GCaMP6f/CyO; UAS-mcherry x UAS-X cross. Flies were raised at 25°C and on a 12-hour light cycle.

### Live imaging

Wandering third instar larva approximately 6 days after egg laying were dissected in ZB media with 15% fly extract to obtain wing discs [19]. ZB media + 15% fly extract contains 79.4% (v/v) ZB media, 0.6% (v/v) of 1 mg/ml of insulin (Sigma aldrich), 15% ZB-based fly extract and 5% penicillin/streptomycin (Gibco). Wing discs were loaded into the previously described REM-Chip [70] and imaged using Nikon Eclipse Ti confocal microscope with a Yokogawa spinning disc and MicroPoint laser ablation system. Image data were collected on an IXonEM+colled CCD camera (Andor technology, South Windsor, CT) using MetaMorph v7.7.9 software (Molecular devices, Sunnyvale, CA). Discs were imaged at three z-planes with a step size of 10 μm, 20x magnification and 10-seconds intervals for a total period of one hour, with 200 ms exposure time, and 50 nW, 488 nm laser exposure at 44% laser intensity. We blocked GJ by inhibiting innexin-2 using Carbenoxolone (Cbx, Sigma Aldrich) drug [66]. Wing discs were incubated in ZB + 15% FEX with 30 μM Cbx for one hour before imaging. To induce Ca^2+^ transients, we imaged wing discs in ZB media + 2.5% FEX [71]. Ca^2+^ waves were induced by imaging the wing disc in ZB media + 15% FEX. Ca^2+^ fluttering was observed when discs were imaged in ZB media + 40% FEX respectively. For experiments reported in Figs 3, 4, and 5, wing imaginal discs were cultured in Grace’s media with low 20E (Dye et al., 2017). Briefly, basal Grace’s media was prepared by addition of Grace’s medium (Sigma, G9771) with 5 mM BisTris, 5% fetal bovine serium (FBS; ThermoFisher/Invitrogen, 10370098) and Pennicillin-Streptomyocin (Sigma P4333, 100x stock solution) along with 20 nM 20E (Sigma, H5142). For the Cbx experiment reported in Fig 3, we cultured wing discs in Grace’s cocktail with 30 uM Cbx (Sigma Aldrich). For insulin dose response experiments reported in Fig 5, we added appropriate volume of Bovine insulin (Sigma, I5500) to the Grace’s cocktail respectively.

### Image processing

All the images were processing in FIJI. Volume viewer plugin was used to generate 3D Kymographs. Briefly, TIFF stacks had background subtracted using the rolling ball background subtraction algorithm with a rolling ball radius of 15. The TIFF stack was then processed by the volume viewer plugin. Stacks were adjusted using the base Fire LUT setting to portray the signaling intensities. A similar approach was followed for the simulation outputs.

### Model formulation

Fig 1 summarizes the experimental system and data. Different classes of patterns emerge at the tissue-level as the level of global stimulation increases: spikes, intercellular Ca^2+^ transients (ICTs), intercellular Ca^2+^ waves (ICWs) and global fluttering [19]. However, a mechanistic understanding linking hormonal stimulation levels to transitions in these qualitative classes of organ-level signaling is lacking. We therefore formulated a computational model to test mechanistic hypotheses that could explain the observed Ca^2+^ signaling dynamics.

### Intracellular model

A modified model of Ca^2+^ signaling toolkit is based on adaptation of previous single-cell model of calcium signaling [30]. The model is summarized in the computational model section of the main text. To recapitulate the same time resolution as the experiments, the simulation time is 1 hour and for generating movies, samples are obtained every 10 s.

### Tissue model

For constructing a realistic model of the tissue, we used experimental images of a wing pouch to build an accurate model of the tissue structure. S1 Fig depicts the structure of the tissue used for simulations and the statistics of the corresponding network. A pouch was constructed computationally using EpiTools. We first segmented the apical cell boundaries using images from Ecad::GFP line. Then, the centroids of segmented cells were used to define cellular positions in the simulated wing disc. A Voronoi tessellation followed by multiple rounds of Lloyd’s relaxation was used to define a template wing disc that matches the experimentally observed network topology.

### Quantification and statistical analysis

#### Quantification of adult wings and statistics

Total wing area was measured using ImageJ. We traced the wing margin by following veins L1 and L5 and the wing hinge region was excluded from the size analysis. All statistical analyses were performed using MATLAB or R. For comparisons, we used student t-tests to assess the statistical significance. *p*-value, standard deviation and sample size (*n*) are as indicated in each figure and legend.

## Supporting information

S1 Fig Computational framework. **(A)** Experimental *Drosophila* imaginal disc showing cell boundaries marked with Ecad∷GFP. The developing wing pouch has been segmented using ImageJ. The genotype of the *Drosophila* used is *yw;;dECad*::*GFP* (BL# 46556) **(B)** A pouch constructed computationally using EpiTools that served as a basis for Ca^2+^ signaling simulations. In brief, cells were segmented from a wing disc. Centroids of segmented cells were used to define cellular positions in the simulated wing disc. A Voronoi tessellation followed by multiple rounds of Lloyd’s relaxation [72] was used to define a template wing disc that matches the experimentally observed network topology.
(TIF)

S2 Fig Single-cell Ca^2+^ dynamics. The model was calibrated to match experimental single-cell frequency and amplitude. Perturbations to stimulation strength V_PLC_ **(A)** alters the frequency and amplitude of Ca^2+^ oscillations whereas perturbations to *k*_*τ*_ **(B)** only alters the frequency.
(TIF)

S3 Fig Bifurcation analysis of the modified model. **(A)** Bifurcation diagram for the modified model used in this study; shown are the maxima and minima of the Ca^2+^ oscillations (dots) and the Ca^2+^ steady states (solid and dashed lines) as a function of the stimulus (V_PLC_). Solid and dashed lines in red indicate stable and unstable states, respectively. Red dots indicate the maxima and minima of unstable limit cycle and the black dots indicate maxima and the minima of the stable limit cycle. HB, Hopf bifurcation occurs when V_PLC_ is varied. Inset figure shows the period of Ca^2+^ oscillations as a function of V_PLC_. **(B)** Blocking permeability of IP_3,_ F_p_ via gap junctions decreases V_PLC_ where the initial Hopf bifurcation point (HB_1_) occurs in the bifurcation diagram. Block dots indicate conditions where permeability of Ca^2+^, F_c_ is set to 0. Red diamonds indicate conditions where F_c_ is set to 0.5.
(TIF)

S4 Fig Model sensitivity analysis to parameters: *k*_*τ*_, *τ*_*max*_, *F*_*p*_, *F*_*c*_, *K*_*r*_ and *K*_*p*_. Five different parameters in the 2D model were varied from their baseline values (BV). V_PLC_ profiles of simulated tissues were selected to generate intercellular waves (red box) and are identical across all simulations to enable comparisons. Each row represents one parameter being varied in a scaling manner by fixed percentages listed in each column (*i*.*e*., 50% of a BV of 1.5 μM would simulate a value of 0.75 μM). Simulations were performed varying only one parameter while holding all others constant at their BVs. Signal frequency is observed through number of bands in the kymograph, and signal duration is observed through thickness of the bands in the kymograph. **(A)** Decreased *k*_*τ*_ (BV of 1.5 μM) increased frequency and decreased duration of the Ca^2+^ signal whereas increased *k*_*τ*_ did not influence the signal. **(B)** Decreased *τ*_*max*_ (BV of 800 s^-1^) increased frequency and decreased duration of the Ca^2+^ signal whereas increased *τ*_*max*_ decreased frequency and increased duration. **(C)** Decreased gap junction (GJ) communication *F*_*p*/*c*_ (BVs of 0.005 μM^2^ s^-1^ for *F*_*p*_; 0.0005 μM^2^ s^-1^ for *F*_*c*_) decreased propagation of the Ca^2+^ signal whereas increased GJ communication increased propagation. Signal propagation is visualized by the uniformity of the signal across the tissue. **(D)** Decreased *K*_*r*_ (BV of 0.4 μM) decreased frequency and duration of the Ca^2+^ signal whereas increased *K*_*r*_ decreased frequency but increased duration. **(E)** Decreased *K*_*p*_ (BV of 0.13 μM) increased frequency of the Ca^2+^ signal whereas increased *K*_*p*_ decreased frequency.
(TIF)

S5 Fig Model sensitivity analysis to parameters: *k*_5,*P*_, *K*_*a*_, *K*_*PLC*_, *V*_*SERCA*_ and *β*. Five different parameters in the 2D model were varied from their baseline values (BV). V_PLC_ profiles of simulated tissues were selected to generate intercellular waves (red box) and are identical across all simulations to enable comparisons. Each row represents one parameter being varied in a scaling manner by fixed percentages listed in each column (*i*.*e*., 50% of a BV of 0.66 s^-1^ would simulate a value of 0.33 s^-1^). Simulations were performed varying only one parameter while holding all others constant at their BVs. Signal frequency is observed through number of bands in the kymograph, and signal duration is observed through thickness of the bands in the kymograph. **(A)** Decreased *k*_5,*P*_ (BV of 0.66 s^-1^) increased frequency of the Ca^2+^ signal whereas increased *k*_5,*P*_ decreased frequency. **(B)** Decreased *K*_*a*_ (BV of 0.08 μM) increased frequency of the Ca^2+^ signal to the point of observing constant activity, whereas increased *K*_*a*_ decreased frequency to the point of loss of signal in a 60 minute simulation. **(C)** Decreased *K*_*PLC*_ (BV of 0.2 μM) increased frequency of the Ca^2+^ signal whereas increased *K*_*PLC*_ decreased frequency to the point of loss of signal in a 60 minute simulation. **(D)** Decreased *V*_*SERCA*_ (BV of 0.9 μM s^-1^) increased frequency and duration of the Ca^2+^ signal to the point of observing constant activity, whereas increased *V*_*SERCA*_ decreased frequency to the point of loss of signal in a 60 minute simulation. **(E)** Decreased *β* (BV of 0.185) increased frequency and duration of the Ca^2+^ signal to the point of observing constant activity, whereas increased *β* decreased frequency to the point of loss of signal in a 60 minute simulation.
(TIF)

S6 Fig Model sensitivity analysis to parameters: *K*_*SERCA*_, *c*_*tot*_, *k*_1_ and *k*_2_. Four different parameters in the 2D model were varied from their baseline values (BV). V_PLC_ profiles of simulated tissues were selected to generate intercellular waves (red box) and are identical across all simulations to enable comparisons. Each row represents one parameter being varied in a scaling manner by fixed percentages listed in each column (*i*.*e*., 50% of a BV of 0.1 μM would simulate a value of 0.05 μM). Simulations were performed varying only one parameter while holding all others constant at their BVs. Signal frequency is observed through number of bands in the kymograph, and signal duration is observed through thickness of the bands in the kymograph. **(A)** Decreased *K*_*SERCA*_ (BV of 0.1 μM) decreased frequency of the Ca^2+^ signal whereas increased *K*_*SERCA*_ increased frequency. **(B)** Decreased *c*_*tot*_ (BV of 2 μM) decreased frequency of the Ca^2+^ signal whereas increased *c*_*tot*_ increased frequency. **(C)** Decreased *k*_1_ (BV of 1.11 s^-1^) decreased frequency and duration of the Ca^2+^ signal whereas increased *k*_1_ increased frequency and increased. **(D)** Decreased *k*_2_ (BV of 0.0203 s^-1^) decreased frequency of the Ca^2+^ signal whereas increased *k*_2_ increased frequency.
(TIF)

S7 Fig Model sensitivity analysis to extremes of the parameters: *F*_*p*_, *F*_*c*_ and *K*_*PLC*_. Two different parameters in the 2D model were varied from their baseline values (BV). V_PLC_ profiles of simulated tissues were selected to generate intercellular waves (red box) and are identical across all simulations to enable comparisons. Each row represents one parameter being varied in a scaling manner by fixed percentages listed in each column (*i*.*e*., 50% of a BV of 0.1 μM would simulate a value of 0.05 μM). Simulations were performed varying only one parameter while holding all others constant at their BVs. Signal frequency is observed through number of bands in the kymograph, and signal duration is observed through thickness of the bands in the kymograph. **(A)** GJ permeability of IP_3_ and Ca^2+^ influences synchronization of Ca^2+^ signaling among cells. Decreased gap junction (GJ) communication *F*_*p*/*c*_ (BVs of 0.005 μM^2^ s^-1^ for *F*_*p*_; 0.0005 μM^2^ s^-1^ for *F*_*c*_) results in a transition of intercellular waves to intercellular transients, and to single-cell spikes. Increased GJ communication increased Ca^2+^ signal propagation and decreased signal frequency. Signal propagation is visualized by the uniformity of the signal across the tissue. **(B)** Variations in the half-activation of V_PLC_ term, *K*_*PLC*_ (BV of 0.2 μM), only changed the frequency of the ICWs.
(TIF)

S8 Fig Schematic of expression pattern using the Gal4/UAS system. **(A)** The GAL4/UAS system was used to express *GCaMP6f* transgene along with other transgenes. **(B)** *nubbin* is expressed in the wing disc pouch and the adult wing phenotype provide a readout of final phenotype after transgene expression in the wing disc pouch during larval stage.
(TIF)

S9 Fig Overexpression of Gαq decreases cell number and cell size. **(A-B)** Wings from adult males expressing *RyR*^*RNAi*^ and wild type Gαq splice 3 variant with *nubbin-Gal4*, *UAS GCaMP6f*, *UAS mcherry*. **(A’-B’)** Region of interest (ROI) where the total number of setae was calculated. **(C-F)** Quantification of the wing size defined here as the area bounded by LIII, LIV, ACV and the wing margin, total cell number and cell area. Overexpression of Gαq in the pouch results in a decrease in total wing area, cell number and cell size. 10 samples were analyzed per condition. Error bars represent standard deviation. **(G)** Quantification of roundness of the adult wing. Gαq overexpression does not affect the roundness. Student t-test was used for statistical significance testing.
(TIF)

S10 Fig GJ inhibition is the key driver of single-cell Ca^2+^ spike activity. To replicate the ex vivo observations of insulin inducing single-cell Ca^2+^ spikes, GJ permeability and the half-activation of V_PLC_ were varied simultaneously in silico. **(A)** A baseline intercellular wave was used as the comparison for how parameter variations changed signal with the following parameter values: IP_3_ gap junction permeability (F_p_) of 0.005 μM^2^s^-1^, Ca^2+^ gap junction permeability (F_c_) of 0.0005 μM^2^s^-1^, and K_PLC_ of 0.20 μM. **(B-E)** *K*_*PLC*_ is increased left-to-right (red bar), and gap junction communication is decreased left-to-right (blue bar). An increase in *K*_*PLC*_ results in a decrease in frequency, while decrease in gap junction communication results in single-cell Ca^2+^ spikes (red arrows).
(TIF)

S11 Fig Sponging cytosolic Ca^2+^ increases overall wing size. **(A-F)** Wings from adult males with the indicated crosses. **(A)** *nubbin-GAL4* x *UAS-RyR*^*RNAi*^ (i.e., nub4>RyR^RNAi^), **(B)** *nubbin-GAL4*, *UAS-GCaMP6f* x *UAS-RyR*^*RNAi*^ (i.e., nub4>GCaMP6f, RyR^RNAi^), **(C)** *nubbin-GAL4* x *UAS-Gaq*^*OE*^ embryonic splice 3 variant of Gaq (i.e., nub4>Gaq^OE^), **(D)** *nubbin-GAL4*, *UAS-GCaMP6f x UAS-Gaq*^*OE*^ (i.e., nub4>GCaMP6f, Gaq^OE^), **(E)** *nubbin-GAL4 x UAS-InsR*^*CA*^ (i.e., nub4>InsR^CA^) gain of function mutant where the α subunit is partially deleted. **(F)** *nubbin-GAL4*, *UAS-GCaMP6f x InsR*^*CA*^ (i.e., nub4>GCaMP6f, InsR^CA^). **(G)** Quantification of adult wings. The genetic encoded calcium sensor, GCaMP6f, binds to Ca^2+^ with high affinity, thus expression of the sensor will to some degree act as a sponge of cytosolic Ca^2+^. Interestingly, the presence of the GCaMP6f sponge with constitutively activated insulin signaling increases the adult wing size (E, F). Similar enhancement of wing size was observed in control wings when GCaMP6f sensor was expressed (A, B). No significant change in the adult wing size was observed when Gαq was overexpressed, suggesting sponging effects are trivialized under Gαq overexpression (C,D). Unpaired student t-test was used, and the p-values are indicated above.
(TIF)

S12 Fig Increasing gene dosage of GCaMP6f Ca^2+^ sensor dramatically reduces wing size. **(A-E)** Wings from adult males of indicated genotypes **(A)** nubG4, UAS RyR^RNAi^, **(B)** nubG4, UAS GCaMP6f, **(C)** nubG4, UAS GCaMP6f/UAS GCaMP6f, **(D)** nubG4, UAS GCaMP6f (Homozygous) **(E)** nubG4, UAS CaMKII **(F)** Quantification of adult wing sizes. As the gene dose of GCaMPf is increased in the wing disc, the overall wing area decreases in size (B, C, D). Overexpressing possible Ca^2+^ downstream target CaMKII increases the wing size consistent with B in which one copy of GCaMP6f was expressed.
(TIF)

S13 Fig Repeated simulations result in the same conclusions. Simulations corresponding to the main text figure conclusions (*i*.*e*., Figs 2–4) were repeated five separate times with five different random number generator seeds. The random number generator seed value determines which cells in a simulated tissue are determined to be initiator cells. For the case of Fig 2, the V_PLC_ values of standby cells also rely on the random number generator seed as the values are sampled randomly from a uniform distribution with set boundaries. The simulations’ resulting video and image outputs were randomized and their inputs were hidden to allow classification of the Ca^2+^ activity. **(A)** Using a graphical user interface (GUI) in MATLAB, the randomized video and kymograph simulations were drawn to show the output kymograph and play the video simulation. A user was tasked to classify the activity of the simulation output as having no activity, single-cell spikes, intercellular transient activity (ICT), intercellular wave activity (ICW), or global fluttering activity. For each main text figure, the five separate simulations had their Ca^2+^ activity classified in four independent runs. Each run corresponds to a brand new running of the classification GUI, each with a different randomization scheme to display the outputs of the simulations. **(B)** The proportions of Ca^2+^ activity are plotted for Fig 2‘s repeated simulations. Runs 1, 2, and 4 all had the same proportions, indicating reproducibility of the simulations’ outputs. Run 3 had mismatched classifications between the ICT and spike class (red arrow), however, the difference in proportions was not significant using a proportions test without a continuity correction [73–75]. **(C)** The proportions of Ca^2+^ activity are plotted for Fig 3‘s repeated simulations. Because Fig 3 was designed to demonstrate either no activity in the case of enabled gap junction communication, or spiking activity in the case of disabled gap junction communication, only two classes of activity appear. In each classification run, there were no differences in the user-recorded classifications. **(D)** The proportions of Ca^2+^ activity are plotted for Fig 4‘s repeated simulations. Runs 1, 2, and 4 all had the same proportions, indicating reproducibility of the simulations’ outputs. Run 3 had mismatched classifications between the ICT and spike class (red arrow), however, the difference in proportions was not significant using a proportions test without a continuity correction.
(TIF)

S1 Table Extended data movies. (DOCX)

S2 Table Changes in wing area for known perturbations through Gal4/UAS system. Note that maximal deviations wing size for strong growth perturbations is in range of 20–50%. References for this table include this study, [19], [76], [77].
(DOCX)

S1 Movie nub-Gal4>UAS-GCaMP6f, UAS-mcherry, ex vivo, spike. (AVI)

S2 Movie nub-Gal4>UAS-GCaMP6f, UAS-mcherry, ex vivo, ICT. (AVI)

S3 Movie nub-Gal4>UAS-GCaMP6f, ex vivo, ICW. (MP4)

S4 Movie nub-Gal4>UAS-GCaMP6f, ex vivo, fluttering. (MP4)

S5 Movie nub-Gal4>UAS-GCaMP6f, in vivo, spikes. (AVI)

S6 Movie nub-Gal4>UAS-GCaMP6f, in vivo, ICT. (MP4)

S7 Movie nub-Gal4>UAS-GCaMP6f, in vivo, ICW. (AVI)

S8 Movie nub-Gal4>UAS-GCaMP6f, in vivo, fluttering. (AVI)

S9 Movie Spike, Simulation output. (MP4)

S10 Movie ICT, Simulation output. (MP4)

S11 Movie ICW, Simulation output. (MP4)

S12 Movie Fluttering, Simulation output. (MP4)

S13 Movie nub-Gal4>UAS-GCaMP6f, ex vivo in Grace’s low 20E media, gap junctions not blocked (Control). (AVI)

S14 Movie nub-Gal4>UAS-GCaMP6f, ex vivo in Grace’s low 20E media with Carbenoxolone, gap junctions blocked. (AVI)

S15 Movie nub-Gal4>UAS-GCaMP6f, UAS-RyRRNAi, ex vivo in Grace’s low 20E media (Control). (AVI)

S16 Movie nub-Gal4>UAS-GCaMP6f, UAS-InsRCA, ex vivo in Grace’s low 20E media. (AVI)

S17 Movie nub-Gal4>UAS-GCaMP6f, UAS-InsRDN, ex vivo in Grace’s low 20E media. (AVI)

S18 Movie nub-Gal4>UAS-GCaMP6f, UAS-GaqOE, ex vivo in Grace’s low 20E media. (AVI)
