# Supplementary figures and images for: From spikes to intercellular waves: Tuning intercellular calcium signaling dynamics modulates organ size control
Source: PLoS Comput Biol. 2021 Nov 1;17(11):e1009543. doi: 10.1371/journal.pcbi.1009543 (PMC8601605; doi:10.1371/journal.pcbi.1009543)

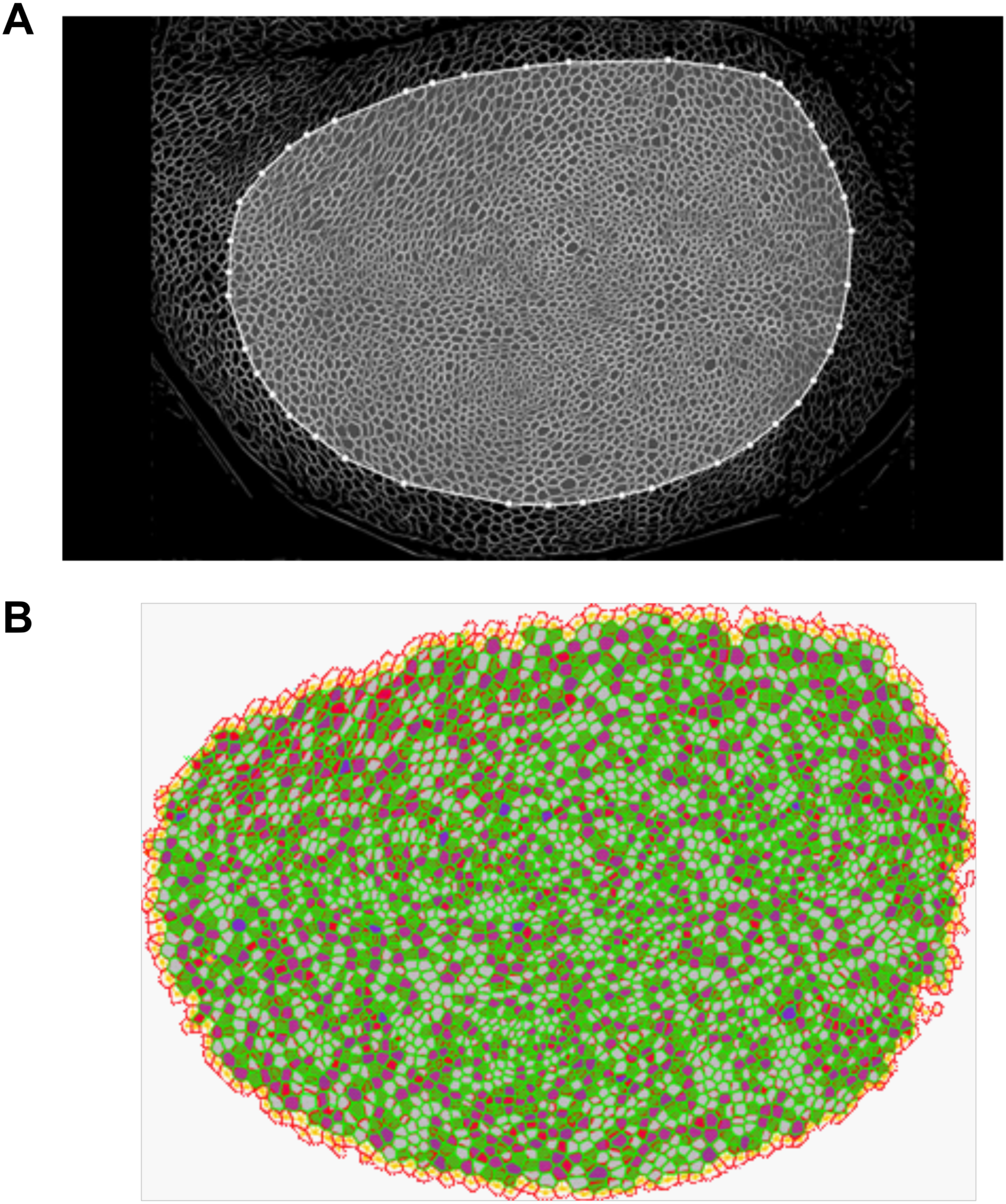

Supplement: S1 Fig — (A) Experimental Drosophila imaginal disc showing cell boundaries marked with Ecad∷GFP. The developing wing pouch has been segmented using ImageJ. The genotype of the Drosophila used is yw;;dECad::GFP (BL# 46556) (B) A pouch constructed computationally using EpiTools that served as a basis for Ca2+ signaling simulations. In brief, cells were segmented from a wing disc. Centroids of segmented cells were used to define cellular positions in the simulated wing disc. A Voronoi tessellation followed by multiple rounds of Lloyd’s relaxation [72] was used to define a template wing disc that matches the experimentally observed network topology. (TIF) [file pcbi.1009543.s001.tif]

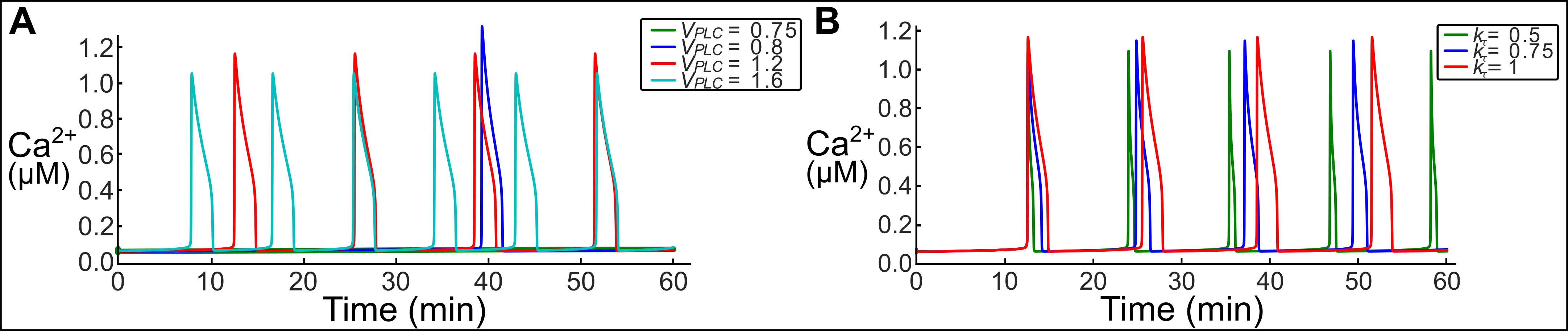

Supplement: S2 Fig — The model was calibrated to match experimental single-cell frequency and amplitude. Perturbations to stimulation strength VPLC (A) alters the frequency and amplitude of Ca2+ oscillations whereas perturbations to kτ (B) only alters the frequency. (TIF) [file pcbi.1009543.s002.tif]

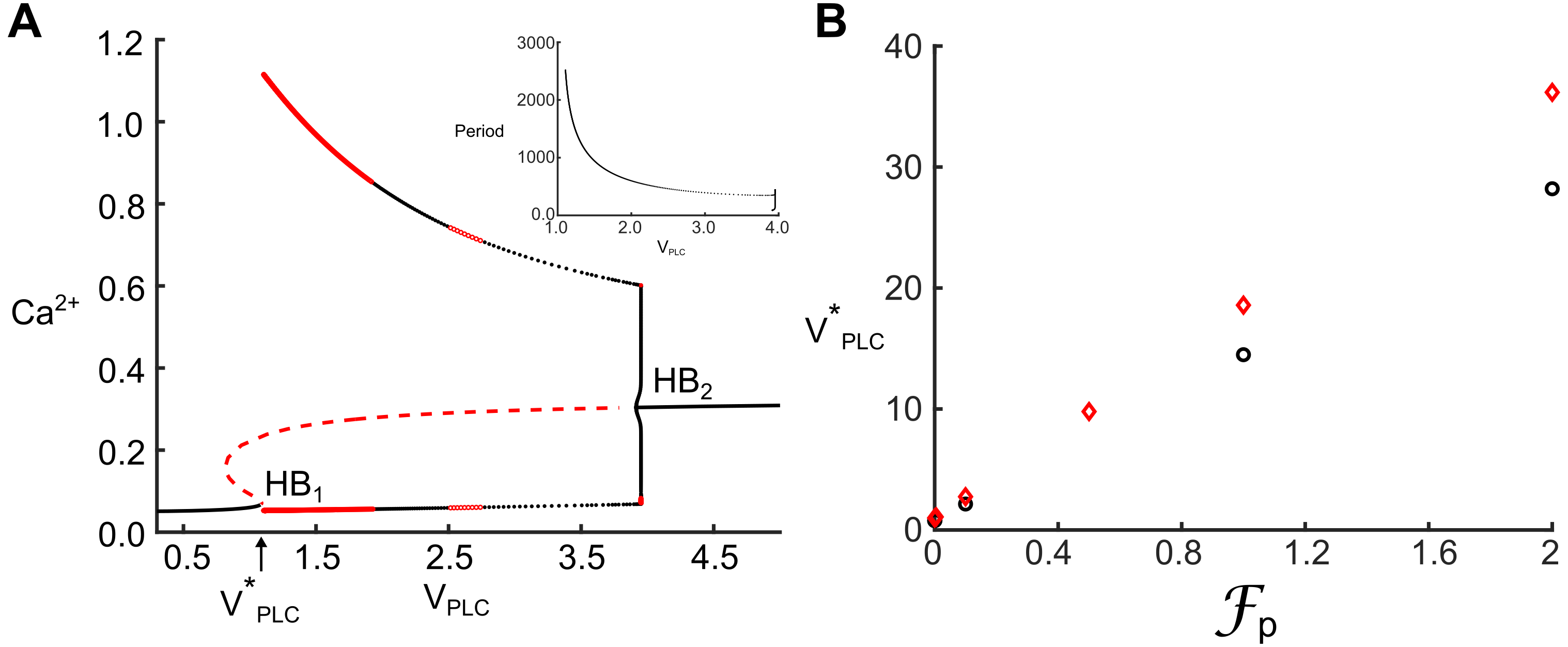

Supplement: S3 Fig — (A) Bifurcation diagram for the modified model used in this study; shown are the maxima and minima of the Ca2+ oscillations (dots) and the Ca2+ steady states (solid and dashed lines) as a function of the stimulus (VPLC). Solid and dashed lines in red indicate stable and unstable states, respectively. Red dots indicate the maxima and minima of unstable limit cycle and the black dots indicate maxima and the minima of the stable limit cycle. HB, Hopf bifurcation occurs when VPLC is varied. Inset figure shows the period of Ca2+ oscillations as a function of VPLC. (B) Blocking permeability of IP3, Fp via gap junctions decreases VPLC where the initial Hopf bifurcation point (HB1) occurs in the bifurcation diagram. Block dots indicate conditions where permeability of Ca2+, Fc is set to 0. Red diamonds indicate conditions where Fc is set to 0.5. (TIF) [file pcbi.1009543.s003.tif]

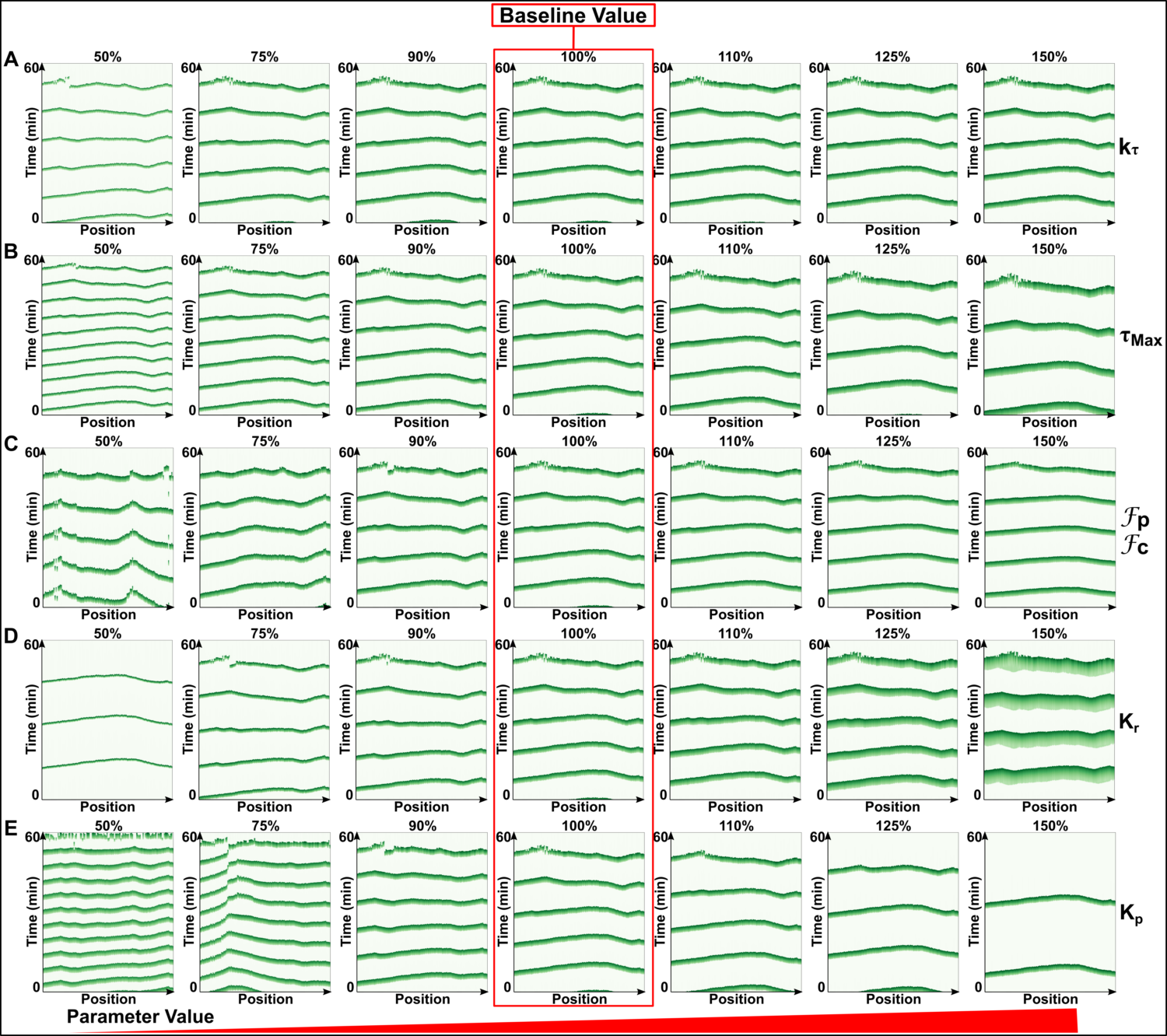

Supplement: S4 Fig — Five different parameters in the 2D model were varied from their baseline values (BV). VPLC profiles of simulated tissues were selected to generate intercellular waves (red box) and are identical across all simulations to enable comparisons. Each row represents one parameter being varied in a scaling manner by fixed percentages listed in each column (i.e., 50% of a BV of 1.5 μM would simulate a value of 0.75 μM). Simulations were performed varying only one parameter while holding all others constant at their BVs. Signal frequency is observed through number of bands in the kymograph, and signal duration is observed through thickness of the bands in the kymograph. (A) Decreased kτ (BV of 1.5 μM) increased frequency and decreased duration of the Ca2+ signal whereas increased kτ did not influence the signal. (B) Decreased τmax (BV of 800 s-1) increased frequency and decreased duration of the Ca2+ signal whereas increased τmax decreased frequency and increased duration. (C) Decreased gap junction (GJ) communication Fp/c (BVs of 0.005 μM2 s-1 for Fp; 0.0005 μM2 s-1 for Fc) decreased propagation of the Ca2+ signal whereas increased GJ communication increased propagation. Signal propagation is visualized by the uniformity of the signal across the tissue. (D) Decreased Kr (BV of 0.4 μM) decreased frequency and duration of the Ca2+ signal whereas increased Kr decreased frequency but increased duration. (E) Decreased Kp (BV of 0.13 μM) increased frequency of the Ca2+ signal whereas increased Kp decreased frequency. (TIF) [file pcbi.1009543.s004.tif]

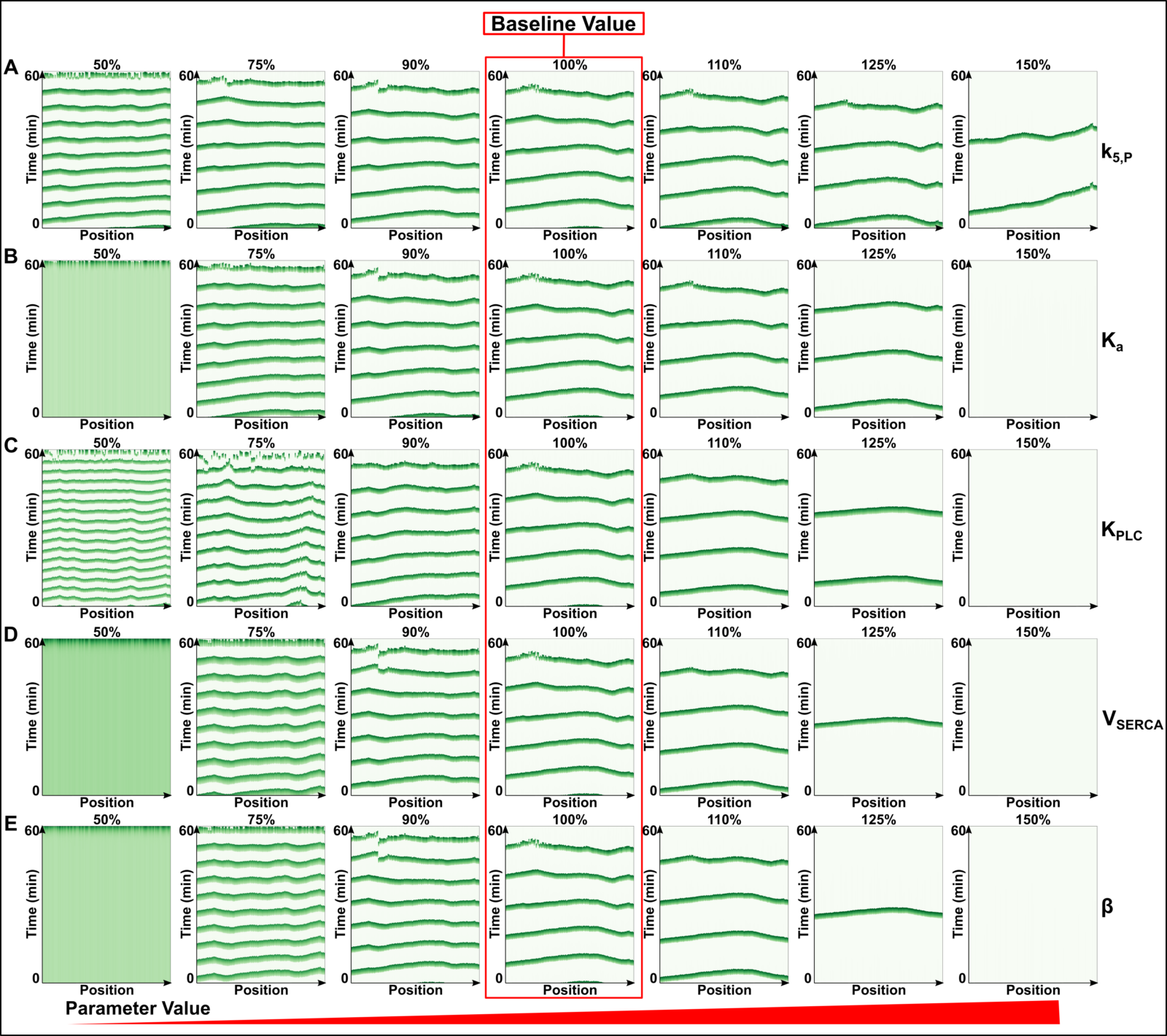

Supplement: S5 Fig — Five different parameters in the 2D model were varied from their baseline values (BV). VPLC profiles of simulated tissues were selected to generate intercellular waves (red box) and are identical across all simulations to enable comparisons. Each row represents one parameter being varied in a scaling manner by fixed percentages listed in each column (i.e., 50% of a BV of 0.66 s-1 would simulate a value of 0.33 s-1). Simulations were performed varying only one parameter while holding all others constant at their BVs. Signal frequency is observed through number of bands in the kymograph, and signal duration is observed through thickness of the bands in the kymograph. (A) Decreased k5,P (BV of 0.66 s-1) increased frequency of the Ca2+ signal whereas increased k5,P decreased frequency. (B) Decreased Ka (BV of 0.08 μM) increased frequency of the Ca2+ signal to the point of observing constant activity, whereas increased Ka decreased frequency to the point of loss of signal in a 60 minute simulation. (C) Decreased KPLC (BV of 0.2 μM) increased frequency of the Ca2+ signal whereas increased KPLC decreased frequency to the point of loss of signal in a 60 minute simulation. (D) Decreased VSERCA (BV of 0.9 μM s-1) increased frequency and duration of the Ca2+ signal to the point of observing constant activity, whereas increased VSERCA decreased frequency to the point of loss of signal in a 60 minute simulation. (E) Decreased β (BV of 0.185) increased frequency and duration of the Ca2+ signal to the point of observing constant activity, whereas increased β decreased frequency to the point of loss of signal in a 60 minute simulation. (TIF) [file pcbi.1009543.s005.tif]

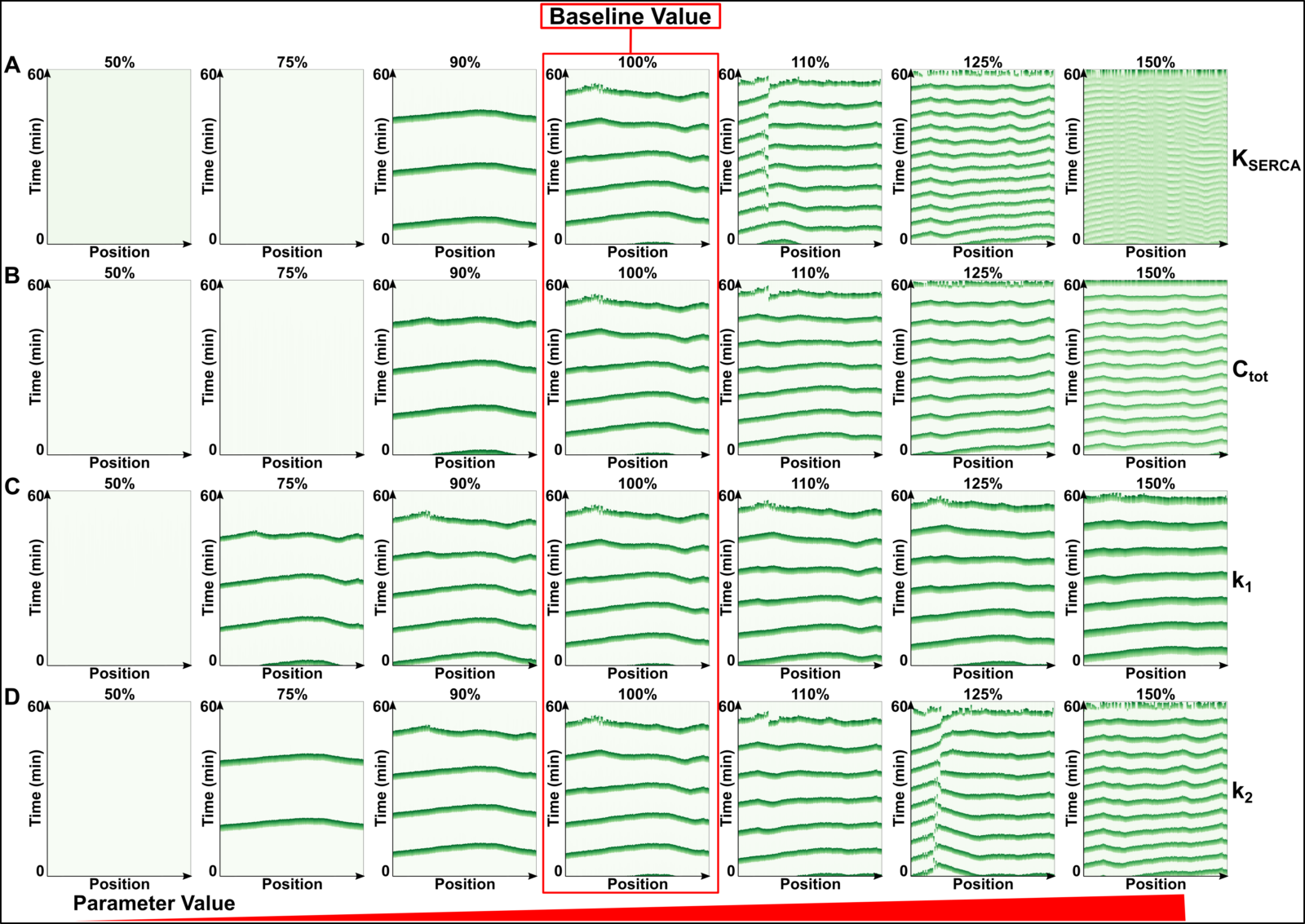

Supplement: S6 Fig — Four different parameters in the 2D model were varied from their baseline values (BV). VPLC profiles of simulated tissues were selected to generate intercellular waves (red box) and are identical across all simulations to enable comparisons. Each row represents one parameter being varied in a scaling manner by fixed percentages listed in each column (i.e., 50% of a BV of 0.1 μM would simulate a value of 0.05 μM). Simulations were performed varying only one parameter while holding all others constant at their BVs. Signal frequency is observed through number of bands in the kymograph, and signal duration is observed through thickness of the bands in the kymograph. (A) Decreased KSERCA (BV of 0.1 μM) decreased frequency of the Ca2+ signal whereas increased KSERCA increased frequency. (B) Decreased ctot (BV of 2 μM) decreased frequency of the Ca2+ signal whereas increased ctot increased frequency. (C) Decreased k1 (BV of 1.11 s-1) decreased frequency and duration of the Ca2+ signal whereas increased k1 increased frequency and increased. (D) Decreased k2 (BV of 0.0203 s-1) decreased frequency of the Ca2+ signal whereas increased k2 increased frequency. (TIF) [file pcbi.1009543.s006.tif]

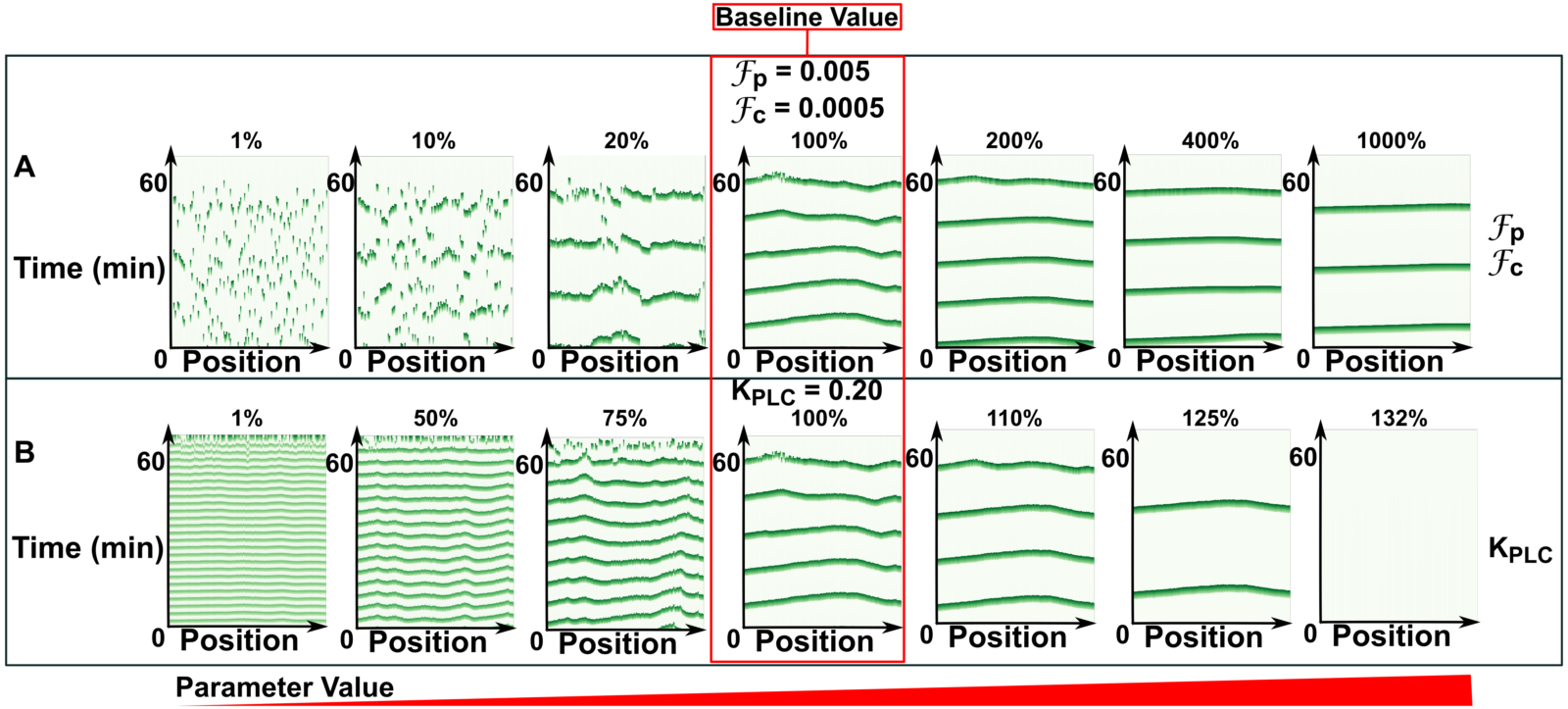

Supplement: S7 Fig — Two different parameters in the 2D model were varied from their baseline values (BV). VPLC profiles of simulated tissues were selected to generate intercellular waves (red box) and are identical across all simulations to enable comparisons. Each row represents one parameter being varied in a scaling manner by fixed percentages listed in each column (i.e., 50% of a BV of 0.1 μM would simulate a value of 0.05 μM). Simulations were performed varying only one parameter while holding all others constant at their BVs. Signal frequency is observed through number of bands in the kymograph, and signal duration is observed through thickness of the bands in the kymograph. (A) GJ permeability of IP3 and Ca2+ influences synchronization of Ca2+ signaling among cells. Decreased gap junction (GJ) communication Fp/c (BVs of 0.005 μM2 s-1 for Fp; 0.0005 μM2 s-1 for Fc) results in a transition of intercellular waves to intercellular transients, and to single-cell spikes. Increased GJ communication increased Ca2+ signal propagation and decreased signal frequency. Signal propagation is visualized by the uniformity of the signal across the tissue. (B) Variations in the half-activation of VPLC term, KPLC (BV of 0.2 μM), only changed the frequency of the ICWs. (TIF) [file pcbi.1009543.s007.tif]

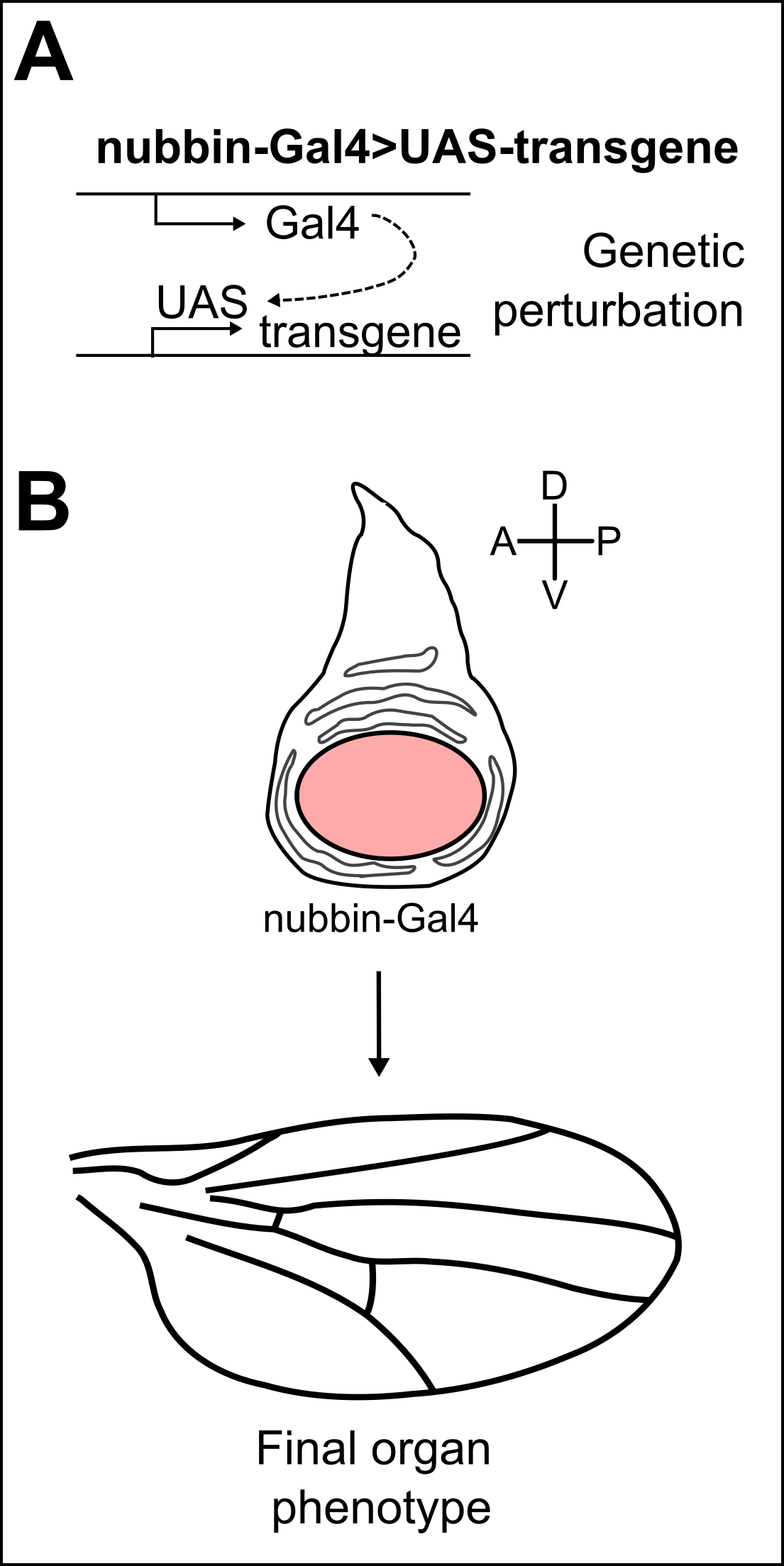

Supplement: S8 Fig — (A) The GAL4/UAS system was used to express GCaMP6f transgene along with other transgenes. (B) nubbin is expressed in the wing disc pouch and the adult wing phenotype provide a readout of final phenotype after transgene expression in the wing disc pouch during larval stage. (TIF) [file pcbi.1009543.s008.tif]

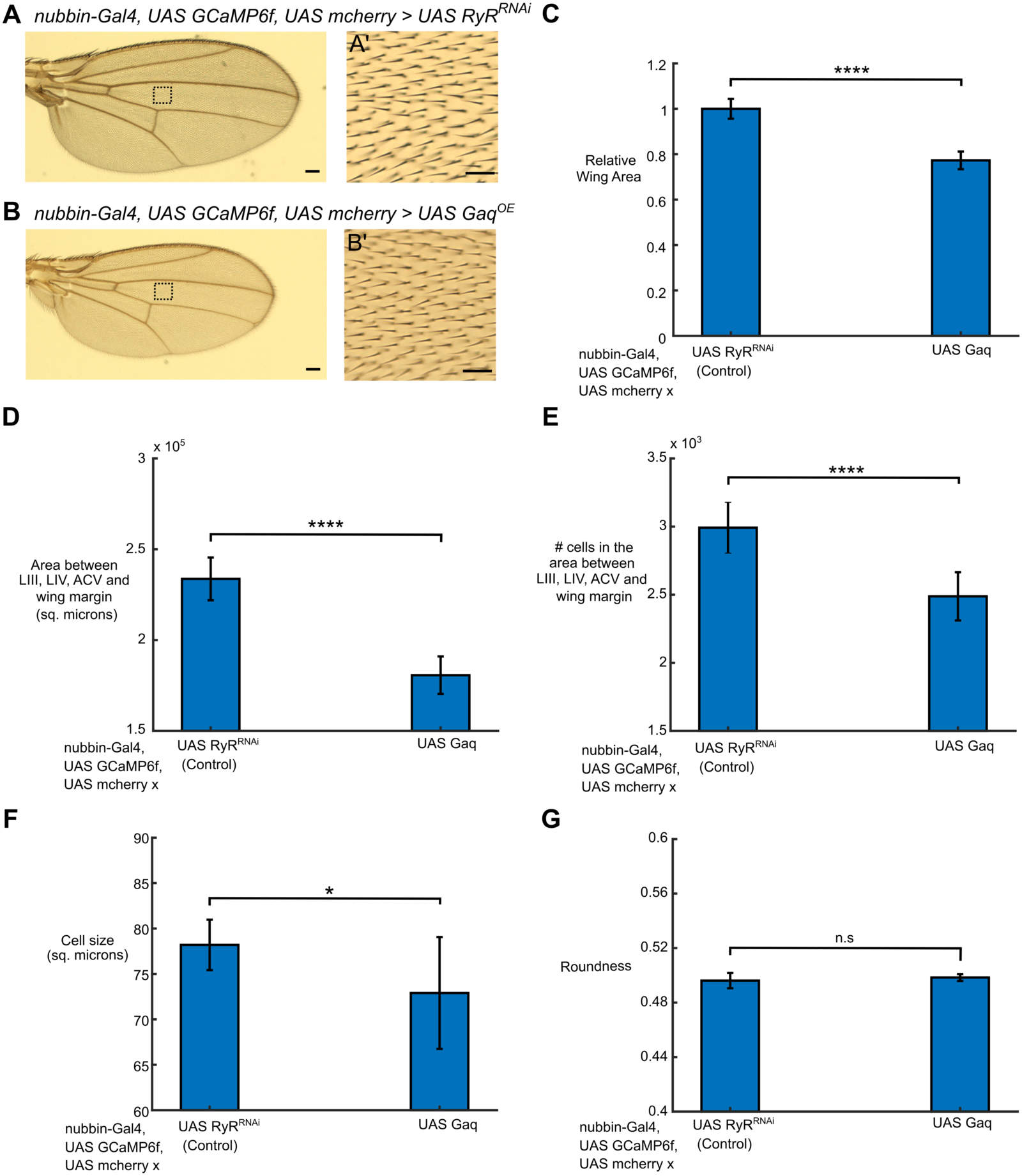

Supplement: S9 Fig — (A-B) Wings from adult males expressing RyRRNAi and wild type Gαq splice 3 variant with nubbin-Gal4, UAS GCaMP6f, UAS mcherry. (A’-B’) Region of interest (ROI) where the total number of setae was calculated. (C-F) Quantification of the wing size defined here as the area bounded by LIII, LIV, ACV and the wing margin, total cell number and cell area. Overexpression of Gαq in the pouch results in a decrease in total wing area, cell number and cell size. 10 samples were analyzed per condition. Error bars represent standard deviation. (G) Quantification of roundness of the adult wing. Gαq overexpression does not affect the roundness. Student t-test was used for statistical significance testing. (TIF) [file pcbi.1009543.s009.tif]

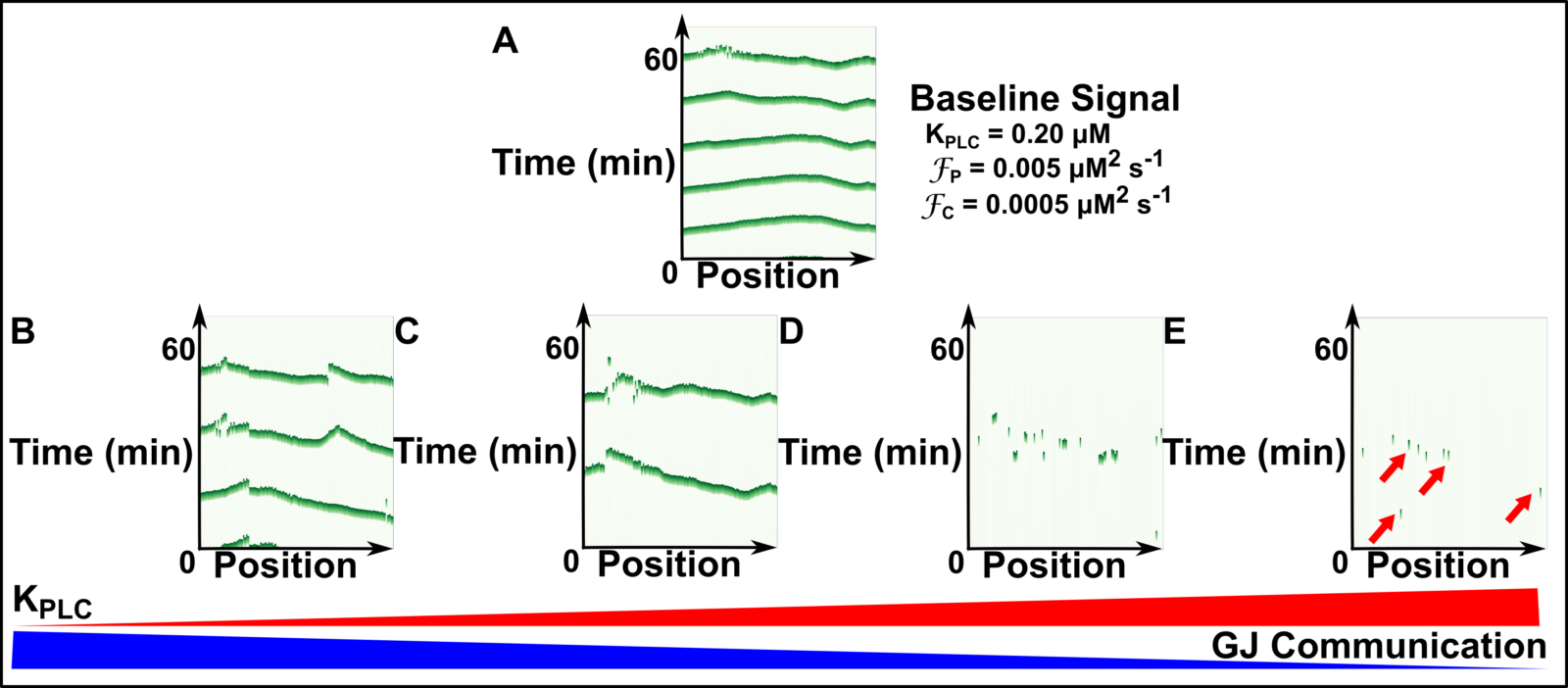

Supplement: S10 Fig — To replicate the ex vivo observations of insulin inducing single-cell Ca2+ spikes, GJ permeability and the half-activation of VPLC were varied simultaneously in silico. (A) A baseline intercellular wave was used as the comparison for how parameter variations changed signal with the following parameter values: IP3 gap junction permeability (Fp) of 0.005 μM2s-1, Ca2+ gap junction permeability (Fc) of 0.0005 μM2s-1, and KPLC of 0.20 μM. (B-E) KPLC is increased left-to-right (red bar), and gap junction communication is decreased left-to-right (blue bar). An increase in KPLC results in a decrease in frequency, while decrease in gap junction communication results in single-cell Ca2+ spikes (red arrows). (TIF) [file pcbi.1009543.s010.tif]

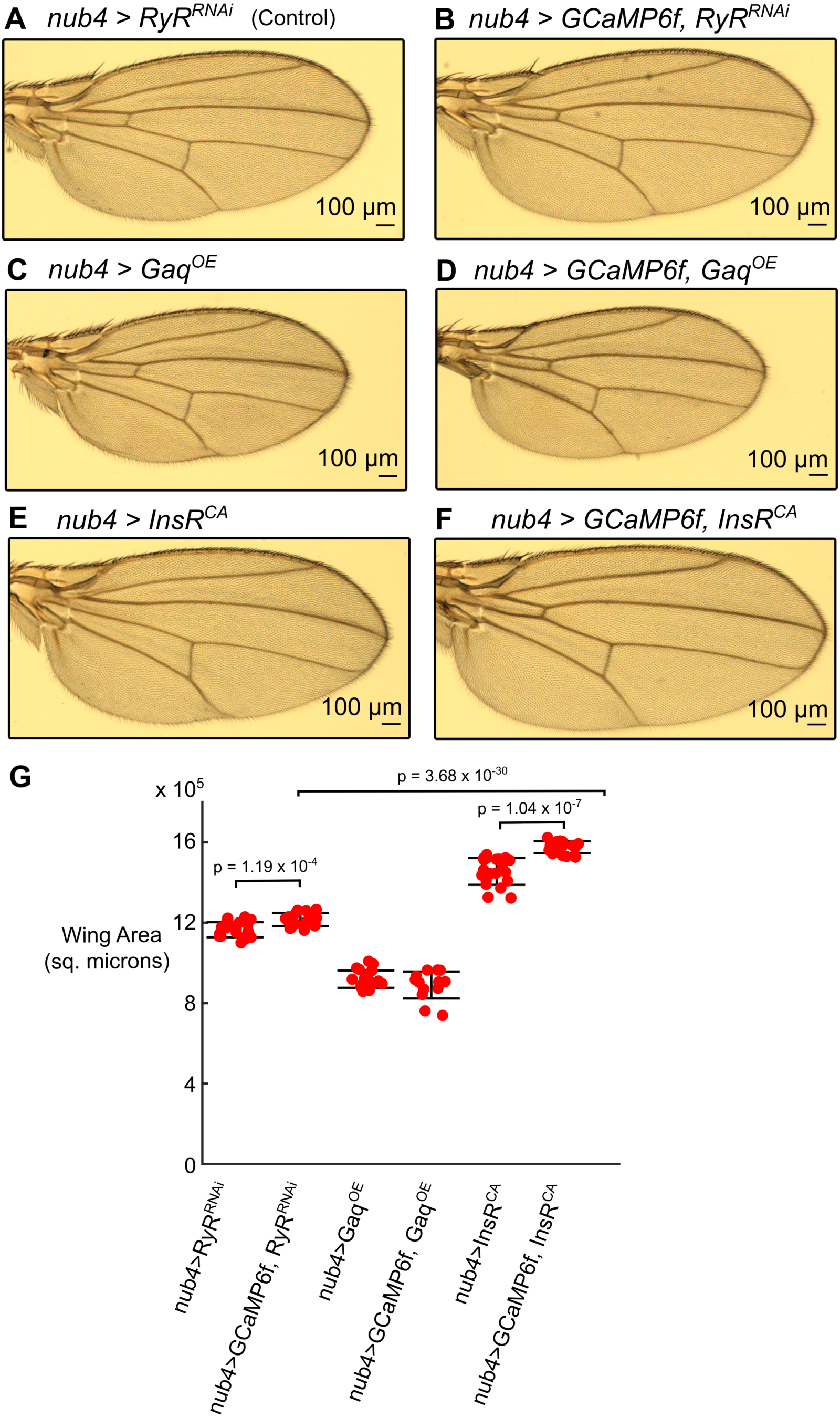

Supplement: S11 Fig — (A-F) Wings from adult males with the indicated crosses. (A) nubbin-GAL4 x UAS-RyRRNAi (i.e., nub4>RyRRNAi), (B) nubbin-GAL4, UAS-GCaMP6f x UAS-RyRRNAi (i.e., nub4>GCaMP6f, RyRRNAi), (C) nubbin-GAL4 x UAS-GaqOE embryonic splice 3 variant of Gaq (i.e., nub4>GaqOE), (D) nubbin-GAL4, UAS-GCaMP6f x UAS-GaqOE (i.e., nub4>GCaMP6f, GaqOE), (E) nubbin-GAL4 x UAS-InsRCA (i.e., nub4>InsRCA) gain of function mutant where the α subunit is partially deleted. (F) nubbin-GAL4, UAS-GCaMP6f x InsRCA (i.e., nub4>GCaMP6f, InsRCA). (G) Quantification of adult wings. The genetic encoded calcium sensor, GCaMP6f, binds to Ca2+ with high affinity, thus expression of the sensor will to some degree act as a sponge of cytosolic Ca2+. Interestingly, the presence of the GCaMP6f sponge with constitutively activated insulin signaling increases the adult wing size (E, F). Similar enhancement of wing size was observed in control wings when GCaMP6f sensor was expressed (A, B). No significant change in the adult wing size was observed when Gαq was overexpressed, suggesting sponging effects are trivialized under Gαq overexpression (C,D). Unpaired student t-test was used, and the p-values are indicated above. (TIF) [file pcbi.1009543.s011.tif]

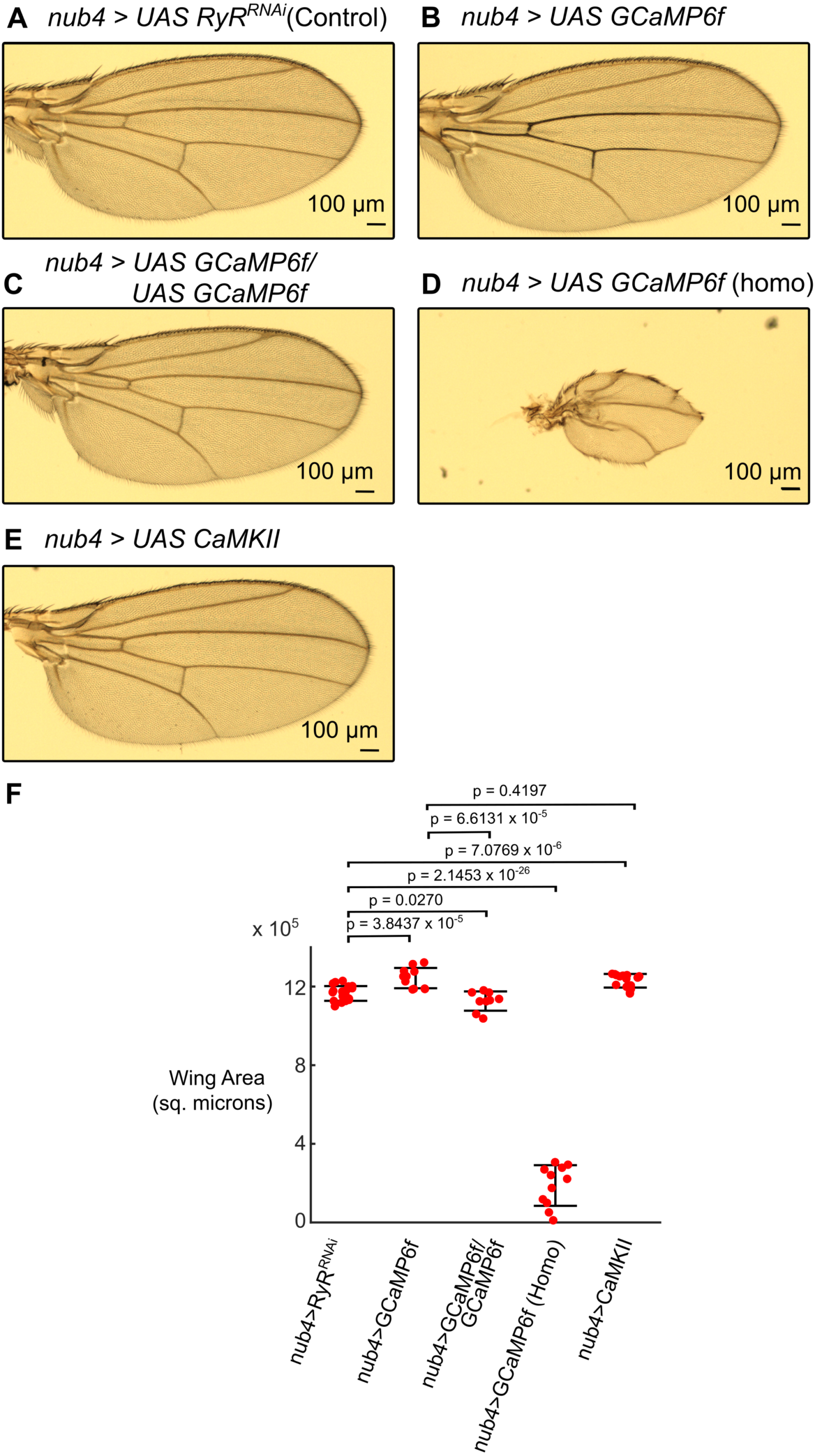

Supplement: S12 Fig — (A-E) Wings from adult males of indicated genotypes (A) nubG4, UAS RyRRNAi, (B) nubG4, UAS GCaMP6f, (C) nubG4, UAS GCaMP6f/UAS GCaMP6f, (D) nubG4, UAS GCaMP6f (Homozygous) (E) nubG4, UAS CaMKII (F) Quantification of adult wing sizes. As the gene dose of GCaMPf is increased in the wing disc, the overall wing area decreases in size (B, C, D). Overexpressing possible Ca2+ downstream target CaMKII increases the wing size consistent with B in which one copy of GCaMP6f was expressed. (TIF) [file pcbi.1009543.s012.tif]

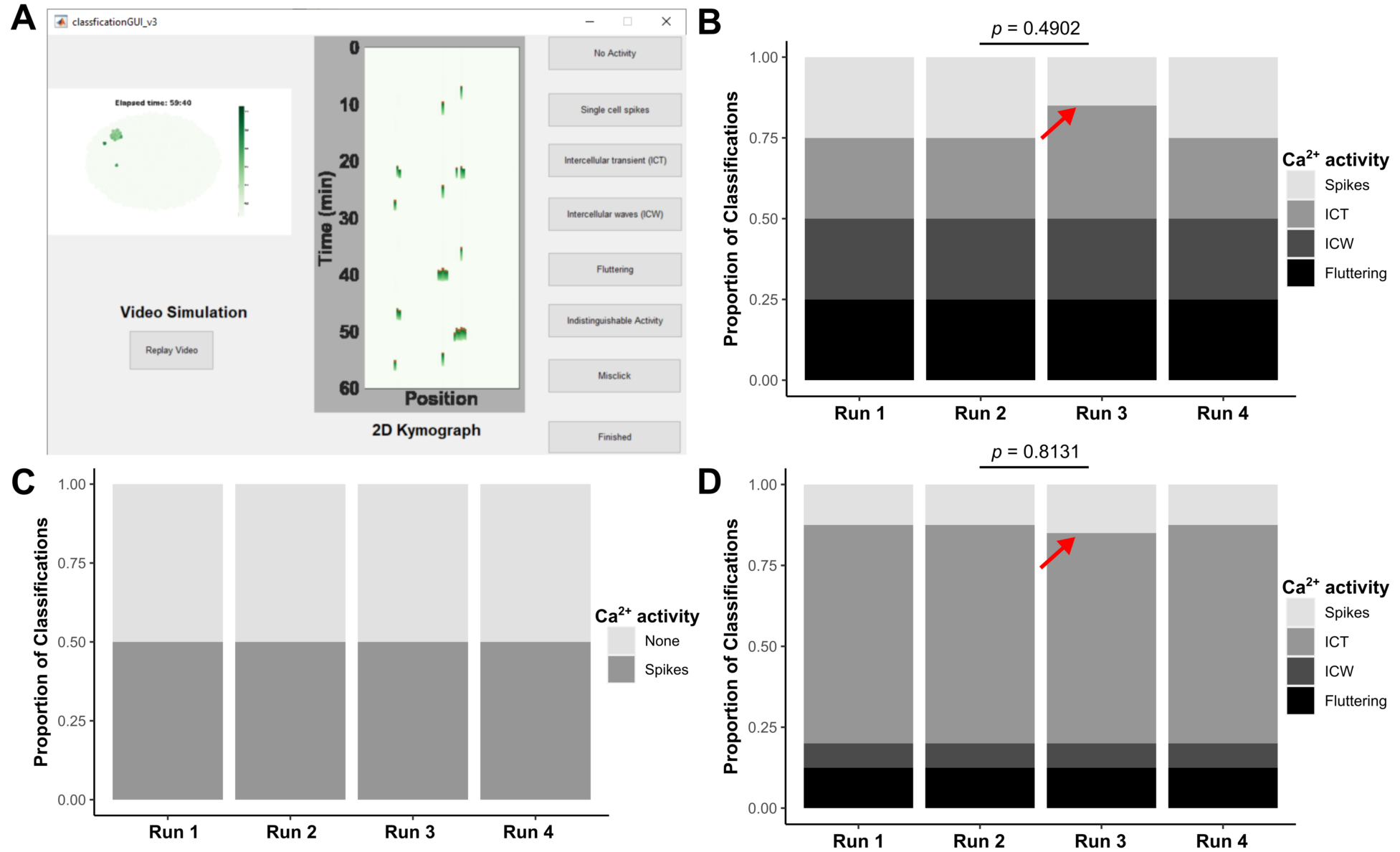

Supplement: S13 Fig — Simulations corresponding to the main text figure conclusions (i.e., Figs 2–4) were repeated five separate times with five different random number generator seeds. The random number generator seed value determines which cells in a simulated tissue are determined to be initiator cells. For the case of Fig 2, the VPLC values of standby cells also rely on the random number generator seed as the values are sampled randomly from a uniform distribution with set boundaries. The simulations’ resulting video and image outputs were randomized and their inputs were hidden to allow classification of the Ca2+ activity. (A) Using a graphical user interface (GUI) in MATLAB, the randomized video and kymograph simulations were drawn to show the output kymograph and play the video simulation. A user was tasked to classify the activity of the simulation output as having no activity, single-cell spikes, intercellular transient activity (ICT), intercellular wave activity (ICW), or global fluttering activity. For each main text figure, the five separate simulations had their Ca2+ activity classified in four independent runs. Each run corresponds to a brand new running of the classification GUI, each with a different randomization scheme to display the outputs of the simulations. (B) The proportions of Ca2+ activity are plotted for Fig 2‘s repeated simulations. Runs 1, 2, and 4 all had the same proportions, indicating reproducibility of the simulations’ outputs. Run 3 had mismatched classifications between the ICT and spike class (red arrow), however, the difference in proportions was not significant using a proportions test without a continuity correction [73–75]. (C) The proportions of Ca2+ activity are plotted for Fig 3‘s repeated simulations. Because Fig 3 was designed to demonstrate either no activity in the case of enabled gap junction communication, or spiking activity in the case of disabled gap junction communication, only two classes of activity appear. In each classification ru [file pcbi.1009543.s013.tif]
