# Supplementary material for: From spikes to intercellular waves: Tuning intercellular calcium signaling dynamics modulates organ size control
Source: PLoS Comput Biol. 2021 Nov 1;17(11):e1009543. doi: 10.1371/journal.pcbi.1009543 (PMC8601605; doi:10.1371/journal.pcbi.1009543)
Supplement: S1 Table — (DOCX) [file pcbi.1009543.s014.docx]

**S1 Table. Extended data movies.**

| **SI Movie #** | **Description** |
| --- | --- |
| 1 | *nub-Gal4>UAS-GCaMP6f*, *UAS-mcherry*, ex vivo, spike |
| 2 | *nub-Gal4>UAS-GCaMP6f*, *UAS-mcherry*, ex vivo, ICT |
| 3 | *nub-Gal4>UAS-GCaMP6f*, ex vivo, ICW |
| 4 | *nub-Gal4>UAS-GCaMP6f*, ex vivo, fluttering |
| 5 | *nub-Gal4>UAS-GCaMP6f*, in vivo, spikes |
| 6 | *nub-Gal4>UAS-GCaMP6f*, in vivo, ICT |
| 7 | *nub-Gal4>UAS-GCaMP6f*, in vivo, ICW |
| 8 | *nub-Gal4>UAS-GCaMP6f*, in vivo, fluttering |
| 9 | Spike, Simulation output |
| 10 | ICT, Simulation output |
| 11 | ICW, Simulation output |
| 12 | fluttering, Simulation output |
| 13 | *nub-Gal4>UAS-GCaMP6f*, ex vivo in Grace’s low 20E media, gap junctions not blocked (Control) |
| 14 | *nub-Gal4>UAS-GCaMP6f*, ex vivo in Grace’s low 20E media with Carbenoxolone, gap junctions blocked |
| 15 | *nub-Gal4>UAS-GCaMP6f*, *UAS-RyR^RNAi^*, ex vivo in Grace’s low 20E media (Control) |
| 16 | *nub-Gal4>UAS-GCaMP6f*, *UAS-InsR^CA^*, ex vivo in Grace’s low 20E media |
| 17 | *nub-Gal4>UAS-GCaMP6f*, *UAS-InsR^DN^*, ex vivo in Grace’s low 20E media |
| 18 | *nub-Gal4>UAS-GCaMP6f*, *UAS-Gaq^OE^*, ex vivo in Grace’s low 20E media |
