# Supplementary material for: From spikes to intercellular waves: Tuning intercellular calcium signaling dynamics modulates organ size control
Source: PLoS Comput Biol. 2021 Nov 1;17(11):e1009543. doi: 10.1371/journal.pcbi.1009543 (PMC8601605; doi:10.1371/journal.pcbi.1009543)
Supplement: S2 Table — Note that maximal deviations wing size for strong growth perturbations is in range of 20–50%. References for this table include this study, [19], [76], [77]. (DOCX) [file pcbi.1009543.s015.docx]

**S2 Table.** Changes in wing area for known perturbations through GAL4/UAS system. Note that maximal deviations wing size for strong growth perturbations is in range of 20-50%.

| **Pathway** | **Perturbation** | **Genotypes of perturbations** | **Genotypes of Control** | **% Changes in wing area** | **References** |
| --- | --- | --- | --- | --- | --- |
| Insulin | InsR^CA^ (Upregulation) | *nubGal4>UAS-GCaMP6f, UAS-InsRCA* | *nubGal4>UAS-GCaMP6f, UAS-mcherry* | 29% | This study |
|  | InsR^DN^ (Downregulation) | *nubGal4>UAS-GCaMP6f, UAS-InsRDN* | *nubGal4>UAS-GCaMP6f, UAS-mcherry* | -49% |  |
| Ca^2+^ | Gαq^OE^ | *MS1096Gal4 > UAS-GαqOE* | *MS1096Gal4 > UAS-RyRRNAi* | -20% | (1) and this study |
|  | Gαq^RNAi^ | *MS1096Gal4 > UAS-GαqRNAi* | *MS1096Gal4 > UAS-RyR^RNAi^* | -17% |  |
|  | itp-83A^RNAi^ | *MS1096Gal4 > UAS-itpr-83ARNAi* | *MS1096Gal4 > UAS-RyRRNAi* | -11% |  |
|  | inx2^RNAi^ | *MS1096Gal4 > UAS-inx2RNAi* | *MS1096Gal4 > UAS-RyRRNAi* | -29% |  |
|  | sI^RNAi^ | *MS1096Gal4 > UAS-sIRNAi* | *MS1096Gal4 > UAS-RyRRNAi* | -13% |  |
|  | plc21c^RNAi^ | *MS1096Gal4 > UAS-plc21CRNAi* | *MS1096Gal4 > UAS-RyRRNAi* | -3% |  |
| Hippo | exRNAi | *nubGal4>UAS-exRNAi* | *nubGal4* | 60% | (2) |
|  | Kib | *nubGal4>UAS-kib* | *nubGal4* | -20% |  |
| Mechanical | rok^RNAi^ | *Nub-Gal4>UAS-dcr2, UAS-rok^RNAi^* | *nubGal4>UAS-dcr2* | -20% | (3) |
|  | rok.CAT | *nubGal4>UAS-dcr2, UAS-rokCAT* | *nubGal4>UAS-dcr2* | 12% |  |

**References**

1. Brodskiy PA, Wu Q, Soundarrajan DK, Huizar FJ, Chen J, Liang P, et al. Decoding calcium signaling dynamics during *Drosophila* wing disc development. Biophysical journal. 2019;116(4):725–40.

2. Su T, Ludwig MZ, Xu J, Fehon RG. Kibra and Merlin Activate the Hippo Pathway Spatially Distinct from and Independent of Expanded. Developmental Cell. 2017 Mar 13;40(5):478-490.e3.

3. Rauskolb C, Sun S, Sun G, Pan Y, Irvine KD. Cytoskeletal Tension Inhibits Hippo Signaling through an Ajuba-Warts Complex. Cell. 2014 Jul 3;158(1):143–56.
