# Supplementary material for: From spikes to intercellular waves: Tuning intercellular calcium signaling dynamics modulates organ size control
Source: PLoS Comput Biol. 2021 Nov 1;17(11):e1009543. doi: 10.1371/journal.pcbi.1009543 (PMC8601605; doi:10.1371/journal.pcbi.1009543)
Supplement: Attachment — Submitted filename: Comments.pdf [file pcbi.1009543.s034.pdf]

Soundarrajan et al. proposed a work to study the relation between Ca2+ signaling dynamics and size control in Drosophila imaginal wing discs utilizing both computational and experimental approaches. They showed the key mechanisms in formation of four distinct Ca2+ patterns, and how these mechanisms are related to disc growth. Conversely they showed Ca2+ activity is required to control growth of wing disc. The conclusions are exciting since they answer a long-term question in developmental biology: how growth and pattern interact with each other.

However, I failed to see the valid analysis on the mechanisms how growth regulates Ca2+ pattern. The mechanisms how Ca2+ regulates growth are unknown, but it is acceptable for such a more difficult question. The authors should at least clearly address the prior question with significant improvement on their arguments, as well as the readability. Here are my comments:

1. Although details of the computational model seem to be explained in the supplement, it would be nice to have a brief discussion in the main text to help readers understand the model better. And many clarifications are needed:

a. Fig 1B-C: It is unclear what do the spatial axes X, Y and L represent in the wing disc.
b. Fig 1D: The meaning of each annotation should be further explained. Why GPCRs and RTKs use the same annotation, and same issue for PLCϒ and PLCβ. Both GJ and IP3R are represented by white box, are they the same thing?
c. Fig 1D and 1E: There are many regulatory mechanisms, especially for Ca2+. The regulatory mechanisms and their corresponding formulations need to be clearly explained.
d. Is FEX concentration related to VPLC in the computational model? FEX and VPLC levels are shown in different colors in Fig1 and Fig2. It may be good to point out they are the same thing. Maybe using the same color or adding some extra texts in the figures to highlight it?
e. It is unclear how GJ affect permeability of Ca2+ and IP3. Is it also a component in the equations or it is only a parameter for permeability?
f. In Fig 2A-2D, is it possible to show the four distinct patterns in the same way in Fig 1C?
g. In Fig 2A-2D: It seems cell with VPLC above the Hopf bifurcation is initiator cell. Fig 2E shows all cells are standby cells in Spikes, ICT and ICW, and all cells are initiator cells in fluttering. But why Fig 2A-2C still have initiator cells (I assume the dark blue points are initiator cells? May have an annotation pointing this out when the Input profile is presented). Also, statistical meaning of the bar and box in Fig 2E should be given in the figure legend.
h. Is the tissue growth explicitly modeled or only modeled in various fixed size tissue?

2. Line 314-343: the authors proposed two hypotheses to explain how wing disc growth regulates Ca2+ pattern. Then they used both computational models and

experiments to eliminate one hypothesis. However, I found this argument lacks of evidence.

a. What is the difference observed in these two hypotheses in computational model? As I can tell, the only difference is that they have different numbers of initiator cells at initial point. I don't understand how experiment helps eliminate one of them? How can experiment identify initiator cells? Experimental observations are shown in Fig 4C-4D, and where is the consistence stated in Line 337-339 being found comparing to the computational model?
b. How parameters or any other conditions are perturbed in the two cases for computational model? The relation between number of initiator cells and disc size, and the relation between permeability and disc size in two cases should be given.
c. In the first scenario, how to get decreasing fraction of initiator cells? If it is manipulated by decreasing VPLC, different decreasing rate of VPLC may lead to different observations. The authors should give a more systematic analysis to show how generally decreased number of initiator cells leads to decreased Ca2+ activity.
d. All conclusions from simulations should be made from multiple independent repeats and their statistics, instead of a single simulation.

3. Line 381-383: The authors only showed insulin affect Ca2+ functions. It is completely unknown how insulin interacts with the system the authors modeled. The perturbations on two parameters for mimicking experimental manipulation on insulin make no sense.

4. Many mistakes and missing information throughout this entire manuscript:

a. Figure numbers are incorrect in the last two sections in Results, such as Line 350, Line 355, line 372, etc.
b. Line 194: Something wrong in this equation.
c. Information for Tissue model is not given in Supporting Information (Line 575).
d. Information for multicellular model is not given in Supporting Information as stated in Line 579-580.
e. Line 552: how many discs were collected and quantified for simulations? In simulation, how many real discs were used to calculate one computational domain?
f. ctot value is not given in Table S1.

5. I don't understand what was summarized in Fig 5H. Arrows point to everywhere without any specific meaning.

6. Many aspects of the model formation need to be clarified:

a. In the computational model, only randomness in PLC production is considered while other components are in deterministic description. Any

rationale? The Ca2+ concentration may also be largely perturbed in different cells.

b. In the single-cell model, only IP3 has degradation rate. Any explanations why degradation is not considered for IP3R and Ca2+? From the value of k5,p, The half-life for IP3 is 0.46 sec, which is extremely fast, explanation? The degradation term for p in Fig 1E should be negative.
c. Equation c for Cytosolic [Ca2+] in Fig 1E, it is not common to have a cube (second term) on the Michaelis-Menten term. Any interpretation?
d. In Table S1, authors should point out clearly the source for each parameter. If the parameter is from this work, how they were selected should be stated. A systematic sensitivity analysis for all parameter is necessary.
e. In one simulation, is the value of VPLC randomly perturbed in each cell, and is it dynamically perturbed during the simulation or only at the initial state? The ranges of VPLC in each phenotype are not pointed out in Table S1 (as stated in line 198).

7. Explanations on functions of each experimental manipulation would be helpful to readers, such as expression region for each GAL4/UAS systems and function of GCaMP6f sensor.

8. When the authors point to Supporting Information, it would be nice to point out the specific sections.
