# Supplementary material for: From spikes to intercellular waves: Tuning intercellular calcium signaling dynamics modulates organ size control
Source: PLoS Comput Biol. 2021 Nov 1;17(11):e1009543. doi: 10.1371/journal.pcbi.1009543 (PMC8601605; doi:10.1371/journal.pcbi.1009543)
Supplement: Attachment — Submitted filename: Review-ZartmanEtAlPaper.pdf [file pcbi.1009543.s035.pdf]

Review: *From spikes to intercellular waves: tuning intercellular Ca2+ signaling dynamics modulates organ size control*

The authors present a systems-level description of Ca2+ 94 signaling during organ development which has been lacking in the literature. This is an important contribution since Ca2+ signalling plays a crucial role in development but not enough modelling has been done in this direction to elucidate many open questions. The authors conduct a bifurcation analysis/parameter sensitivity analysis of the model by altering key parameters and find four types of Ca2+ signals which are also observed in experiments. I recommend publication with minor revisions. I list the revisions requested below:

- L62-63: I find very interesting the finding "Our experiments also reveal that a "goldilocks zone" of optimal Ca2+ 63 activity is required to achieve optimal growth at the organ level". The same optimal window has been observed in other Ca2+ signalling scenarios, for example for fertilizing the egg successfully. Maybe refer to this phenomenon more generally in other parts of the text.
- L81: "Epithelial cells do not observe social distancing" – although I found this sentence amusing in a way as it is obviously related to the pandemic I also find it detracting from the context of the embryogenesis
- L.192: "We modified the rate of IP3R inactivation term, r, to replicate our experimental Ca2+ dynamics".
- Some more explanations to be provided about the modelling process here and also in the Supplementary Information where the model is explained. How was the particular choice of the model and the parameters chosen motivated by your experiments? More specifically, please justify in more detail why the Ca2+ signalling model by Politi et al (2006) was chosen versus many other options. Does it provide the full spectrum of qualitative behaviours observed in experiments perhaps?
- L. 198 (and earlier): "To reproduce the four distinct patterns in silico, we varied the VPLC parameter progressively in individual cells across a range of parameters (Table S1)." Since VPLC is a very important bifurcation parameter I strongly suggest to explain it in more detail in the main text.
- L. 250: "VPLC values set to the Hopf bifurcation threshold value of 0.774". Please explain where 0.774 is coming from/why it is chosen.
- Figure S4: In the bifurcation analysis why only V_PLC and F_P chosen as bifurcation parameters. Could you also add bifurcation diagrams for τ_max, F_c, K_τ? (If there is a reason for not including these bifurcation diagrams please state it clearly.) Also, a 2-parameter bifurcation diagram could be useful.
- Please provide some more details about the algorithm/numerics of the cell-based model you have developed (in Supplementary Information). Also, it would be good to provide some more instructions so that the Python code can be easily run by other users (I have followed the GitHub link provided and read the "Readme" but have not run the code.)

Typographical errors and aesthetic/stylistic edits:

In the abstract: Ca 2+ -> Ca^{2+} as in main text

Recently we showed - > We have shown recently

When many references are listed, e.g. L106-7 please make sure they are in chronological order (or reverse order, depending on what the journal requires)

L121 and L123 “not characterised” appears in subsequent sentences, please choose a different phrase

L123: IP3R -> IP3?

L143: summary of baseline -> summary of **the** baseline

L. 194: the subscript “max” is missing in τ (See equation)
