# Supplementary material for: From spikes to intercellular waves: Tuning intercellular calcium signaling dynamics modulates organ size control
Source: PLoS Comput Biol. 2021 Nov 1;17(11):e1009543. doi: 10.1371/journal.pcbi.1009543 (PMC8601605; doi:10.1371/journal.pcbi.1009543)
Supplement: Attachment — Submitted filename: Review-ZartmanEtAlPaper_secondround.pdf [file pcbi.1009543.s037.pdf]

Review: *From spikes to intercellular waves: tuning intercellular Ca2+ signaling dynamics modulates organ size control*

The authors present a systems-level description of Ca2+ 94 signaling during organ development which has been lacking in the literature. This is an important contribution since Ca2+ signalling plays a crucial role in development but not enough modelling has been done in this direction to elucidate many open questions. The authors conduct a bifurcation analysis/parameter sensitivity analysis of the model by altering key parameters and find four types of Ca2+ signals which are also observed in experiments. I am pleased to see that the authors incorporated successfully changes addressing my previous review and that of the other reviewer. I still recommend publication with (very) minor revisions. I list the new revisions requested below. (I recommend that the authors, once they implement the changes below, that they do not need to send back the manuscript for a further check.)

ABSTRACT:

1. "Our computational model successfully recapitulates how the dynamics of Ca2+ transients varies during organ growth" Could you use "reproduces" instead of "recapitulates"?

MAIN TEXT:

2. L. 183 "we ~~assume~~ only consider the case…"

3. p.192: the sentence "where Fp refers to the permeability of IP3 through gap junction communication," does not make complete sense. You could say the "IP3 permeability of the gap junctions" – see also https://www.sciencedirect.com/science/article/pii/S0005273604000367?via%3Dihub if it helps.

4. p.193: "lj refers to the length of the cell boundary shared by cells i and cell j, respectively." The notation does not make complete sense to me – when you involve two cells it is natural that the subscripts of l should reflect that, i.e. shouldn't you use l_{ij}?

5. L.201: "In our model, we ignore the flux of Ca2+ 200 through the cell's plasma membrane," – please provide justification for this assumption

6. Please add English language articles where needed, such as "the" in many sentences. Examples (my correction with red):

- L. 178 "which is directly proportional to the PLC substrate PIP2"
- L.192-193: "…pj refers to the IP3 concentration in a neighboring cell j"
- L193: "lj refers to the length of the cell boundary"
- L.199: "pumped out from the cytosol to the extracellular…"

7. Please add punctuation marks in equations, e.g a comma is needed after equations when the sentence continues after the equation. For example, after equations (4), (5), (6), (7). Also please fix the capital in "Where" right after the power law for the initiator cells.

8. L.254: wholistic -> holistic

9. L. 244-245, L. 259-264

It is initially mentioned that the transition from limited to tissue-wide calcium activity depends on the amount of fly extract (FEX) added to the culture but then you vary V_{PLC}.Please explain

the dependence of V_{PLC} on the amount of FEX more precisely, this is still not clear in L. 259-264.

10. L. 286 “progression” is not the right word here. You could replace the bold-faced sentence in the beginning of the caption of Figure 2 as follows: “The level of hormonal stimulation governs the spatial extent of intercellular Ca2+ 286 communication”

11. please resolve inconsistencies in “single-cell” vs “single cell”. When single cell is used as a noun it does take a hyphen (e.g. in L340 please remove the hypehn). When it is used as an advective it does take a hyphen, e.g. L.55 “single-cell spikes”

12. L. 346 “necessary to ~~generating~~ generate Ca2+ activity in (embryonic epithelial?) cells.” Or is this a phenomenon that you assume exists in other cell types?

13. L. 369: “done” -> “taken”

14. L. 371-372: *“Decreased GJ permeability decreased propagation of the Ca2+ 371 signal across the simulated tissue 372 whereas increased GJ communication increased propagation*.” The second or first part of this sentence is redundant as they relay the same point.

15. L. 376 and L. 378: please quantify “substantial” using percentages.

16. L. 379: please adopt consistent notation you had used “GJ permeability” earlier in the text for “gap junctional permeability”. Also in L. 410 you use “Gap junction permeability”.

17. L.410: “shapes” ->”modulates”

18. “the power-law relationship value of -1.8 in scaling gap junction communication was 431 chosen to reflect the power-law relationship in our previous publication” please briefly remind the reader the reason for this choice, it is not obvious.

19. L. 435 “…activities of the tissues” -> why are these called activities? Please be consistent about the way you refer to V_{PLC} in the text. I also think it should be “of tissue”.
