# Supplementary material for: From spikes to intercellular waves: Tuning intercellular calcium signaling dynamics modulates organ size control
Source: PLoS Comput Biol. 2021 Nov 1;17(11):e1009543. doi: 10.1371/journal.pcbi.1009543 (PMC8601605; doi:10.1371/journal.pcbi.1009543)
Supplement: Attachment — Submitted filename: 2021 Rebuttal Letter V7 (dsoundar@nd.edu).docx [file pcbi.1009543.s036.docx]

**Response to reviewers:**

We thank the reviewers for helpful comments and for the positive recommendation for the article as suitable for *PLOS Computational Biology*. We have implemented all of the suggestions as detailed below in blue font for each point raised. The main changes in the manuscript text are also indicated in blue font.

We believe that these changes significantly strengthen the work and trust that it will be a valuable addition as an article in *PLOS Computational Biology*.

**Reviewer #1**

Soundarrajan et al. proposed a work to study the relation between Ca^2+^ signaling dynamics and size control in *Drosophila* imaginal wing discs utilizing both computational and experimental approaches. They showed the key mechanisms in formation of four distinct Ca^2+^ patterns, and how these mechanisms are related to disc growth. Conversely, they showed Ca^2+^ activity is required to control growth of wing disc. The conclusions are exciting since they answer a long-term question in developmental biology: how growth and pattern interact with each other.

Response: We thank Reviewer #1 for this positive assessment.

However, I failed to see the valid analysis on the mechanisms how growth regulates Ca^2+^ pattern. The mechanisms how Ca^2+^ regulates growth are unknown, but it is acceptable for such a more difficult question. The authors should at least clearly address the prior question with significant improvement on their arguments, as well as the readability. Here are my comments:

1. Although details of the computational model seem to be explained in the supplement, it would be nice to have a brief discussion in the main text to help readers understand the model better.

Response: In the revised manuscript, we have added a new section following the introduction where we describe the key details of the model formulation. The section explains the relevant equations used in our simulations and the rationale for the model based on our previous experimental work (1).

And many clarifications are needed:

a. Fig 1B-C: It is unclear what do the spatial axes X, Y and L represent in the wing disc.

Response: The X and Y coordinates in the Figure 1B correspond roughly to the anterior-posterior (A/P) and dorsal-ventral D/V axes of the wing disc. Similarly, in panel C the kymograph is drawn across the A/P axis. We have clarified this in the figure by denoting where the A/P and D/V axes are (panel B) and that the position goes from anterior to posterior in panel C. We more explicitly describe this in the revised figure caption.

b. Fig 1D: The meaning of each annotation should be further explained. Why GPCRs and RTKs use the same annotation, and same issue for PLCϒ and PLCβ. Both GJ and IP_3_R are represented by white box, are they the same thing?

Response: We have annotated individual molecules in Figure 1D based on the functionality of the molecules. GPCRs and RTKs are classes of cell surface receptors that can activate different classes of phospholipases (PLCs), and we have indicated this. Both PLCγ and PLCβ are two classes of PLCs that generate IP_3_ from hydrolysis of PIP_2_. We have revised the figure panel to explicitly show that both classes of PLCs are represented by the lumped parameter V_PLC_ which represents the sum of stimulated GPCRs that activate PLCβ and RTKs that activate PLCγ. Presumably, each PLC isoform have their own unique (but currently unknown) V_PLC_ and K_PLC_ values. Hence, Figure 1D shows that both GPCRs and RTKs produce IP_3_ where our model parameter V_PLC_ is the net sum of PLC activity. Thus, we have indicated them with similar annotation. As described in this report, the different input stimuli lead to differential spatiotemporal extents of calcium signaling, suggesting that Ca^2+^ signaling is multiplexed. GJ and IP_3_Rs are the molecules that allow for the transport of molecules.

c. Fig 1D and 1E: There are many regulatory mechanisms, especially for Ca^2+^. The regulatory mechanisms and their corresponding formulations need to be clearly explained.

Response: Done. We have added a new section under methods explaining the mathematical model formulation based on experimental results. We have also removed panel E from Figure 1, and it is expanded in the new section.

d. Is FEX concentration related to VPLC in the computational model? FEX and VPLC levels are shown in different colors in Fig 1 and Fig 2. It may be good to point out they are the same thing. Maybe using the same color or adding some extra texts in the figures to highlight it?

Response: Yes, FEX in Figure 1 refers to fly extract (agonist) concentration added to the culture media. We deduce that fly extract (FEX) contains an unknown stimulator of phospholipase β activity. Thus, increasing FEX concentration corresponds to an increasing value of V_PLC_ for all cells in the model. There is cell-cell heterogeneity of V_PLC_ in unstimulated cells, including a small fraction of initiator cells exhibiting higher V_PLC_.

In the computational model, the agonist concentration is directly related to the proportion of cells with V_PLC_ values close to single-cell Hopf threshold. Thus, V_PLC_ is not *directly* a parameter representing the concentration of FEX, but is a parameter that describes the net activity of PLC through FEX stimulation of upstream receptor(s). In Figure 2A, we show that having cells below single cell Hopf bifurcation threshold (Fig 2E) results in spikes whereas as we increase the proportion of cells with V_PLC_ close to single cell Hopf bifurcation threshold we begin to observe Ca^2+^ waves in qualitative agreement with higher concentration of FEX. These waves resemble the Ca^2+^ waves that we observe experimentally (Figure 1B) when we stimulate the wing discs with 15% (v/v) FEX in the media. As the specific GPCR and GPCR ligands involved in the stimulation of wave activity remain to be discovered, the model does not explicitly model FEX in the wing disc cells. However, results from our computational simulations indicate that variation in total stimulation strength between individual cells is sufficient to cause changes in the Ca^2+^ signaling patterns.

e. It is unclear how GJ affect permeability of Ca^2+^ and IP_3_. Is it also a component in the equations or it is only a parameter for permeability?

Response: Gap junction permeability for Ca^2+^ ($F_{c}$) and IP_3_ ($F_{p}$) is a parameter in the equations

$$J_{p}\approx F_{p}\left[ \sum_{j\in N_{i}} p_{j}l_{j}-p_{j}\left( \sum_{j\in N_{j}} l_{j} \right) \right]$$

$$J_{c}\approx F_{c}\left[ \sum_{j\in N_{i}} c_{j}l_{j}-c_{j}\left( \sum_{j\in N_{j}} l_{j} \right) \right]$$

wherein F_p_ refers to the permeability of IP_3_ through gap junctional communication, F_c_ refers to the permeability of Ca^2+^ through gap junctional communication, $p_{j}$ and $c_{j}$ refer to IP_3_ concentration and Ca^2+^ concentration respectively in neighboring cell $j$ and $l_{j}$ refers to the length of cell boundary shared by cells $i$ and $j$ respectively.

Equations shown above assumes that each cell is well-mixed. Furthermore, the model neglects intracellular diffusion of Ca^2+^ and IP_3_. We assume that the flux of Ca^2+^ and IP_3_ through gap junctional proteins linearly proportional to the concentration difference between cell i and cell j. Thus, the equation describing intercellular flux is

$$J_{i-j}\mathcal{= F(}c_{i}-c_{j})$$

where $\mathcal{F}$ is the permeability coefficient for different species diffusing through gap junctions such as Ca^2+^ and IP_3_, $c_{i}$ and $c_{j}$ are concentrations of species in i and j cells that are connected by gap junctional proteins. We adopted this formulism from the works of Othmer and Scriven (2) and included this in the computational model section after the introduction.

f. In Fig 2A-2D, is it possible to show the four distinct patterns in the same way in Fig 3?

Response: We have changed 3D kymographs in Figure 2A-D to 2D kymographs in order to be consistent with other figures in the manuscript. We, however, feel that 3D kymographs clearly demonstrate the spikes that result when gap junctions are inhibited more so than the 2D kymographs. Hence, we have decided to keep the 3D visualization in Figure 3.

g. In Fig 2A-2D: It seems cell with V_PLC_ above the Hopf bifurcation is initiator cell. Fig 2E shows all cells are standby cells in Spikes, ICT and ICW, and all cells are initiator cells in fluttering. But why Fig 2A-2C still have initiator cells (I assume the dark blue points are initiator cells? May have an annotation pointing this out when the Input profile is presented). Also, statistical meaning of the bar and box in Fig 2E should be given in the figure legend.

Response: To clarify, there are initiator cells within the whole cell population in all our simulations in Figure 2A-2D. Figure 2A has been updated to indicate that dark blue points are initiator cells with high V_PLC_ values. In Figure 2A-C, the total fraction of initiator cells are less when compared with that of Figure 2D. We have additionally highlighted the small distribution of initiator cells for spikes, ICTs, and ICWs in Figure 2E. Our claim is that despite the same initiator cell V_PLC_ profiles (Figure 2A-D have the same initiator cell locations and V_PLC_ values), the proportion of standby cells close to the single cell Hopf threshold determines the class of the Ca^2+^ signaling activity observed in the wing disc. For instance, in the case of spikes, all the cells excluding the initiator cells have V_PLC_ values lower than the single cell Hopf threshold. In the case of ICWs, the proportion of cells with V_PLC_ higher than Hopf bifurcation threshold increases.

h. Is the tissue growth explicitly modeled or only modeled in various fixed size tissue?

Response: Our model does not model growth explicitly as this would be a completely separate work requiring further mechanistic studies beyond the scope and questions raised here. It would also require defining explicitly relationships between calcium signaling transients and other core growth pathways, which still need to be defined. Our imaging studies are performed over approximately 1 hour of development, while significant growth occurs only on the order of a day.

As a proxy, we instead looked at how Ca^2+^ signaling varies at different tissue sizes given the time scale differences of individual calcium transients and slow tissue growth dynamics. Using the model formulation framework described in Figure S1, we reduced the number of cells in a simulated tissue.

It is likely that the impact of Ca^2+^ signaling on growth must be integrated, but before we can answer specific questions on growth control, we first have to understand the regulation of short-medium term dynamics of minutes to hours. Nonetheless, we are able to tie the long term impact of specific classes of Ca^2+^ stimulation (either through GPCR/PLCβ or RTK/PLCγ) to changes in the final size of the organ. This in turn motivates the study into what governs the spatiotemporal dynamics of calcium signaling in this tissue. It also motivates future work to investigate how the classes of Ca^2+^ signaling dynamics tunes downstream gene expression and metabolic states of the tissue.

2. Line 314-343: the authors proposed two hypotheses to explain how wing disc growth regulates Ca^2+^ pattern. Then they used both computational model experiments to eliminate one hypothesis. However, I found this argument lacks of evidence.
   a. What is the difference observed in these two hypotheses in computational model? As I can tell, the only difference is that they have different numbers of initiator cells at initial point. I don’t understand how experiment helps eliminate one of them? How can experiment identify initiator cells? Experimental observations are shown in Fig 4C-4D, and where is the consistence stated in Line 337-339 being found comparing to the computational model?

Response: To clarify, we looked at changes of Ca^2+^ dynamics in different sized tissues. Previous Figure 4 had a mislabeled scaling bar in Panel B, and this has been corrected in the current version of figure 4. To explain more clearly, we explored two possible explanations of observed trends related to tissue size and Ca^2+^ signaling dynamics with *in silico* scenarios. In the first scenario, we considered the case of decreasing the total number of initiator cells as the development proceeds and the discs grow in size while maintaining a constant level of gap junction permeability (Figure 4A). We observed reduced number of waves and ICTS and a larger number of Ca^2+^ spikes in the larger discs and greater number of ICTs and fewer number of Ca^2+^ spikes in smaller discs. This observation is consistent with our earlier experimental observation we published in Brodskiy et al. wherein we reported an inverse relationship between Ca^2+^ signaling activity and the pouch size when discs were cultured in 15% (v/v) FEX. In the second scenario, we tested whether gap junctional communication scaling with size also explains the observed decrease in the number of spikes (Figure 4B). To do so, we held the number of initiator cells constant regardless of size but decreased the gap junctional permeability as the tissue size increased. We have further clarified using text in panels A and B to explicitly state what is held constant and what is varied. In Figure 4A, the total number of initiator cells is varied, gap junction communication is held constant (Figure 4A). In Figure 4B, the gap junction communication is varied while the number of initiator cells is held constant (Figure 4B).

When we decreased gap junction permeability in the wing discs, we observed similar trend where there was a decrease in total spikes as the discs grew in size. To experimentally differentiate between the two scenarios, we cultured wing sizes from larva with different age in media without agonist such as insulin and FEX and quantified the Ca^2+^ activity. Our rationale for performing such a study is to avoid overstimulating receptors on the wing disc by addition of FEX and insulin, and to mimic in vivo conditions ex vivo. In theory, not modelling agonists to either GPCRs and RTKs should indicate the number of initiator cells above the V_PLC_ Hopf bifurcation. When we cultured the wing discs in the absence of agonist, we observed only spikes. We did not observe ICWs and ICTs similar to what our model predicts for smaller wing discs. We report in the manuscript that the spikes observed in the absence of agonists have a similar Ca^2+^ dynamics to initiator cells observed in the simulations. Furthermore, when we characterize the number of spikes in differently sized wing discs, we observed a net increase in the number of spikes as disc size increases. However, when we scaled the spikes with size of the pouch, we did not observe a strong difference. This suggests that the initiator cells do not scale with size, but the total number of spikes increase suggesting that gap junctional communication likely decreases as the discs age leading to increased number of spikes.

b. How parameters or any other conditions are perturbed in the two cases for computational model? The relation between number of initiator cells and disc size, and the relation between permeability and disc size in two cases should be given.

Response: We have listed in the Figure 4 caption how initiator cell counts and gap junction communication is scaled. In Figure 4A, the total number of initiator cells was varied according to the following equation: $N_{initiators}=8,000\cdot N_{cells}^{-0.8}$ where $N_{cells}$ is the total number of cells in the simulated tissue. The power-law relationship power of -0.8 was used from the discovery that integrated Ca^2+^ intensity scales in a similar power-law fashion detailed in one of our previous publications (1). The constant in front of the equation, 8,000, was chosen to output physiologically realistic fractions of initiator cells with the simulated tissue sizes. In future studies, this value has the possibility of being fit to experimental data if fractions of initiator cells can be quantified. In Figure 4B, gap junction permeability was varied according to the following equation: $F_{p}=800\cdot N_{cells}^{-1.8}$ where $F_{c}$ is directly proportional to $F_{p}$ such that $F_{c}=0.1\cdot F_{p}$ and the total number of initiator cells is held constant in this scenario ($N_{initiators}=65)$. All other parameter values are those listed in the revised Table 1 of the main text. Further, in both scenarios the V_PLC_ values of all standby cells were restricted to values of 0.40 in order to attribute any differences in resulting Ca^2+^ activity to the scaling relationships, and not the V_PLC_ activities of the tissues. These details have been further expanded upon in the revised Figure 4 caption and included in the main text.

c. In the first scenario, how to get decreasing fraction of initiator cells? If it is manipulated by decreasing VPLC, different decreasing rate of VPLC may lead to different observations. The authors should give a more systematic analysis to show how generally decreased number of initiator cells leads to decreased Ca^2+^ activity.

Response: In order to obtain a decreasing number of initiator cells, the total count of initiator cells with V_PLC_ values above the Hopf bifurcation value was scaled with the total number of cells in the simulated tissue. The full equation is $N_{initiators}=8,000\cdot N_{cells}^{-0.8}$ where $N_{cells}$ is the total number of cells in the simulated tissue. The V_PLC_ profiles for all tissue sizes in Figure 4A have standby cells with V_PLC_ values restricted to values of 0.40, reflecting the low level of exogenous stimulation performed with experiments in Figure 4C, D. V_PLC_ profiles for standby cells in other regimes (*i.e.,* the spikes, ICW, or fluttering ranges) result in simulations that are not consistent with experimental results, thus 0.40 was used. We must stress that there is not decreasing V_PLC_ values in the simulations shown in Figure 4A as all standby cells are restricted to the value of 0.40 in each tissue size. The only variation between tissue sizes is the total number of initiator cells. The count of initiator cells (naturally spiking) are detected by imaging wing discs that are not stimulated with either fly extract or insulin. In the experiments, we see a slight decrease in number of spiking cells per tissue area over during the later stages of larval development consistent with declining numbers of initiator cells and / or levels of gap junction communication.

d. All conclusions from simulations should be made from multiple independent repeats and their statistics, instead of a single simulation.

Response: Done. For each of the figures in the main text (Figure 2, 3, and 4), we repeated the simulations for a total count of 5 simulations; each with a different random number generator seed. With different random number generator seeds, each simulation has different locations of initiator cells and standby cells (*i.e.,* completely different V_PLC_ profiles of tissues). Observation of these repeated simulations leads to the same results and the conclusions remain the same. The results of these repeated simulations are located in our GitHub repository here: <https://multicellularsystemslab.github.io/MSELab_Calcium_Cartography_2021/> where there is a section linking to code and corresponding output for the repeated simulations. We have also included a separate figure (Figure S13) quantifying the class of Ca^2+^ activity in each of the repeated simulations. All conclusions hold for multiple independent repeats.

3. Line 381-383: The authors only showed insulin affect Ca^2+^ functions. It is completely unknown how insulin interacts with the system the authors modeled. The perturbations on two parameters for mimicking experimental manipulation on insulin make no sense.

Response: Previous experimental studies in the *Drosophila* wing have shown that insulin signaling interacts with one of the PLC isoforms (PLC γ) homolog *sI* expressed in the wing disc (3). In our previous experimental paper, we also reported that knockdown of *sI* in the wing disc did not inhibit fly extract (FEX) induced waves, but knockdown of Gq and PLC β did induce waves (1). We have added this rationale to better explain our experiments.

Notably, when we increased insulin in the culture media we observed an increased number of single cell Ca^2+^ spikes which phenocopies Ca^2+^ spikes observed when we inhibit gap junctional proteins Inx2 (1). Another tie between insulin and PLC is that the insulin receptor is a receptor tyrosine kinase, which can activate PLCγ.

Taking these observations together, we hypothesized two different scenarios which we tested with our computational model. In the first scenario, we asked whether the biochemical properties of PLC$\gamma$ may be different than PLC$\beta$. When we ran the simulations by varying K_PLC_, we observed a decrease in frequency of the Ca^2+^ oscillations. However, we did not computationally observe single cell Ca^2+^ spikes which we observed in wing disc cultured in the presence of insulin (Figures S5C and S7B). In the second scenario, we tested whether inhibition of gap junctions was sufficient to produce Ca^2+^ spikes (Figure S7A). When we ran the simulations setting gap junctional permeability to 0, we did observe single cell spikes similar to what we saw when discs were cultured in the presence of insulin. Only under conditions of severely reduced gap junction permeability are in silico spikes demonstrated with respect to these two scenarios (Figure S10).

It is important to note that our model does not explicitly model insulin signaling components. We only varied downstream effects of PLC-based stimulation of IP_3_ production and gap junction communication. This suggests that there is important future work needed to explore how insulin may regulate gap junction communication and quantify differences in the in vivo biochemical parameters for different PLC isoforms related to insulin and GPCR signaling pathways. Such an investigation is beyond the scope of this current work.

4. Many mistakes and missing information throughout this entire manuscript:
   a. Figure numbers are incorrect in the last two sections in Results, such as Line 3 5 0 , Line 355, line 372, etc.

Response: In the revised manuscript, we have changed the figure numbers.

b. Line 194: Something wrong in this equation.

Response: Fixed. $\tau$ should’ve been $\tau_{max}$ and we have fixed this in the revised manuscript. We modified the equation for rate of inactivation of IP_3_R, $r$, to match experimentally observed frequency and width of half max of the Ca^2+^ oscillations in the wing disc. We adopted the formulism proposed by Sneyd and colleagues (4). Detailed description of the model is given in the supplemental information.

c. Information for Tissue model is not given in Supporting Information (Line 575).

Response: We have added descriptions of how we constructed the tissue model from images of disc pouch of Ecad::GFP line to the revised manuscript. It is located in the “Tissue model” section of the materials and methods of the main text and Figure S1 of the supporting information.

d. Information for multicellular model is not given in Supporting Information as stated in Line 579-580.

Response: We have added a new section justifying our model formulation to the main text after the introduction.

e. Line 552: how many discs were collected and quantified for simulations? In simulation, how many real discs were used to calculate one computational domain?

Response: We used one wing disc labelled with E-Cadherin::GFP to reconstruct apical topology of the wing disc that we use as a prototypical disc for computational model.

f. ctot value is not given in Table S1.

Response: Fixed. We have moved Table S1 to the main text as Table 1 and included the value for C_tot_.

5. I don’t understand what was summarized in Fig 5H. Arrows point to everywhere without any specific meaning.

Response: Thank you for the feedback. We have accordingly clarified the figure and reduced the number of arrows in Figure 5H. Figure 5H is the “punchline figure” summarizing main findings of the paper. Briefly, cytosolic Ca^2+^ transients are stimulated by two different classes of cell surface receptors in the wing disc. First, it can be stimulated by insulin receptors which belong to the family of receptor tyrosine kinase (RTKs) and GPCRs to activate Gαq proteins and PLC β respectively. Second, we show that stimulation of RTKs and GPCRs result in different spatiotemporal patterns of Ca^2+^ due to different levels of stimulation of initiator cells and the standby cells. In the case of insulin stimulation, we observe Ca^2+^ spikes. The computational model predicts that V_PLC_ of standby cells is way below Hopf bifurcation threshold. Furthermore, Gαq overexpression in the wing disc leads to Ca^2+^ waves implying that majority of standby cells are close to Hopf bifurcation. These two different Ca^2+^ patterns are mapped to two different growth responses. Insulin signaling produces predominantly Ca^2+^ spikes and stimulates growth of the final wing size. Increased levels of GPCR/Gαq/PLC$\beta$ signaling leads to persistent Ca^2+^ waves and a reduction in the wing area. Arrows in the figure highlight these processes and the number of arrows has been reduced.

6. Many aspects of the model formation need to be clarified:
   a. In the computational model, only randomness in PLC production is considered while other components are in deterministic description. Any rationale? The Ca^2+^ concentration may also be largely perturbed in different cells.

Response: V_PLC_ is the stimulation-dependent control parameter in our model. Our previous experimental reports showed that the concentration of stimulus (FEX) added to the culture media determined the observed class of Ca^2+^ activity (1). As the driving factor in recapitulating these patterns in silico, we varied single cell V_PLC_ values. We do, however, acknowledge that other parameters could potentially affect Ca^2+^ signaling patterns including kinetics of degradation of IP_3_ in the system. However, our objective of this study is to establish single cell properties that affect the pattern behavior and to experimentally elucidate the physiological functions of these patterns in wing development.

b. In the single-cell model, only IP_3_ has degradation rate. Any explanations why degradation is not considered for IP_3_R and Ca^2+^? From the value of k_5,p_, The half-life for IP_3_ is 0.46 sec, which is extremely fast, explanation? The degradation term for p in Fig 1E should be negative.

Response: Our model assumes that IP_3_R proteins to be more stable. We adopted the formulism proposed by De Young and Keizer to describe IP_3_R dynamics (5). The model considers the changes to the state of the IP_3_Rs in our equations. Our model assumes that IP_3_R has three equivalent and independent subunits and each subunit has IP_3_ binding site, an activating Ca^2+^ binding site and an inactivating Ca^2+^ binding site respectively. IP_3_Rs could thus be in one of the eight states. Our model assumes that the rate of inactivation of IP_3_R occurs at a slower time scale than the activation of IP_3_Rs by IP_3_ and Ca^2+^. We, therefore, describe IP_3_R inactivation as a separate ODE. Ca^2+^ cannot be generated or degraded but can move in and out of the cell’s cytoplasm.

In the case of Ca^2+^, our model considers transport of Ca^2+^ from ER through IP_3_R and ER. Our model also considers binding of Ca^2+^ to IP_3_R Ca^2+^ binding sites and SERCA pumps. IP_3_ is rapidly metabolized by other kinases and phosphatases in the cytosol yielding fast kinetics.

We have fixed the typo (negative sign) in front of the degradation term.

c. Equation c for Cytosolic [Ca^2+^] in Fig 1E, it is not common to have a cube (second term) on the Michaelis-Menten term. Any interpretation?

Response: Ca^2+^ flux through IP_3_R can be described using Markov model wherein the state of IP_3_R in eight possible states is considered. Our current model computes the probability of the IP_3_Rs in the “open” state wherein the IP_3_R is bound to IP_3_ and Ca^2+^ in the activating domain. Cubic term in the equation stems from simplifying the eight state Markov model of IP_3_R to include only states that correspond to “open” state of the receptor. This simplification stems from the work of Li and Renzel and detailed derivations are reported in these references (6).

d. In Table S1, authors should point out clearly the source for each parameter. If the parameter is from this work, how they were selected should be stated. A systematic sensitivity analysis for all parameter is necessary.

Response: In the revised manuscript, we have moved Table S1 to be Table 1 in the main text. We have additionally pointed out the source of each parameter. We have also performed sensitivity analysis of other parameters and included them in revised Figures S4 – S6.

e. In one simulation, is the value of VPLC randomly perturbed in each cell, and is it dynamically perturbed during the simulation or only at the initial state? The ranges of VPLC in each phenotype are not pointed out in Table S1 (as stated in line 198).

Response: In all the simulations, V_PLC_ profiles for all of the cells are generated from random uniform distributions from a range of values depending on the in silico experimental setup. Initiator cells indexes/locations in the 2D tissue are randomly selected at the start of the simulation using a random number generator seed. The code is constructed in such a way that we can have consistent initiator cell indexes/locations by selecting the same random number generator seed. Initiator cell V_PLC_ values are randomly uniformly distributed from 1.3 to 1.5 in the simulations (aside from having an exact value of 0.774 if Figure 3). Cell indexes/locations not selected to be initiator cells are set as standby cells and their V_PLC_ values are randomly uniformly distributed within the code using a modifiable lower and upper bound. For example, in Figure 2A-D, the bounds of standby cell V_PLC_ values for spikes was 0.1 – 0.5, ICTs was 0.25 – 0.60, ICWs was 0.4-0.8, and fluttering was 1.4 – 1.5. Simulated tissues for Figures 2 and 3 had 25 initiator cells in each simulation. Simulated tissues in Figure 4A had a scaling fraction of initiator cells while Figure 4B all had constant initiator cell counts of 65.

We have more explicitly stated this in the revised text. We do not alter the V_PLC_ values dynamically. We have also listed the range of V_PLC_ values testing in all the simulations in Table 1 of the main text. However, the specific V_PLC_ ranges corresponding to the reproduced four calcium patterns for initiator cells and standby cells is given in Figure 2E.

7. Explanations on functions of each experimental manipulation would be helpful to readers, such as expression region for each GAL4/UAS systems and function of GCaMP6f sensor.

Response: We have added a new figure in the supplementary information (Figure S8) illustrating the expression regions of the transgenes in the wing disk along with an explanation of GAL4/UAS system.

8. When the authors point to Supporting Information, it would be nice to point out the specific sections.

Response: In the revised manuscript, we have pointed out specific sections and more explicitly labeled supporting information figure references.

**Reviewer # 2**

The authors present a systems-level description of Ca^2+^ signaling during organ development which has been lacking in the literature. This is an important contribution since Ca^2+^ signaling plays a crucial role in development but not enough modelling has been done in this direction to elucidate many open questions. The authors conduct a bifurcation analysis/parameter sensitivity analysis of the model by altering key parameters and find four types of Ca^2+^ signals which are also observed in experiments. I recommend publication with minor revisions. I list the revisions requested below:

Response: We thank Reviewer #2 for a strong recommendation on the value of this work.

- L62-63: I find very interesting the finding “Our experiments also reveal that a “goldilocks zone” of optimal Ca^2+^ activity is required to achieve optimal growth at the organ level”. The same optimal window has been observed in other Ca^2+^ signaling scenarios, for example for fertilizing the egg successfully. Maybe refer to this phenomenon more generally in other parts of the text.

Response: We have cited a review article describing mechanisms of Ca^2+^ activation during fertilization (7). The mechanism of Ca^2+^ signaling has striking differences with the Ca^2+^ signaling observed when egg is fertilized. For instance, various studies have shown that PLC isoform PLC ζ triggers Ca^2+^ release form the Ca^2+^ stores such as the ER. In contrast, we have experimentally shown that PLCβ homolog PLC 21C is required for Ca^2+^ oscillations in the wing imaginal disc. Next, Ca^2+^ activation in the egg has been shown to be coordinated with increased exocytosis in some species. Whether this is indeed the case in our system is not known and warrants further investigation.

- L81: “Epithelial cells do not observe social distancing” – although I found this sentence amusing in a way as it is obviously related to the pandemic, I also find it detracting from the context of the embryogenesis

Response: We have modified this sentence as our aim was merely to grab the reader’s attention rather than detract from the context of organogenesis. We have changed it to “Mechanisms of intercellular communication are critical during epithelial morphogenesis when cells communicate and coordinate their activities to generate functioning organs (1,2).”

- L.192: “We modified the rate of IP_3_R inactivation term to replicate our experimental Ca^2+^ dynamics”. Some more explanations to be provided about the modelling process here and also in the Supplementary Information where the model is explained. How was the particular choice of the model and the parameters chosen motivated by your experiments? More specifically, please justify in more detail why the Ca^2+^ signaling model by Politi et al (2006) was chosen versus many other options. Does it provide the full spectrum of qualitative behaviors observed in experiments perhaps?

Response: Our choice of mechanistic mathematical model was based on the experimental interrogation of fly extract (FEX)-induced Ca^2+^ signaling that we and other groups have published previously (1,10,11). We and others that mapped out key molecular plays that regulate Ca^2+^ signaling in response to FEX and other mechanical stimulators including IP_3_R, PLCs and Gαq. Specific receptors involved in the signaling transduction machinery is being mapped out in a separate study in our lab. Given this information, we feel that the model proposed by Politi describing Ca^2+^ signaling in single cells adequately describes the Ca^2+^ signaling we experimentally observe. This is due to the fact that the Politi model describes hormone stimulated Ca^2+^ signaling and includes relevant feedback loops between IP_3_ and Ca^2+^ on key molecules such as PLC and IP_3_R. Previous published experimental studies on single channel properties of the IP_3_Rs does show the possibility of these feedback loop described by the models (12). To better illustrate these points, we have included a revised model description as a new section under computational methods of the main text. We find it striking that we did not have to significantly vary model parameter values to capture much of the experimentally observed phenomena, suggesting strong preservation of biochemical features across a broad range of biological contexts.

- L. 198 (and earlier): “To reproduce the four distinct patterns in silico, we varied the VPLC parameter progressively in individual cells across a range of parameters (Table S1).” Since VPLC is a very important bifurcation parameter I strongly suggest to explain it in more detail in the main text.

Response: V_PLC_ refers to the maximum production rate of IP_3_ in the cells and represents the lumped activity of stimulated Phospholipases. In the Politi model, this parameter is dependent on the concentration of the hormones and growth factors added to the cell culture (13). Descriptions of this term is given in the Computational methods section in the main text respectively.

- L. 250: “VPLC values set to the Hopf bifurcation threshold value of 0.774”. Please explain where 0.774 is coming from/why it is chosen.

Response: The Hopf threshold was identified from a single cell version of the model wherein Ca^2+^ oscillations occur in the cell when V_PLC_ is at or above the value of 0.774 (Revised Figures 2F, S2A). This is further confirmed in a bifurcation analysis of the V_PLC_ parameter (Revised Figure S3). We used single cell model to calibrate our system to match the experimentally observed oscillation frequency. We then generalized the single cell model to the entire wing disc by including the Ca^2+^ and IP_3_ diffusion terms across the network of cells.

- Figure S4: In the bifurcation analysis why only V_PLC and F_P chosen as bifurcation parameters. Could you also add bifurcation diagrams for τ_max, F_c, K_τ? (If there is a reason for not including these bifurcation diagrams please state it clearly.) Also, a 2-parameter bifurcation diagram could be useful.

Response: Our objective behind the construction of bifurcation diagram is to rationally determine ranges of parameter values for V_PLC_ term for the 2D model. We emphasize the V_PLC_ term because it is the term used by Politi and many others as the parameter that depends on the agonist concentration in the media. We varied this parameter in order to determine agonist conditions that leads to various Ca^2+^ patterns. Other terms in the equations such as $\tau_{\max}$ and $k_{\tau}$ are not dependent on agonist stimulus and thus we have not performed bifurcation analysis.

- Please provide some more details about the algorithm/numerics of the cell-based model you have developed (in Supplementary Information). Also, it would be good to provide some more instructions so that the Python code can be easily run by other users (I have followed the GitHub link provided and read the “Readme” but have not run the code.)

Response: We have included a section in the materials and methods labeled “Tissue model” where we describe how the cell-based model was developed using EpiTools We have updated the [GitHub webpage](https://multicellularsystemslab.github.io/MSELab_Calcium_Cartography_2021/) to provide a link to a Google Colaboratory script that is able to run a demo simulation that corresponds to Figure 2 of the main text where you can select to simulate single cell spikes, intercellular transients, intercellular waves, or global fluttering. For convenience, you can also find the link to the Google Colaboratory script [here](https://colab.research.google.com/drive/1Md-OkLtQ3TdeEt3aBj__tua_hnYCPsdj?usp=sharing) (https://colab.research.google.com/drive/1Md-OkLtQ3TdeEt3aBj__tua_hnYCPsdj?usp=sharing). We have also more explicitly written instructions on how to run the code for new users by providing images and step-by-step details of downloading and running the code.

**Typographical errors and aesthetic/stylistic edits:**

In the abstract: Ca 2+ -> Ca^ {2+} as in main text Recently we showed - > We have shown recently

Response: We have changed the phrase “Recently we showed” to “We have shown recently” in the abstract.

When many references are listed, e.g. L106-7 please make sure they are in chronological order (or reverse order, depending on what the journal requires)

DONE. We have updated the all the references in Vancouver format as per the guidelines in the PLoS Computational Biology website.

L121 and L123 “not characterized” appears in subsequent sentences, please choose a different phrase

Response: We have changed the phrase “not characterized” to “poorly understood”.

L123: IP_3_R -> IP_3_?

Response: We have changed IP_3_R to IP_3_ in the main text.

L143: summary of baseline -> summary of the baseline

Response: In the revised manuscript, we have decided to completely remove panel E from the figure in an effort to explain the equations more thoroughly in the newly incorporated “Computational Model” section under methods.

L. 194: the subscript “max” is missing in τ (See equation)

Response: We apologize for this typo. It was a formatting issues that came up when we converted our document to PDF. We have fixed it in the revised manuscript.

**References:**

1.	Brodskiy PA, Wu Q, Soundarrajan DK, Huizar FJ, Chen J, Liang P, et al. Decoding calcium signaling dynamics during *Drosophila* wing disc development. Biophysical journal. 2019;116(4):725–40.

2.	Othmer HG, Scriven LE. Instability and dynamic pattern in cellular networks. Journal of Theoretical Biology. 1971 Sep 1;32(3):507–37.

3.	Murillo-Maldonado JM, Zeineddine FB, Stock R, Thackeray J, Riesgo-Escovar JR. Insulin Receptor-Mediated Signaling via Phospholipase C-γ Regulates Growth and Differentiation in *Drosophila*. PLOS ONE. 2011 Nov 21;6(11):e28067.

4.	Sneyd J, Han JM, Wang L, Chen J, Yang X, Tanimura A, et al. On the dynamical structure of calcium oscillations. PNAS. 2017 Feb 14;114(7):1456–61.

5.	De Young GW, Keizer J. A single-pool inositol 1,4,5-trisphosphate-receptor-based model for agonist-stimulated oscillations in Ca^2+^ concentration. Proc Natl Acad Sci U S A. 1992 Oct 15;89(20):9895–9.

6.	Li Y-X, Rinzel J. Equations for InsP3 Receptor-mediated [Ca2+]i Oscillations Derived from a Detailed Kinetic Model: A Hodgkin-Huxley Like Formalism. Journal of Theoretical Biology. 1994 Feb 21;166(4):461–73.

7.	Swann K, Lai FA. Egg Activation at Fertilization by a Soluble Sperm Protein. Physiological Reviews. 2016 Jan 1;96(1):127–49.

8.	Leptin M. Drosophila Gastrulation: From Pattern Formation to Morphogenesis. Annual Review of Cell and Developmental Biology. 1995;11(1):189–212.

9.	Schöck F, Perrimon N. Molecular Mechanisms of Epithelial Morphogenesis. Annual Review of Cell and Developmental Biology. 2002;18(1):463–93.

10.	Balaji R, Bielmeier C, Harz H, Bates J, Stadler C, Hildebrand A, et al. Calcium spikes, waves and oscillations in a large, patterned epithelial tissue. Scientific reports. 2017;7:42786.

11.	Restrepo S, Basler K. *Drosophila* wing imaginal discs respond to mechanical injury via slow InsP 3 R-mediated intercellular calcium waves. Nature communications. 2016;7:12450.

12.	Srikanth S, Wang Z, Tu H, Nair S, Mathew MK, Hasan G, et al. Functional Properties of the *Drosophila melanogaster* Inositol 1,4,5-Trisphosphate Receptor Mutants. Biophysical Journal. 2004 Jun 1;86(6):3634–46.

13.	Politi A, Gaspers LD, Thomas AP, Höfer T. Models of IP_3_ and Ca^2+^ oscillations: frequency encoding and identification of underlying feedbacks. Biophysical journal. 2006;90(9):3120–33.
