# Supplementary material for: From spikes to intercellular waves: Tuning intercellular calcium signaling dynamics modulates organ size control
Source: PLoS Comput Biol. 2021 Nov 1;17(11):e1009543. doi: 10.1371/journal.pcbi.1009543 (PMC8601605; doi:10.1371/journal.pcbi.1009543)
Supplement: Attachment — Submitted filename: PLoS_CB_ICW_Rebuttal_Letter_Second_Round_V3.docx [file pcbi.1009543.s038.docx]

**Review:** From spikes to intercellular waves: tuning intercellular Ca^2+^ signaling dynamics modulates organ size control.

The authors present a systems-level description of Ca^2+^ signaling during organ development which has been lacking in the literature. This is an important contribution since Ca^2+^ signaling plays a crucial role in development but not enough modelling has been Corrected. in this direction to elucidate many open questions. The authors conduct a bifurcation analysis/parameter sensitivity analysis of the model by altering key parameters and find four types of Ca^2+^ signals which are also observed in experiments. I am pleased to see that the authors incorporated successfully changes addressing my previous review and that of the other reviewer. I still recommend publication with (very) minor revisions. I list the new revisions requested below. (I recommend that the authors, once they implement the changes below, that they do not need to send back the manuscript for a further check.)

We thank the reviewer for positive assessment of the manuscript. We have addressed the minor revisions raised by the reviewer.

**ABSTRACT:**

1. “Our computational model successfully recapitulates how the dynamics of Ca^2+^ transients vary during organ growth” Could you use “reproduces” instead of “recapitulates”?

We have changed the word “recapitulates” to “reproduces” based on the comment from the reviewer

**MAIN TEXT:**

2. L. 183 “we ~~assume~~ only consider the case…”

We have deleted the word “assume”

3. p.192: the sentence “where Fp refers to the permeability of IP3 through gap junction communication,” does not make complete sense. You could say the “IP3 permeability of the gap junctions” – see also https://www.sciencedirect.com/science/article/pii/S0005273604000367?via%3Dihub if it helps.

We have changed the phrase to “IP_3_ permeability of the gap junctions” based on reviewer feedback.

4. p.193: “𝑙𝑗 refers to the length of the cell boundary shared by cells i and cell j, respectively.” The notation does not make complete sense to me – when you involve two cells it is natural that the subscripts of l should reflect that, i.e. shouldn’t you use l_{ij}?

We have changed $l_{j}$ to $l_{ij}$ to be more explicit in stating it is the length of the boundary shared by the cells. To further clarify distinctions in equations, we have modified them in the text to contain subscripts with reference to cell *i*. This required changing one of the parameters $K_{i}$ to $K_{r}$ throughout the text and supplementary information to avoid confusion.

5. L.201: “In our model, we ignore the flux of Ca^2+^ through the cell’s plasma membrane,” – please provide justification for this assumption

We have provided justification based on experimental studies. Experimental interrogations have suggested that the observed Ca^2+^ dynamics is primarily through Ca^2+^ release from ER through IP_3_R. Thus, to recapitulate this behavior in our model we have assumed that Ca^2+^ release from plasma membrane is negligible.

6. Please add English language articles where needed, such as “the” in many sentences. Examples (my correction with red):

• L. 178 “which is directly proportional to the PLC substrate PIP2”

Corrected.

• L.192-193: “…p𝑗 refers to the IP3 concentration in a neighboring cell j”

Corrected.

• L193: “lj refers to the length of the cell boundary”

Corrected.

• L.199: “pumped out from the cytosol to the extracellular…”

Corrected.

7. Please add punctuation marks in equations, e.g a comma is needed after equations when the sentence continues after the equation. For example, after equations (4), (5), (6), (7). Also please fix the capital in “Where” right after the power law for the initiator cells.

Corrected.

8. L.254: wholistic -> holistic

Corrected.

9. L. 244-245, L. 259-264

It is initially mentioned that the transition from limited to tissue-wide calcium activity depends on the amount of fly extract (FEX) added to the culture but then you vary VPLC. Please explain the dependence of VPLC on the amount of FEX more precisely, this is still not clear in L. 259- 264

We have further clarified the connect between FEX and V_PLC_ in revised lines L263 – 268 wherein FEX contains a mixture of agonists that stimulates PLC activity. The increased PLC activity would subsequently increase the maximal rate of production of IP_3_, which is the definition of V_PLC_. Therefore, by varying V_PLC_ we are indirectly simulating different concentrations of FEX with V_PLC_ and FEX being positively correlated with one another.

10. L. 286 “progression” is not the right word here. You could replace the bold-faced sentence in the beginning of the caption of Figure 2 as follows: “The level of hormonal stimulation governs the spatial extent of intercellular Ca2+ 286 communication”

We have replaced the caption as per the reviewer’s suggestion.

11. please resolve inconsistencies in “single-cell” vs “single cell”. When single cell is used as a noun it does take a hyphen (e.g. in L340 please remove the hyphen). When it is used as an advective it does take a hyphen, e.g. L.55 “single-cell spikes”

Corrected.

12. L. 346 “necessary to generating generate Ca^2+^ activity in (embryonic epithelial?) cells.” Or is this a phenomenon that you assume exists in other cell types?

We are unaware of this phenomenon occurring in other cell types. However, we believe that this phenomenon explains generation of single cell Ca^2+^ spikes in the absence of any agonist in wing disc cells modelled in this study.

13. L. 369: “Corrected.” -> “taken”

Corrected.

14. L. 371-372: “Decreased GJ permeability decreased propagation of the Ca^2+^ signal

across the simulated tissue whereas increased GJ communication increased

propagation.” The second or first part of this sentence is redundant as they relay the same point.

We fixed the sentence, which now reads: “We discovered that decreased GJ permeability decreased propagation of the Ca^2+^ signal across the simulated tissue.”

15. L. 376 and L. 378: please quantify “substantial” using percentages.

We have quantified these in Fig S7 where the percentage values in the figure are representative of a fixed multiplier (*i.e.*, 10% on the figure indicates 0.10 * Baseline). We have additionally modified the sentence to state “a 90% decrease” and a “100% increase” to quantify the simulation setup.

16. L. 379: please adopt consistent notation you had used “GJ permeability” earlier in the

text for “gap junctional permeability”. Also in L. 410 you use “Gap junction permeability”.

We have fixed this notation in throughout the text.

17. L.410: “shapes” ->”modulates”

Corrected.

18. “the power-law relationship value of -1.8 in scaling gap junction communication was

chosen to reflect the power-law relationship in our previous publication” please briefly remind the reader the reason for this choice, it is not obvious.

We have modified the sentence to clarify that -1.8 was chosen as an analogy to the negative power law correlation we observed between integrated Ca^2+^ activity and the pouch size from the experimental data, which we reported in our previous experimental paper.

The corrected sentence is as follows: “Similar to the scenario where initiator cell count was scaled, we investigated a scenario of scaling gap junction communication. We selected a power-law relationship value of -1.8 as an analogy to the relationship between integrated Ca^2+^ activity we reported previously (19).”

19. L. 435 “...activities of the tissues” -> why are these called activities? Please be consistent about the way you refer to V_{PLC} in the text. I also think it should be “of tissue”.

We have updated the text in revised lines L436 – 438 to clarify the simulation setup was Corrected. to ensure consistency across the two scaling relationship hypotheses.
